# Synthesis of novel pyrazole derivatives and neuroprotective effect investigation

**DOI:** 10.1080/14756366.2025.2583820

**Published:** 2025-11-12

**Authors:** Aihua Feng, Qing Zeng, Jun Wang, Han Li, Xu Fang, Yan Geng, Wei Pan, Gang Li, Junfeng Dong

**Affiliations:** ^a^ The First Dongguan Affiliated Hospital, Guangdong Medical University, Dongguan, China; ^b^ School of Pharmacy and Food Engineering, Wuyi University, Jiangmen, China

**Keywords:** Anti-inflammatory, pyrazole, spinal cord injuries, neuroprotective

## Abstract

Spinal cord injuries (SCIs) cause irreversible damage and lasting neurological impairments. Current treatments are limited to surgical and pharmaceutical interventions, underscoring the need for novel agents. In this study, 27 novel pyrazole derivatives were designed, synthesised. The anti-inflammatory and antioxidant activities of the compounds were systematically evaluated utilising lipopolysaccharide-stimulated BV2 microglial cells. Anti-inflammatory activity was assessed by quantifying the mRNA expression levels of key pro-inflammatory cytokines [tumour necrosis factor-α, interleukin-1β, and interleukin-6 (IL-6)] via quantitative reverse transcription polymerase chain reaction. Among the synthesised derivatives, compound **6g** demonstrated the most potent anti-inflammatory effect, exhibiting an IC_50_ value of 9.562 μM for the suppression of IL-6 expression and no significant cytotoxicity was observed. Notable, compound **6g** exhibited better inhibitory potency against IL-6 expression compared to the anti-inflammatory drugs dexamethasone and Celecoxib. These findings strongly support the potential of compound **6g** as a promising therapeutic candidate for mitigating secondary inflammation in SCI.

## Graphical abstract

**Figure:**
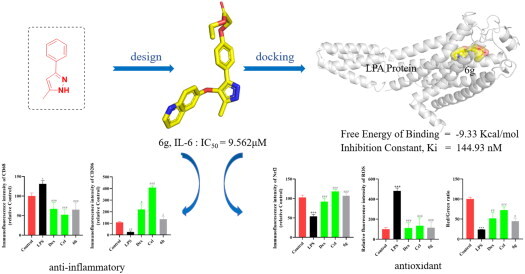

## Introduction

The spinal cord injury (SCI) refers to damage to the spinal cord resulting in either temporary or permanent dysfunction, which is major cause of disability and mortality across the world.^1–3^ The spinal cord undergoes irreversible damage due to physiological, biochemical, and structural alterations that occur following a SCI, which results in a high incidence of disability and mortality, imposing long-lasting psychological strain and significant financial pressures on both patients and their families.^4^^,^^5^ Currently, the primary treatments for SCI includes surgical decompression, pharmacological therapy, hyperbaric oxygen treatment, and prosthetic rehabilitation, etc.^6^^,^^7^ However, the current treatment outcomes are not satisfactory due to the intricate pathophysiological processes and microenvironmental imbalances involved.^8^ These pathophysiologic processes are divided into primary and secondary injuries, with the primary injury being irreversible.^9^^,^^10^ Secondary injury exhibits signs such as free radical generation, post-traumatic inflammatory response, oedema, programmed cell death, and glutamate excitotoxicity.^11^^,^^12^ Consequently, managing secondary injury following SCI may be the key to effective treatment and resolution of SCI.

Inflammation represents the most important characteristic sign of SCI, triggering the secretion of cytokines released by central nervous system or inflammatory cells, i.e. tumour necrosis factor-α (TNF-α), interleukin-1β (IL-1β) and interleukin-6 (IL-6).^13^^,^^14^ It has been discovered that reducing the anti-inflammatory capacity could alleviate neurological dysfunction from SCI.^15^^,^^16^ Oxidative stress is a major factor influencing the prognosis of SCI.^17^ The excessive release of Reactive oxygen species (ROS) can disrupt the balance and cause oxidative stress.^18^ The occurrence of oxidative stress destroys the subcellular structure of neurons and glial cells, leading to cell death.^19^ Therapeutic strategies that effectively remove ROS and alleviate inflammation may be the key to accelerating patient recovery. Therefore, the current study focuses on the development of novel anti-inflammatory agents specifically targeted at SCI.

**Figure 1.**
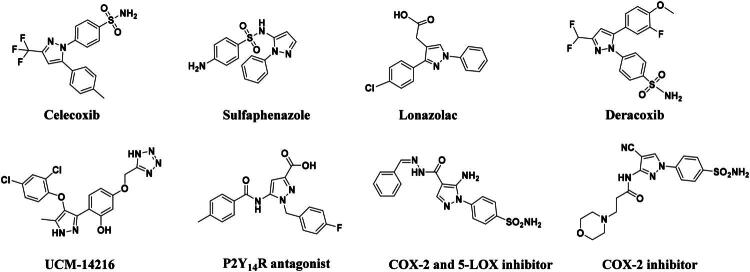
The representative anti-inflammatory agents based on pyrazole group.

**Figure 2.**
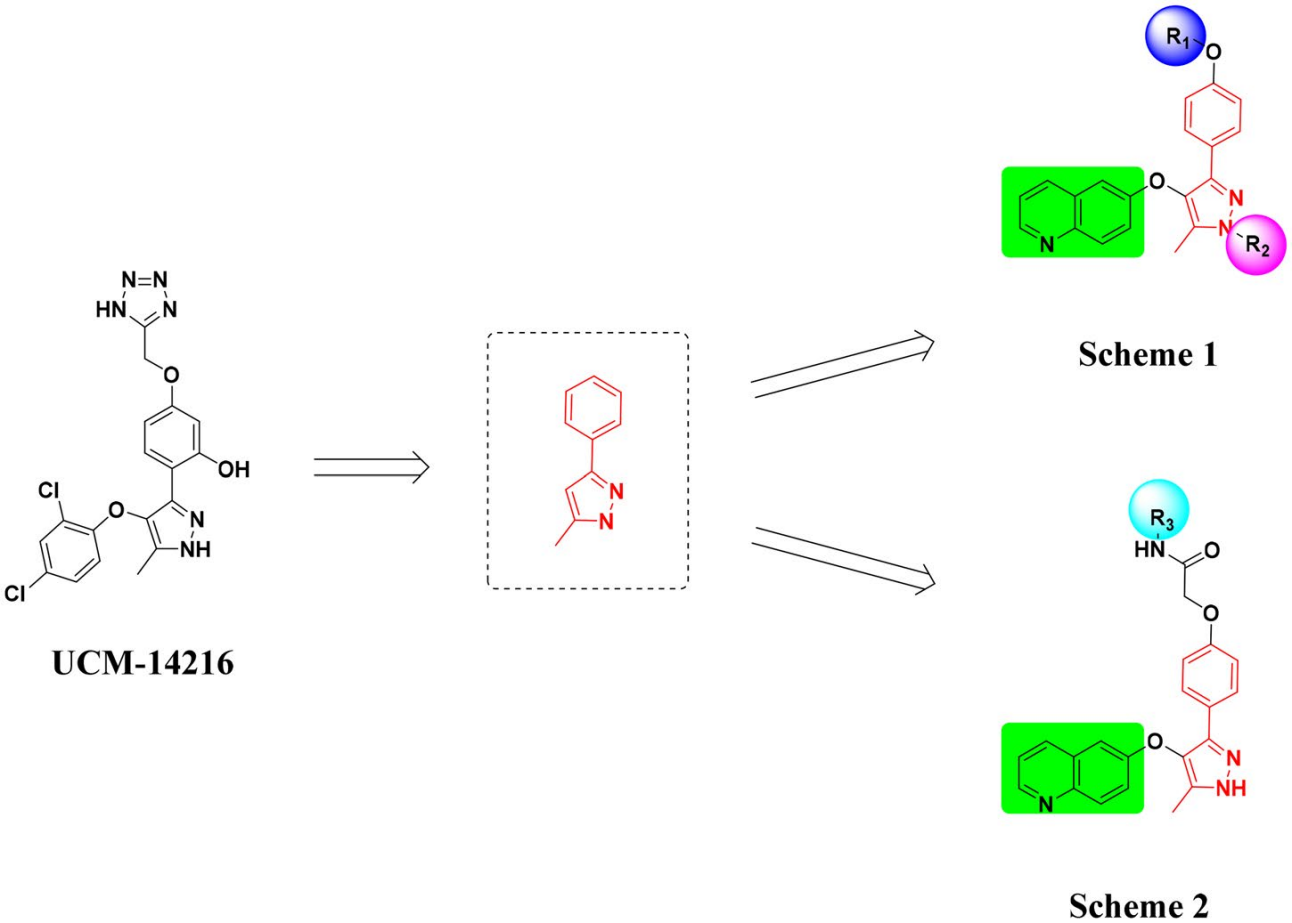
Design ideas.

The pyrazole group is widely used in medicinal chemistry and can be found in many drugs, such as anti-inflammatory,^20–23^ anti-cancer,^24^^,^^25^ anti-bacterial,^26–28^ anti-viral,^29^^,^^30^ and anti-fungal drugs.^31^^,^^32^ Numerous anti-inflammatory drugs are based on the pyrazole nucleus as shown in Figure 1. Celecoxib (Cel) is a well-known nonsteroidal anti-inflammatory drug and cyclooxygenase-2 (COX-2) inhibitor based on the pyrazole group,^33^ exhibiting strong anti-inflammatory effects and the ability to alleviate the sequelae of SCI. Among these compounds, we selected UCM-14216 as our lead compound, which is a potent and selective lysophosphatidic acid receptor 2 (LPA2) antagonist with an half maximal inhibitory concentration (IC_50_) of 1.9 μM,^21^ has demonstrated promising efficacy in a mouse model of SCI. In the present study, retaining the core pharmacophore of UCM-14216, we designed and synthesised 27 novel pyrazole derivatives and evaluated their anti-inflammatory and antioxidant activities (Figure 2).

## Results and discussion

### Chemistry

**Scheme 1.**
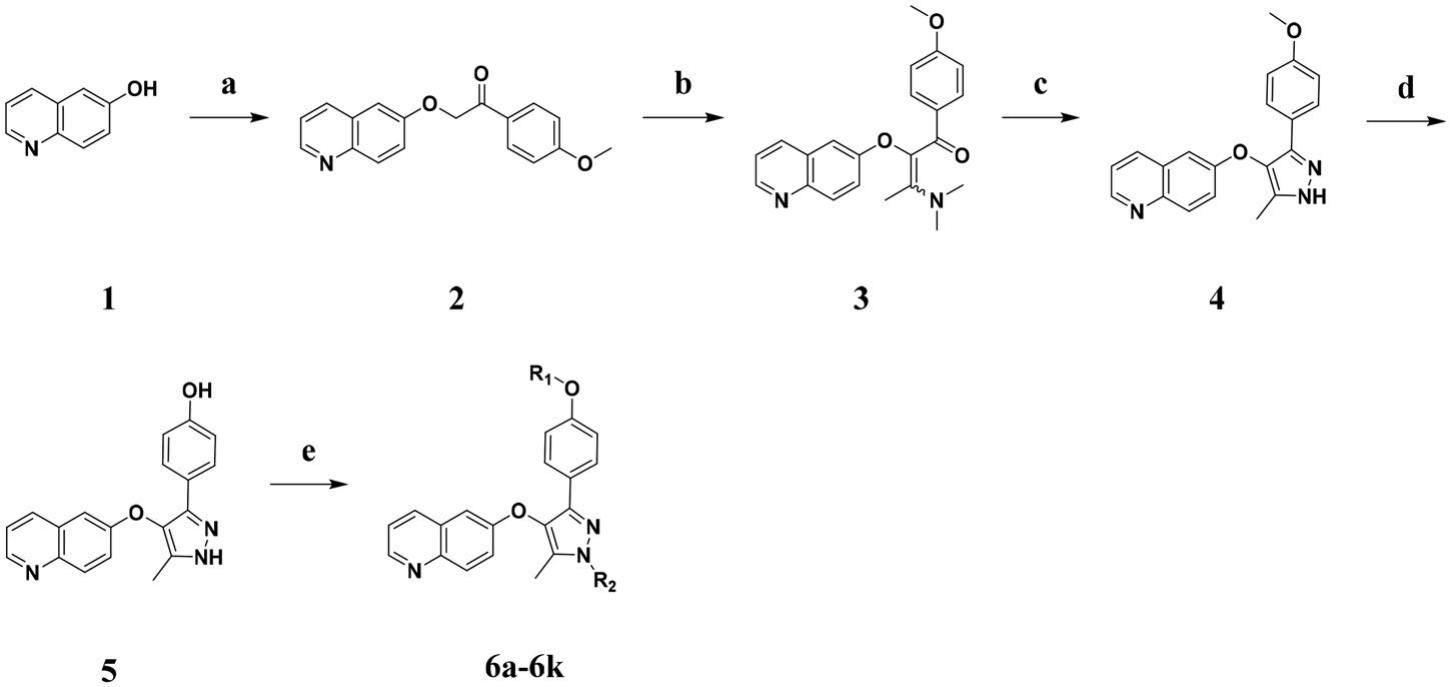
The routes of target compounds. Reagents and conditions: (a) 2-bromo-4′-methoxyacetophenone, DBU, dry DMF, 140 °C; (b) 1,1-dimethoxy-*N*,*N*-dimethylethanamine, 90 °C; (c) hydrazinium hydroxide, ethyl alcohol, 40 °C; (d) BBr_3_, DCM, −78 °C; and (e) methyl bromoacetate or ethyl bromoacetate, K_2_CO_3_, dry DMF, rt.

The synthetic route to compounds **6a–k** is outlined in Scheme 1. Intermediate **4** was synthesised through a three-step sequence involving etherification, condensation, and pyrazole ring formation. Target compounds **6a–k** were prepared by demethylation followed by substitution nucleophilic 2 (SN2) substitution. Detailed experimental specifications for Scheme 1 are provided in Table 1.

**Table 1.** Details of the compounds synthesised in Scheme 1.

Compounds	R_1_	R_2_	Yield	MP (°C)	Log *S*^a^	Log *D*^b^	Log *P*^c^
**4**	CH_3_	H	40%	222.5–224.5	−3.35	3.72	3.29
**5**	H	H	77%	235.7–237.7	−3.64	3.08	2.85
**6a**	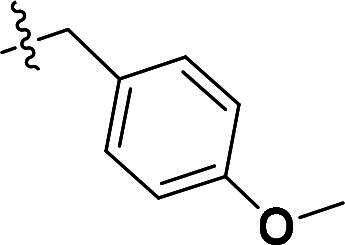	H	71%	157.5–159.5	−4.59	3.99	4.11
**6b**	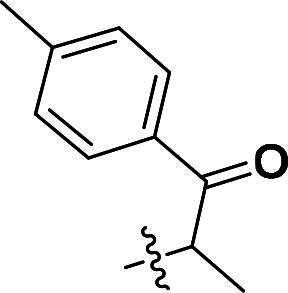	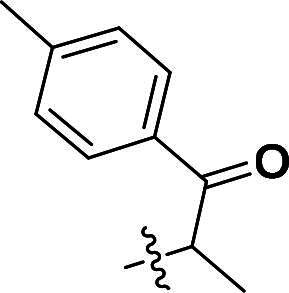	52%	196.6–198.6	−7.41	4.62	5.87
**6c**	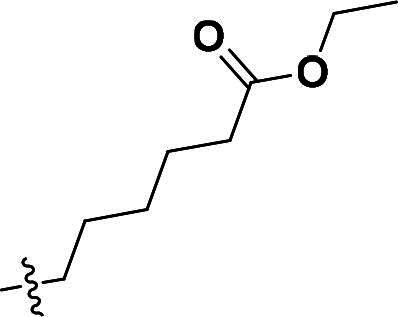	H	90%	181.4–183.4	−5.73	3.82	4.52
**6d**	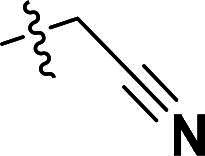	H	82%	183.2–185.2	−4.10	3.06	2.82
**6e**	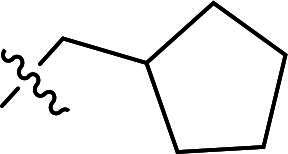	H	87%	186.7–188.7	−5.52	3.90	5.00
**6f**	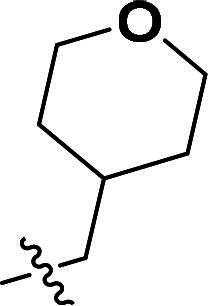	H	90%	177.8–179.8	−5.81	3.77	4.05
**6g**	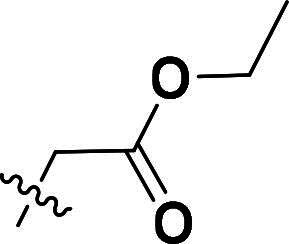	H	73%	155.7–157.7	−4.09	3.37	3.15
**6h**	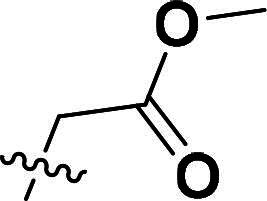	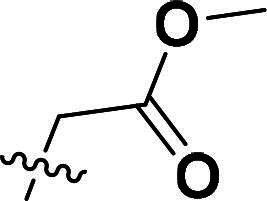	64%	173.5–175.5	−4.38	2.66	2.26
**6i**	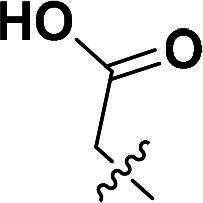	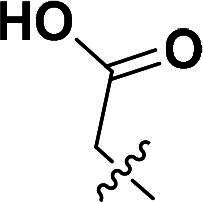	95%	181.8–183.8	−3.13	1.31	1.22
**6j**	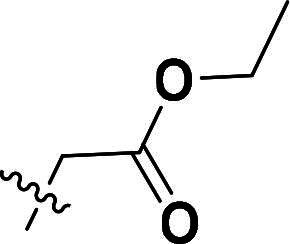	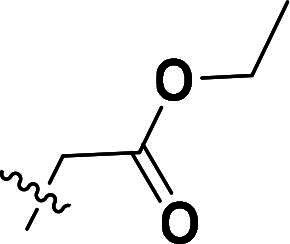	67%	160.6–162.6	−4.40	3.17	3.03
**6k**	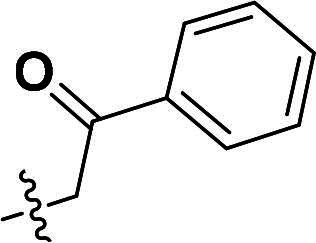	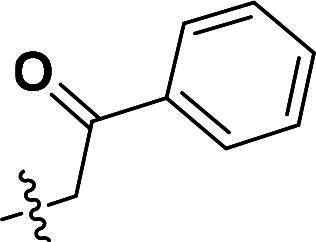	45%	154.3–156.3	−5.81	3.77	4.05

^a^Logarithm of the molar solubility.

^b^Logarithm of the distribution coefficient.

^c^Logarithm of the partition coefficient, the parameters a, b, and c were computationally derived using the ADMETlab platform (https://admetlab3.scbdd.com/), a web-based tool for predicting absorption, distribution, metabolism, excretion, and toxicity (ADMET) properties.

**Scheme 2.**
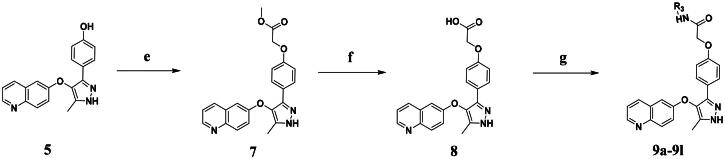
The routes of target compounds. Reagents and conditions: (e) methyl bromoacetate or ethyl bromoacetate, K_2_CO_3_, dry DMF, rt; (f) NaOH aq., 1,4-dioxane, 60 °C; and (g) HATU, DIPEA, DMF, rt.

The synthetic route to compounds **9a–l** is delineated in Scheme 2. Intermediate **7** was synthesised via SN2 of compound **5** with methyl bromoacetate, followed by ester hydrolysis. Target compounds **9a–l** were obtained through amide coupling of **8**. Detailed synthetic parameters for Scheme 2 are provided in Table 2.

**Table 2.** Details of the compounds synthesised in Scheme 2.

Compounds	R_3_	Yield	MP (°C)	Log *S*^a^	Log *D*^b^	Log *P*^c^
**7**	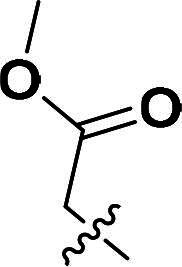	82%	219.5–221.5	−4.34	2.82	2.54
**8**	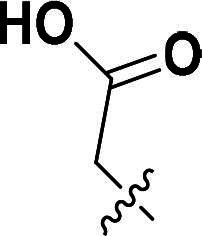	96%	251.3–253.3	−4.17	2.23	2.24
**9a**	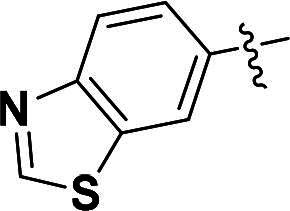	25%	197.4–199.4	−5.06	3.34	3.25
**9b**	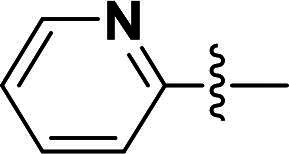	21%	230.9–232.9	−4.38	3.21	3.01
**9c**	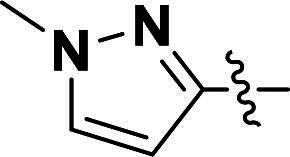	43%	256.9–258.9	−4.15	3.06	2.80
**9d**	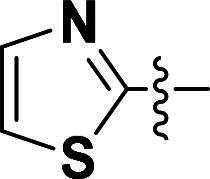	42%	246.5–248.5	−4.90	3.40	3.15
**9e**	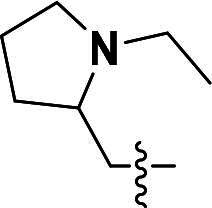	61%	230.3–232.3	−4.08	3.06	3.12
**9f**	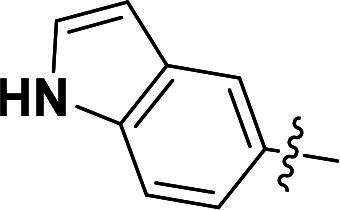	34%	239.7–241.7	−5.01	3.41	3.27
**9g**	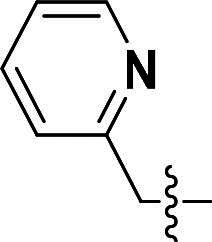	57%	214.2–216.2	−4.05	2.97	2.74
**9h**	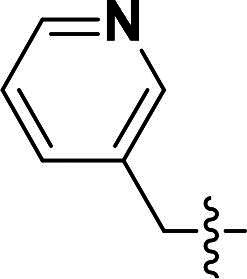	43%	247.7–249.7	−4.12	3.11	2.91
**9i**	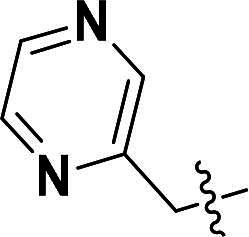	69%	218.2–220.2	−3.55	2.56	2.16
**9j**	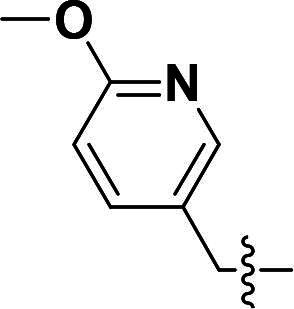	58%	228.8–230.8	−4.83	3.36	3.33
**9k**	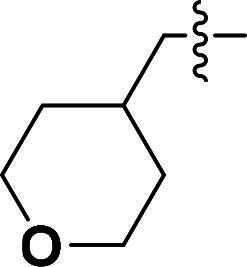	76%	248.1–250.1	−4.29	3.08	2.81
**9l**	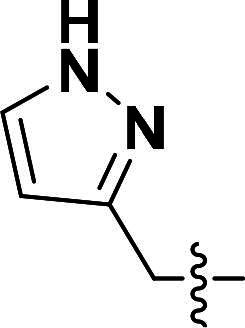	80%	232.3–234.3	−3.72	2.65	2.29

^a^Logarithm of the molar solubility.

^b^Logarithm of the distribution coefficient.

^c^Logarithm of the partition coefficient, the parameters a, b, and c were computationally derived using the ADMETlab platform (https://admetlab3.scbdd.com/), a web-based tool for predicting absorption, distribution, metabolism, excretion, and toxicity (ADMET) properties.

The synthesised compounds exhibit a broad melting point (MP) range (154.3–258.9 °C), reflecting significant structural diversity introduced by the substituents (R_1_, R_2_, R_3_). Notably, compounds with higher MPs often correlate with lower predicted aqueous solubility (Log S, all negative: −3.13 to −7.41). Across the series, the positive partition coefficient (Log P, 1.22–5.87) and distribution coefficient (Log D, 1.31–4.62) values confirm a general trend towards lipophilicity.

### Cytotoxicity assay

Firstly, the cytotoxicity of all the synthesised compounds was assessed using the mouse microglial (BV2) cells. As shown in Table 3, most compounds did not exhibit cytotoxicity at 10 μM, except for compounds **6a**, **6b**, **6d**, **9a**, **9b**, **9f**, and **9i** with toxicity.

**Table 3.** Cell viability of BV2 cells treated with different compounds, Dex and Cel for 24 h (*n* = 3).

Compound	CCK-8 (%)	Compound	CCK-8 (%)
Control	99.87 ± 0.48	**7**	101.61 ± 3.2
Dex	79.49 ± 2.67	Cel	77.59 ± 0.43
**4**	97.82 ± 12.09	**8**	130.07 ± 3.21
**5**	92.78 ± 3.5	**9a**	88.73 ± 1.57
**6a**	34.52 ± 7.99	**9b**	86.4 ± 1.03
**6b**	72.09 ± 17.82	**9c**	106.21 ± 1.28
**6c**	75.56 ± 18.89	**9d**	138.35 ± 10.48
**6d**	40.55 ± 24.92	**9e**	105.38 ± 1.68
**6e**	90.8 ± 13.62	**9f**	67.67 ± 1.99
**6f**	95.52 ± 27.57	**9g**	100.13 ± 1.88
**6g**	100.5 ± 0.4	**9h**	106.64 ± 3.34
**6h**	98.1 ± 1.07	**9i**	40.55 ± 24.92
**6i**	102.31 ± 0.32	**9j**	90.8 ± 13.62
**6j**	112.93 ± 7.55	**9k**	95.52 ± 27.57
**6k**	104.79 ± 5.22	**9l**	100 ± 17.23

Data are represented as mean ± SD.

### In vitro anti-inflammatory activity and structure–activity relationships study

**Figure 3.**
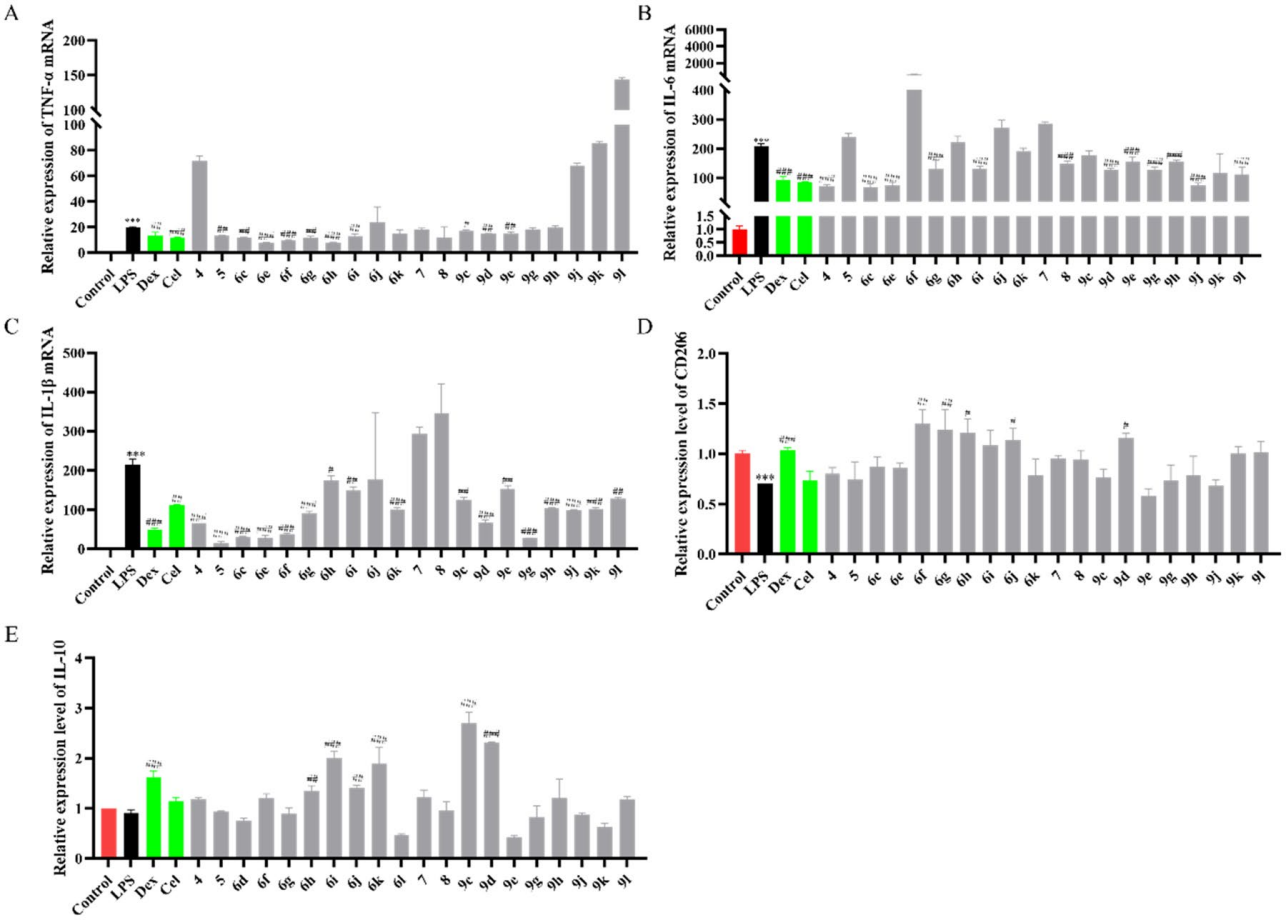
Anti-inflammatory activity of compounds in BV2 cells. qRT-PCR analysis of TNF-α (A), IL-6 (B) and IL-1β (C), CD206 (D) and interleukin-10 (IL-10) (E) mRNAs in the BV2 cells treated with different compounds for 2 h after being stimulated by LPS for 4 h (*n* = 3). Data are represented as mean ± SD. Compared with Control group, ***P < 0.001. Compared with LPS group, ^#^*P* < 0.05, ^##^*P* < 0.01, ^###^*P* < 0.001.

In order to explore the anti-inflammatory ability of compounds, inflammatory cytokine-related indicators were detected. The expression of pro-inflammatory factors (Figure 3(A–C) and Tables S1–S3) was inhibited, and the expression of anti-inflammatory factors (Figure 3(D–E) and Tables S4 and S5) was increased by most compounds on lipopolysaccharide (LPS) stimulated BV2 cells. The preliminary results showed that two compounds (**6g, 9g**) exhibited good effects in BV2 cells, and compound **6g** exhibited the strongest inhibitory effect.

**Figure 4.**
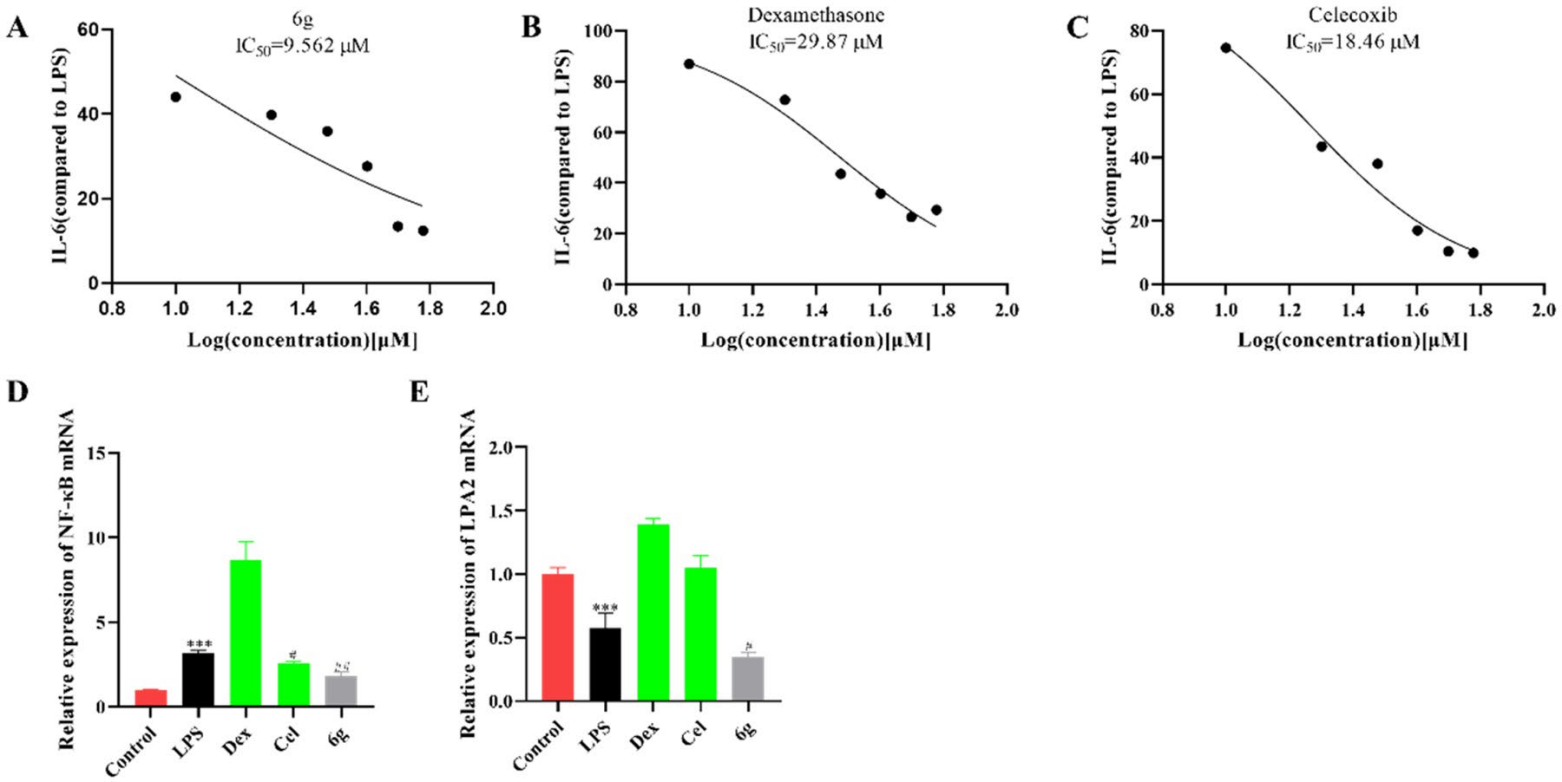
Inhibition of **6g** (A), Dex (B) and Cel (C) on IL-6 secretion in the LPS-stimulated BV2 cells (*n* = 3). qRT-PCR analysis of NF-κB (D) and LPA2 (E) mRNAs in the BV2 cells (*n* = 3). Data are represented as mean ± SD. Compared with Control group, ***P < 0.001. Compared with LPS group, ^#^*P* < 0.05, ^##^*P* < 0.01, ^###^*P* < 0.001.

Based on the above results, **6g** was selected to investigate the potential dose-dependent inhibition of cytokines. The IL-6 expression induced by LPS stimulation in BV2 cells was inhibited by compound **6g** (Figure 4(A)), and the IC_50_ was 9.562 μM. The IC_50_ of dexamethasone (Dex) was 29.87 μM (Figure 4(B)), and the IC_50_ of Cel was 18.46 μM (Figure 4(C)). The results were indicated that compound **6g** showed great anti-inflammatory activity.

The nuclear factor kappa-light-chain-enhancer of activated B cells (NF-κB) pathway has been reported to be one of the main pathways inducing inflammation,^34–36^ in order to explore the mechanism by which compound **6g** regulates inflammation, the expression of NF-κB was detected by quantitative real-time PCR (qRT-PCR). The expression of NF-κB was increased after being stimulated by LPS in BV2 cells (Figure 4(D) and Table S6). However, it was reversed by compound **6g**. The expression of LPA2 was detected by qRT-PCR. As shown in Figure 4(E) and Table S7, the expression of LPA2 was decreased by compound **6g** in LPS-BV2 cells.

**Figure 5.**
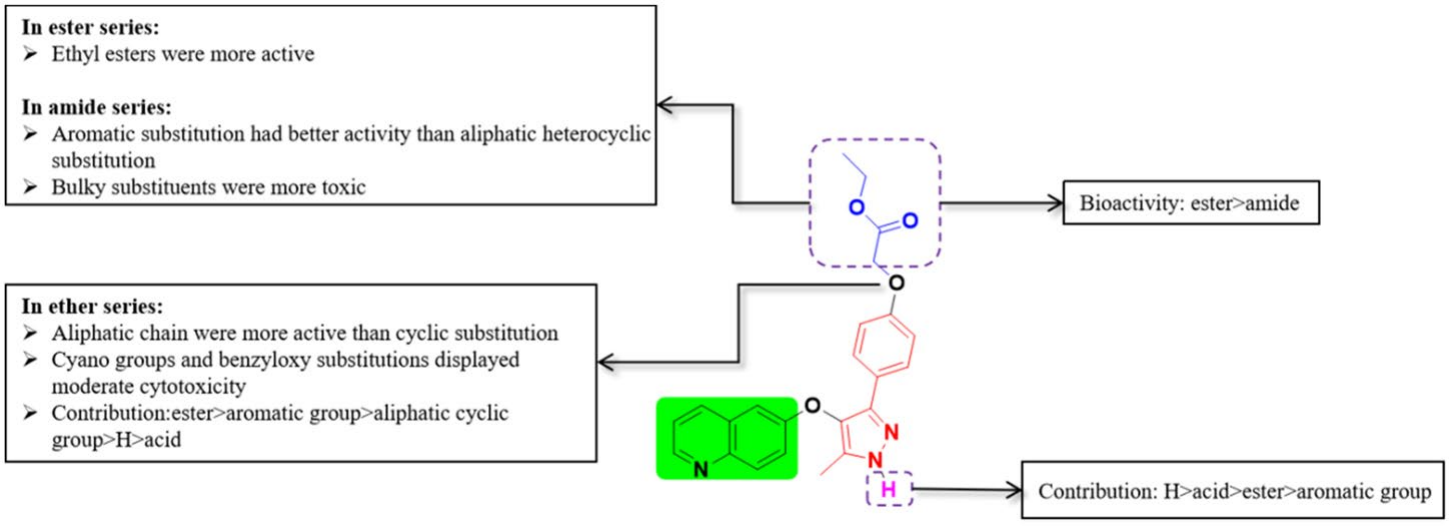
Brief Structure Activity Relationship analysis of this work.

Based on the results of the anti-inflammatory screening experiments, we outlined the structure–activity relationships. Compound **6g** exhibited excellent anti-inflammatory activity, indicating that the contribution of ester substitution was more significant than that of amide substitution. In the ester series, ethyl esters exhibited enhanced activity relative to methyl esters, with longer alkyl chains generally showing better performance than shorter ones. Within the amide series, aromatic substitutions demonstrated significantly enhanced biological activity relative to their heterocyclic counterparts. Notably, bulky substituents exhibited a proportional increase in toxicity profiles, with steric bulk showing positive correlation with cytotoxic potency. Among the entire ether series, aliphatic chain substitutions demonstrated superior activity compared to cyclic substitutions. Notably, cyan groups and benzyloxy substitutions displayed moderate cytotoxicity. The overall contribution of substituents was established as follows: aliphatic chain substitutions provided the most significant activity enhancement, followed by aromatic substitutions, which surpassed both unsubstituted (H) groups and aliphatic cyclic functionalities. Carboxylic acid derivatives showed the lowest activity levels among the tested substituents. For pyrazole substitution, the order of activity was H > acid > ester > aromatic (Figure 5).

### Regulation of the expression of CD68 and CD206 in BV2 cells

**Figure 6.**
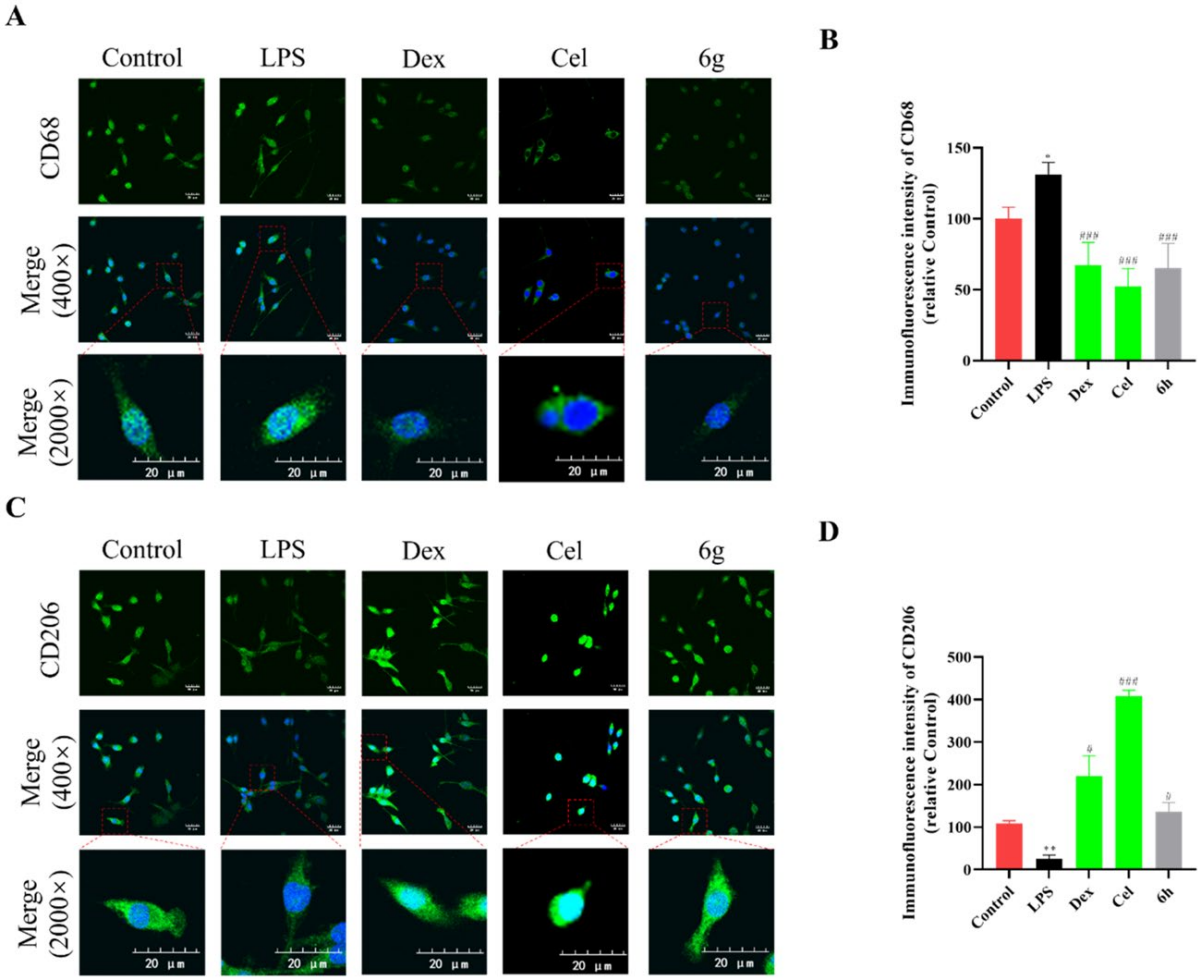
The expression of CD68 and CD206 in BV2 cells stimulated by LPS. (A,B) Immunofluorescence staining analysis of CD206 expression in the BV2 cells treated with LPS for 4 h (*n* = 3). (C,D) Immunofluorescence staining analysis of CD68 expression in the BV2 cells treated with LPS for 4 h (*n* = 3). Data are represented as mean ± SD. Compared with Control group, **P < 0.01, ***P < 0.001. Compared with LPS group, ^#^*P* < 0.05, ^###^*P* < 0.001.

Studies have shown that after activating microglia, it differentiates into MI and MII phenotypes.^37^ MI microglia secretes pro-inflammatory factors, and overactivated MI microglia has obvious neurotoxic effects.^37^ Anti-inflammatory cytokines are secreted by MII-type microglia, which plays an important role in promoting inflammation repair.^37^ LPS-induced microglial activation is characterised by a large increase of cells with the MI phenotype.^38^ To investigate whether anti-inflammatory cytokines could be activated by compound **6g**, the expression of macrophage mannose receptor 1 (CD206) and cluster of differentiation 68 (CD68) was detected. As shown in Figure 6(A,B), the expression of CD68 was significantly decreased in BV2 cells after pre-treatment with compound **6g** (10 μM) in LPS-induced BV2 cells. As shown in Figure 6(C,D), Control with LPS group, the expression of CD206 was significantly increased in BV2 cells after pre-treatment with compound **6g** (10 μM).

### Regulation of the expression of oxidative stress in BV2 cells

**Figure 7.**
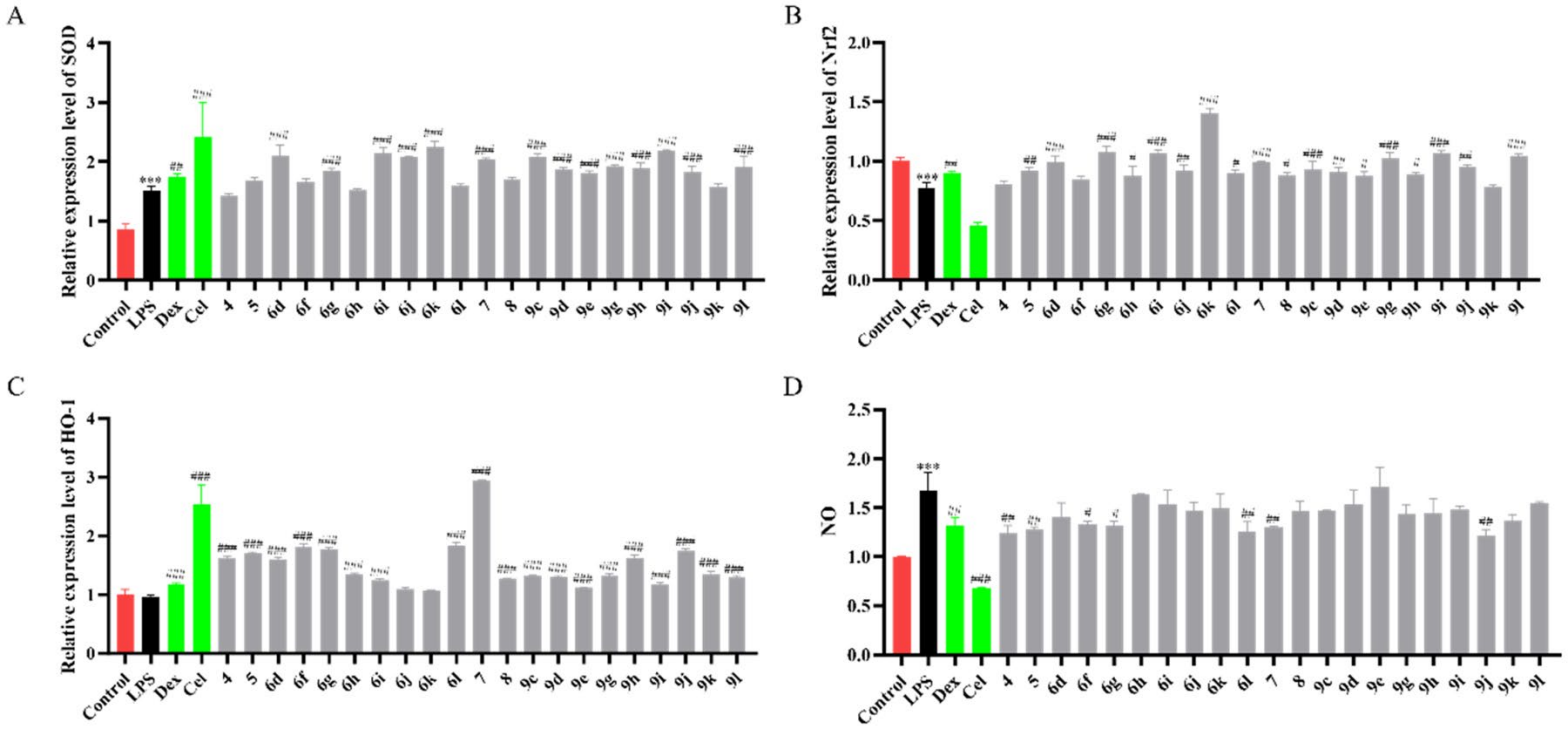
Antioxidant activity of compounds in BV2 cells. qRT-PCR analysis of superoxide dismutase (SOD) (A), Nrf2 (B), HO-1 (C), and NO (D) mRNAs in the BV2 cells treated with different compounds for 2 h after being stimulated by LPS for 4 h (*n* = 3). Data are represented as mean ± SD. Compared with Control group, ***P < 0.001. Compared with LPS group, ^#^*P* < 0.05, ^##^*P* < 0.01, ^###^*P* < 0.001.

Neuroinflammation generally occurs with oxidative stress in cells.^39^ Thus, the antioxidant capacity of compounds was tested by qRT-PCR. The results were shown in Figure 7(A–D) and Tables S8–S11, compared with Control group, the mRNA levels of nuclear factor erythroid 2-related factor 2 (Nrf2) and haem oxygenase-1 (HO-1) were promoted by compound **6g** in LPS-induced BV2 cells, showing good antioxidant capacity.

**Figure 8.**
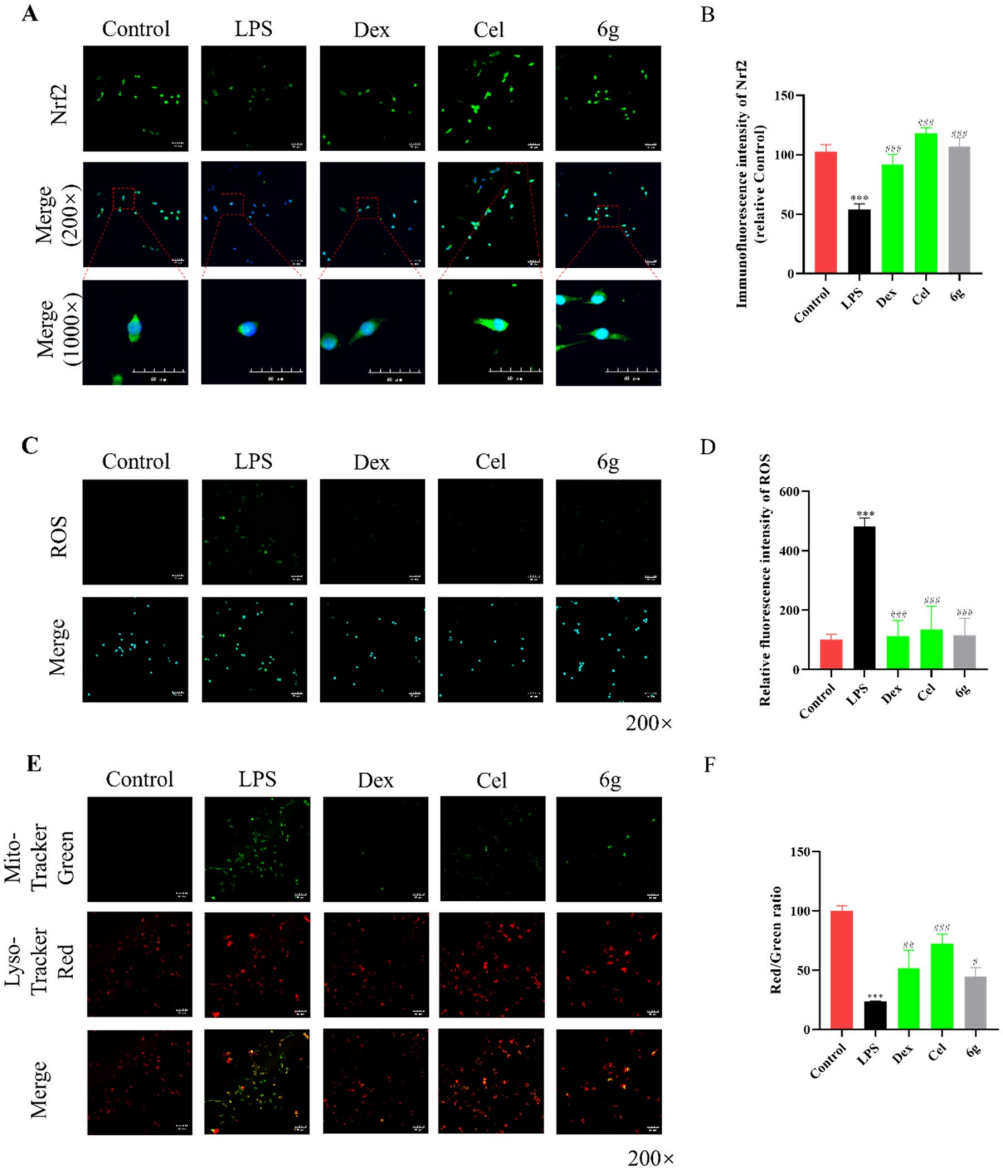
The expression of oxidative stress in BV2 cells stimulated by LPS. (A,B) Immunofluorescence staining analysis of Nrf2 expression in the BV2 cells treated with LPS for 4 h (*n* = 3). (C,D) ROS staining analysis in the BV2 cells treated with LPS for 4 h (*n* = 3). (E,F) Detection of mitochondrial membrane potential in the BV2 cells treated with LPS for 4 h (*n* = 3). Data are represented as mean ± SD. Compared with Control group, ***P < 0.001. Compared with LPS group, ^###^*P* < 0.001.

Immunofluorescence result showed that Nrf2 expression was down-regulated in the LPS group compared with the Control group (Figure 8(A,B)). However, **6g** administration could promote the protein expression of Nrf2. ROS levels are elevated in inflammation beyond the normal physiological range, and then mitochondrial membrane potential is affected,^40^ so mitochondrial membrane potential and the expression of ROS were detected *in vitro*. As shown in the Figure 8(C–F), treatment with different concentrations of **6g** was found to inhibit ROS expression, enhance mitochondrial membrane potential and thereby attenuate LPS-induced cell damage **(**10 μM).

### Molecular docking and molecular dynamics simulations

**Figure 9.**
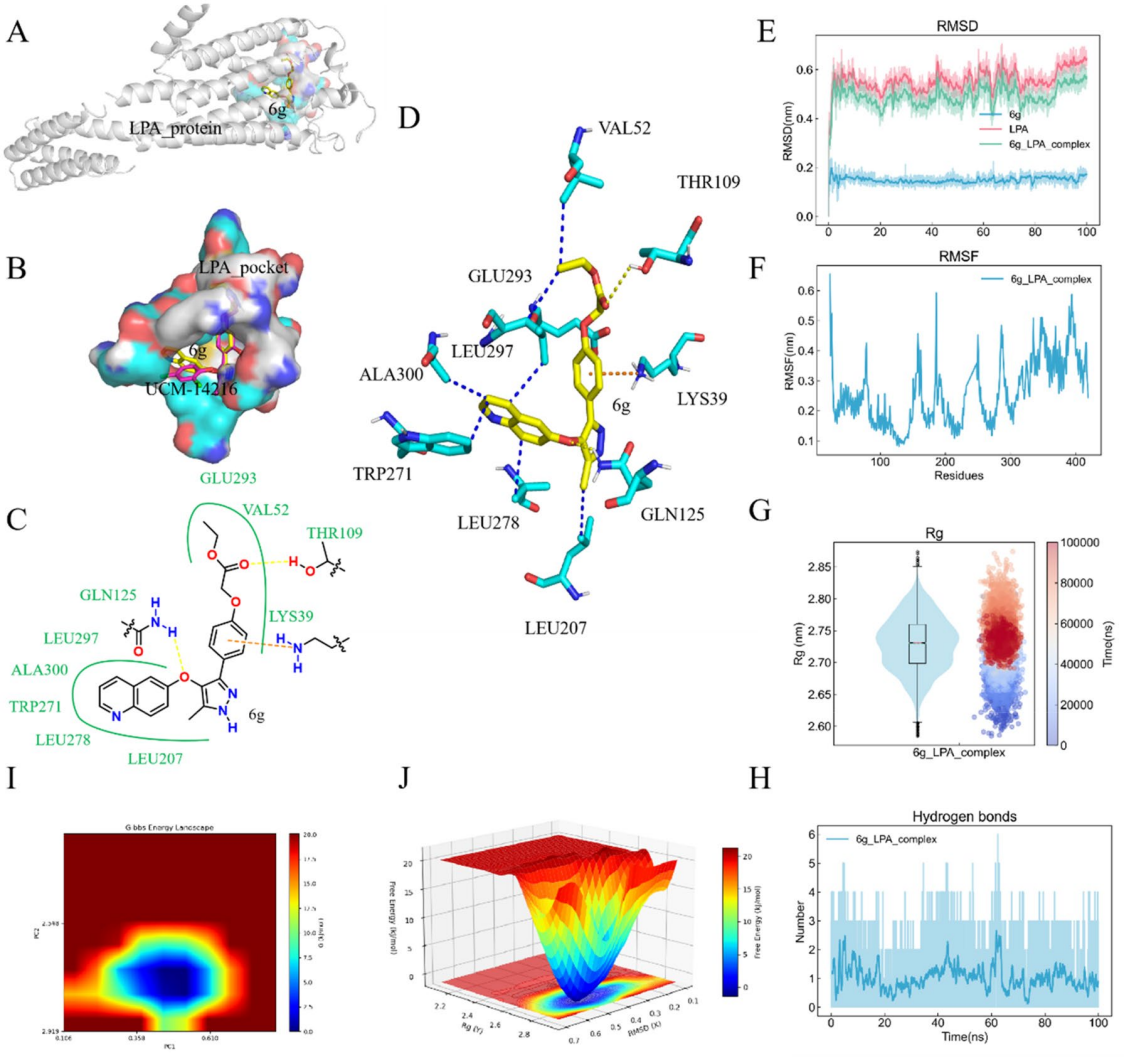
The docking and MD simulation of compound **6g** and LPA (PDB: 4Z35). Schematic representation of the binding site of compound **6g** with 4Z35 (A). Schematic representation of the active pocket of compound **6g**, UCM-14216, bound to the 4Z35 protein (B). 2D Interaction forces between compound **6g** and LPA (C). 3D Interaction forces between compound **6g** and LPA (D, yellow dashed lines: hydrogen bonds, blue dashed lines: hydrophobic interactions, orange dashed line: π-cation). RMSD is calculated for LPA protein, LPA protein–**6g** ligand complexes and compound **6g** (E). RMSF analysis of LPA protein–**6g** ligand complexes (F). RG analysis of LPA protein–**6g** ligand complexes (G). H-bonding interactions of LPA protein–**6g** ligand complexes (H). Gibbs free-energy topography of LPA protein–**6g** ligand complexes in 2D (I). Gibbs free-energy topography of LPA protein–**6g** ligand complexes in 3D (J).

To further investigate the interaction between compound **6g** and LPA, we performed computerised molecular docking simulations using AutoDock Vina. The output (PDB: 4Z35, Figure 9(A)) was then visualised, revealing that compound **6g** formed hydrogen bonding interactions with amino acids THR109 and GLN125, and a π-cation interaction with amino acid LYS39. To verify the docking results, compound **6g** was compared with the reported compound UCM-14216 (Figure 9(B)). It was found that both compounds were in the same protein pocket and had extremely similar conformations with almost complete overlap. Furthermore, the ether group substitution region of compound **6g** is located in the hydrophobic pocket formed by GLU293, VAL52, THR109 and LYS39, while the quinoline ring is located in the hydrophobic pocket formed by GLN125, LEU207, LEU278, TRP271, ALA300 and LEU297. These regions bind to the corresponding amino acids through hydrophobic interactions (see Figure 9(C,D)), and the lowest calculated binding energy was −9.33 kcal/mol. This indicates that compound **6g** has a strong affinity for LPA.

To verify the stability of docking in more depth, we performed simulation experiments on the complexes to investigate how changes in protein flexibility and structural alterations affect the interaction characteristics of the complexes, using molecular dynamics simulations. During the simulation, the protein’s root mean square deviation (RMSD) increased initially and then changed steadily, indicating that the system had stabilised. We also calculated the RMSD value of the protein–ligand complex (Figure 9(E) for details of the RMSD curves), and the smooth RMSD value intuitively indicates the complex’s conformational stability. The flexibility of the protein atoms was measured using root-mean-square free vibration (RMSF). This parameter is a key factor in determining the stability of protein–ligand complexes, and the RMSF values of the complexes are displayed in Figure 9(F). Overall, the simulation results show that the RMSF values of the complexes are lower and do not exhibit significant fluctuations, consistent with the characteristics of the RMSD curves, which remain within a reasonable fluctuation range.

The radius of gyration (RG) is another important parameter for assessing the degree of densification of protein–ligand complexes.^41^ This parameter reflects the structural compactness of the protein–ligand interaction; lower values indicate less unfolding of the complex (a more compact structure). As shown in Figure 9(G), most of the RG values of the complex are between 2.7 and 2.75 nm in the violin plots. From the scatter plots, it can be seen that the RG values of the complexes first increased, then decreased and finally stabilised at around 2.73 nm over time. This demonstrates that the complexes have a certain degree of compactness during the simulation process. In summary, it indicates that the free state protein and the selected substances can effectively maintain the stability and dense structure of the complex.

The stability of proteins can be significantly affected by the number of hydrogen bonds formed with different substances. Figure 9(H) shows the average number of hydrogen bonds formed by complexes containing proteins and ligands. Throughout the simulation, compound **6g** formed two stable hydrogen bonds with the protein, whereas the other hydrogen bonds formed were unstable.

The Free Energy Landscape (FEL) is a graphical tool that visualises the free-energy changes of a molecular system across various conformations or states. By using the RG and RMSD as coordinates and applying statistical methods to calculate the associated free-energy distributions, insights into the stability and dynamics of molecules in different conformations and states can be gained. As shown in the energy diagrams of Figure 9(I,J), the blue region corresponds to the energy regime of the system with stable conformations. In this region, the system exhibits the lowest free-energy values for these conformations, underscoring the high stability of the protein structure in such states. Analysing this blue region enables the identification of the most stable protein conformations, as well as potential folding pathways and key intermediate states. This understanding is crucial for elucidating the functional and kinetic characteristics of proteins.

For the molecular docking and molecular dynamics simulations described above, AutoDock Vina 4.2.6 and Gromacs 2023.2 were employed, respectively.^42–44^ In the molecular docking process, the box coordinates were set as centre *x* = −3.0, centre *y* = −27.2, centre *z* = 51.7, with dimensions size *x* = 21.5, size *y* = 19.6, and size *z* = 20.7. Additionally, the parameters exhaustiveness = 16 and number modes = 20 were utilised. For the molecular dynamics simulation, the CHARMM36 force field was selected, and the TIP3P water model was adopted, a dodecahedral box was defined.

## Conclusions

In summary, our study identifies the novel pyrazole derivative **6g** as a potent antagonist of the LPA2 receptor, which plays a detrimental role in neuroinflammation after SCI. Compound **6g** exhibits dual anti-inflammatory and antioxidant properties by significantly suppressing pro-inflammatory cytokine release, reducing ROS, and activating the Nrf2/HO-1 pathway. Supported by molecular docking that confirmed its strong binding affinity for LPA2, these findings highlight the compound’s significant protective effects in cellular models and its considerable therapeutic potential for the treatment of SCI.

## Materials and methods

### Solvents and chemicals

Reagents and solvents were commercially available and purchased from Energy Chemical and Bide Pharm. Both ^1^H NMR and ^13^C NMR were recorded on a Bruker AVIII-400 spectrometer. Abbreviations for signal coupling are as follows: s, singlet; d, doublet; t, triplet; m, multiplet and br, broad. protons for NH and OH were not observed and they have merged with the base line of the spectra. High resolution mass spectra were measured using AB Sciex Triple TOF^™^ 5600 plus. The purity of the final compounds was >94%, as analysed by HPLC (SHIMADZU LC-20 A, UV detection at 254 nm) on a C18 column (4.6 mm × 150 mm, 5 μm).

### Synthesis of target compounds

#### Synthesis of compound 2

To a solution of 6-hydroxyquinoline (1050 mg, 7.28 mmol, 1 equiv.) in anhydrous *N*,*N*-dimethylformamide (DMF), 2-bromo-4′-methoxyacetophenone (2000 mg, 8.73 mmol, 1.2 equiv.) and 1,8-diazabicyclo[5.4.0]-undec-7-ene (DBU) (1330 mg, 8.73 mmol, 1.2 equiv.) were added. The reaction mixture was heated at 140 °C for 30 min. Upon completion, the mixture was cooled to room temperature, and diluted with ethyl acetate. The organic layer was washed with water and brine, dried over sodium sulphate (Na_2_SO_4_), filtered, and concentrated under reduced pressure. The residue was purified by silica gel column chromatography to obtain intermediate **2** as brownish yellow oil. Yield: 90%.

***1-(4-Methoxyphenyl)-2-(quinolin-6-yloxy)ethan-1-one (2)***. ^1^H NMR (400 MHz, DMSO-*d*_6_) *δ* 8.74 (dd, *J* = 4.0, 1.6 Hz, 1H, Quinoline-CH), 8.19 (dd, *J* = 8.0, 1.6 Hz, 1H, Quinoline-CH), 8.09–8.02 (m, 2H, Methoxy-Ph-CH), 7.96 (d, *J* = 9.2 Hz, 1H, Quinoline-CH), 7.51 (dd, *J* = 9.2, 2.8 Hz, 1H, Quinoline-CH), 7.45 (dd, *J* = 8.4, 4.0 Hz, 1H, Quinoline-CH), 7.39 (d, *J* = 2.8 Hz, 1H, Quinoline-CH), 7.14–7.08 (m, 2H, Methoxy-Ph-CH), 5.68 (s, 2H, CH_2_), 3.86 (s, 3H, Methoxy-CH_3_). ^13^C NMR (101 MHz, DMSO-d6) *δ* 192.9, 164.1, 156.4, 148.6, 144.4 (2 × C), 135.2, 130.9, 130.7 (2 × C), 129.3, 127.8, 122.4, 122.1, 114.5, 107.6, 70.6, 56.1. Chemical formula: C_18_H_15_O_3_N.

#### Synthesis of compound 3

Compound **2** (263 mg, 0.85 mmol, 1 equiv.) and 1,1-dimethoxy-*N*,*N*-dimethylethanamine (1.0 ml, 7.14 mmol, 8.4 equiv.) were stirred at 90 °C for 5 h. Upon completion of the reaction, the mixture was cooled to room temperature, diluted with ethyl acetate, and washed with water and brine. The organic layer was dried over Na_2_SO_4_, filtered, and concentrated under reduced pressure. The residue was purified by silica gel column chromatography to obtain intermediate **3** as brownish yellow oil. Yield: 45%.

***3-(Dimethylamino)-1-(4-methoxyphenyl)-2-(quinolin-6-yloxy)but-2-en-1-one (3)***. Mixture of conformer A:B (6:1). Major conformer A: ^1^H NMR (500 MHz, CDCl_3_) *δ* 8.71 (dd, *J* = 4.0, 1.5 Hz, 1H, Quinoline-CH), 7.90 (d, *J* = 6.0 Hz, 1H, Quinoline-CH), 7.41–7.38 (m, 2H, Methoxy-Ph-CH), 7.31–7.29 (m, 1H, Quinoline-CH), 7.28–7.25 (m, 1H, Quinoline-CH), 6.86 (d, *J* = 2.5 Hz, 1H, Quinoline-CH), 6.80–6.77 (m, 2H, Methoxy-Ph-CH), 6.41 (d, *J* = 2.0 Hz, 1H, Quinoline-CH), 3.74 (s, 3H, Methoxy-CH_3_), 3.04 (s, 6H, N-CH_6_), 2.76 (s, 3H, vinyl-CH_3_). Minor conformer B: ^1^H NMR (500 MHz, CDCl3) *δ* 8.74 (dd, *J* = 4.0, 1.5 Hz, 1H, Quinoline-CH), 7.93 (t, *J* = 8.5 Hz, 1H, Quinoline-CH), 7.44–7.41 (m, 2H, Methoxy-Ph-CH), 7.34–7.32 (m, 1H, Quinoline-CH), 7.31–7.28 (m, 1H, Quinoline-CH), 6.89 (d, *J* = 2.5 Hz, 1H, Quinoline-CH), 6.83–6.80 (m, 2H, Methoxy-Ph-CH), 6.43 (d, *J* = 2.0 Hz, 1H, Quinoline-CH),3.80 (s, 3H, Methoxy-CH_3_), 3.05 (s, 6H, N–CH_6_), 2.78 (s, 3H, vinyl-CH_3_). Chemical formula: C_22_H_22_O_3_N_2_.

#### Synthesis of compound 4

Hydrazinium hydroxide (65%, 0.18 ml/mmol) was added to a solution of compound **3** (154 mg, 0.41 mmol) in anhydrous ethanol. The reaction mixture was heated to reflux for two hours, then cooled to room temperature, and concentrated under reduced pressure. The residue was dissolved in a small amount of ethyl acetate, acidified with hydrochloric acid (HCl), and extracted with ethyl acetate. The combined organic layers were washed with water and brine, dried over Na_2_SO_4_, filtered, and concentrated under reduced pressure. The residue was purified by silica gel column chromatography to obtain intermediate **4** as a white solid. Yield: 40%.

***6-((3-(4-Methoxyphenyl)-5-methyl-1H-pyrazol-4-yl)oxy)quinoline (4)***. ^1^H NMR (500 MHz, CDCl_3_) *δ* 8.78 (dd, *J* = 4.0, 2.0 Hz, 1H, Quinoline-CH), 8.08 (d, *J* = 9.0 Hz, 1H, Quinoline-CH_),_ 7.93 (dd, *J* = 8.5, 2.0 Hz, 1H, Quinoline-CH), 7.64 (d, *J* = 9.0 Hz, 2H, Methoxy-Ph-CH), 7.56 (dd, *J* = 9.0, 3.0 Hz, 1H, Quinoline-CH), 7.32 (dd, *J* = 8.5, 4.0 Hz, 1H, Quinoline-CH), 7.06 (d, *J* = 3.0 Hz, 1H, Quinoline-CH), 6.84 (d, *J* = 9.0 Hz, 2H, Methoxy-Ph-CH), 3.75 (s, 3H, Methoxy-CH_3_), 2.17 (s, 3H, Pyrazole-CH_3_). HPLC: *t*_R_ 10 min, purity 97.88%. Chemical formula: C_20_H_17_O_2_N_3_.

#### Synthesis of compound 5

Boron tribromide (453 mg, 1.8 mmol, 3 equiv.) was added to a solution of compound **4** (200 mg, 0.6 mmol, 1 equiv.) in anhydrous dichloromethane (DCM, 6.0 ml) at −78 °C. The mixture was kept at −78 °C for 1 h. The reaction was then warmed to room temperature and stirred for an additional 20 h. Upon completion, the reaction mixture was diluted with ethyl acetate and washed with water and brine. The organic layer was dried over Na_2_SO_4_, filtered, and concentrated under reduced pressure. The residue was purified by silica gel column chromatography to obtain intermediate **5** as a yellow solid. Yield: 77%.

***4-(5-Methyl-4-(quinolin-6-yloxy)-1H-pyrazol-3-yl)phenol (5)***. ^1^H NMR (600 MHz, DMSO-*d*_6_) *δ* 9.13 (dd, *J* = 4.8, 1.2 Hz, 1H, Quinoline-CH), 8.93 (d, *J* = 8.4 Hz, 1H, Quinoline-CH), 8.28 (d, *J* = 9.0 Hz, 1H, Quinoline-CH), 7.96 (dd, *J* = 9.0, 2.4 Hz, 1H, Quinoline-CH), 7.93 (dd, *J* = 8.4, 4.8 Hz, 1H, Quinoline-CH), 7.53 (d, *J* = 3.0 Hz, 1H, Quinoline-CH), 7.51 (d, *J* = 8.4 Hz, 2H, Methoxy-Ph-CH), 6.73 (d, *J* = 8.4 Hz, 2H, Methoxy-Ph-CH), 2.07 (s, 3H, Pyrazole-CH_3_). ^13^C NMR (151 MHz, DMSO-*d*_6_) *δ* 158.1, 157.6, 144.9, 143.7, 137.2, 136.6, 135.6, 131.8, 130.4, 127.1 (2 × C), 125.6, 125.4, 122.9, 121.5, 116.0 (2 × C), 109.6, 9.9. HPLC: *t*_R_ 10 min, purity 99.58%. Chemical formula: C_19_H_15_O_2_N_3_.

#### Synthesis of compound 6a–6k

To a solution of intermediate 5 (200 mg, 0.63 mmol, 1 equiv.) in anhydrous DMF, K_2_CO_3_ (174 mg, 1.26 mmol, 2 equiv.) was added bromine reagent (96 mg, 0.63 mmol, 1 equiv.). The reaction mixture was stirred at room temperature overnight. Upon completion, the reaction mixture was diluted with ethyl acetate, and the organic layer was washed with water and brine. The organic layer was dried over Na_2_SO_4_, filtered, and concentrated under reduced pressure. The residue was purified by silica gel column chromatography to obtain intermediate **6a–6k**.

***6-((3-(4-((4-Methoxybenzyl)oxy)phenyl)-5-methyl-1H-pyrazol-4-yl)oxy)quinoline (6a)***. ^1^H NMR (400 MHz, DMSO-*d*_6_) *δ* 8.76 (s, 1H, Quinoline-CH), 8.21 (s, 1H, Quinoline-CH), 8.02 (d, *J* = 8.8 Hz, 1H, Quinoline-CH), 7.70–7.56 (m, 3H, Ether-Ph-CH, Quinoline-CH), 7.44 (dd, *J* = 8.4, 4.0 Hz, 1H, Quinoline-CH), 7.32 (d, *J* = 8.0 Hz, 2H, Methoxy-Ph-CH), 7.19 (s, 1H, Quinoline-CH), 7.05–6.85 (m, 4H, Ether-Ph-CH, Methoxy-Ph-CH), 4.95 (s, 2H, CH_2_), 3.72 (s, 3H, Methoxy-CH_3_), 2.06 (s, 3H, Pyrazole-CH_3_). ^13^C NMR (101 MHz, DMSO-*d*_6_) *δ* 159.4, 156.4, 149.2, 144.7, 141.0, 137.2, 135.6, 135.5, 132.5, 131.6, 130.0 (2 × C), 129.3, 126.9 (2 × C), 125.5, 124.5, 122.3, 121.4, 115.3 (2 × C), 114.2 (2 × C), 108.8, 69.4, 55.5, 8.7. ESI-HRMS *m/z*: calcd for C_27_H_24_O_3_N_3_^+^ [M + H]^+^, 438.1812; found, 438.1817. HPLC: *t*_R_ 7 min, purity 98.71%.

***2-(4-(5-Methyl-1-(1-oxo-1-(p-tolyl)propan-2-yl)-4-(quinolin-6-yloxy)-1H-pyrazol-3-yl)phenoxy)-1-(p-tolyl)propan-1-one (6b)***. ^1^H NMR (400 MHz, DMSO-*d*_6_) *δ* 8.77 (dd, *J* = 4.0, 1.6 Hz, 1H, Quinoline-CH), 8.05 (d, *J* = 10.4 Hz, 1H, Quinoline-CH), 7.98 (d, *J* = 9.2 Hz, 1H, Quinoline-CH), 7.90 (d, *J* = 8.4 Hz, 2H, Carbonyl-Ph-CH), 7.78 (d, *J* = 8.0 Hz, 2H, Carbonyl-Ph-CH), 7.54–7.44 (m, 4H, Ether-Ph-CH, Carbonyl-Ph-CH), 7.33 (dd, *J* = 10.0, 8.0 Hz, 4H, Quinoline-CH, Carbonyl-Ph-CH), 6.97 (dd, *J* = 2.8, 1.2 Hz, 1H, Quinoline-CH), 6.77 (d, *J* = 9.2 Hz, 2H, Ether-Ph-CH), 6.20 (q, *J* = 6.8 Hz, 1H, CH), 5.88 (q, *J* = 6.8 Hz, 1H, CH), 2.36 (d, *J* = 9.6 Hz, 6H, CH_3_), 2.12 (d, *J* = 2.4 Hz, 3H, Pyrazole-CH_3_), 1.72 (d, *J* = 6.8 Hz, 3H, CH_3_), 1.45 (d, *J* = 6.8 Hz, 3H, CH_3_). ^13^C NMR (101 MHz, DMSO-*d*_6_) *δ* 197.9, 196.1, 157.1, 156.3, 149.3, 144.9, 144.7, 144.1, 140.4, 137.2, 135.4, 135.3, 133.2, 133.0, 132.0, 131.9, 131.7, 130.0 (2 × C), 129.8 (2 × C), 129.0 (2 × C), 128.6 (2 × C), 127.1 (2 × C), 124.9, 124.5, 122.4, 121.0, 115.4 (2 × C), 108.8, 74.6, 21.7, 18.7, 16.9, 8.8. ESI-HRMS *m/z*: calcd for C_39_H_36_O_4_N_3_^+^ [M + H]^+^, 610.2700; found, 610.2719. HPLC: *t*_R_ 10 min, purity 99.17%.

***Ethyl 6-(4-(5-methyl-4-(quinolin-6-yloxy)-1H-pyrazol-3-yl)phenoxy)hexanoate (6c)***. ^1^H NMR (400 MHz, DMSO-*d*_6_) *δ* 8.75 (dd, *J* = 4.0, 1.6 Hz, 1H, Quinoline-CH), 8.22 (dd, *J* = 8.4, 1.6 Hz, 1H, Quinoline-CH), 8.02 (d, *J* = 9.2 Hz, 1H, Quinoline-CH), 7.69–7.58 (m, 3H, Ether-Ph-CH, Quinoline-CH), 7.43 (dd, *J* = 8.4, 4.0 Hz, 1H, Quinoline-CH), 7.19 (d, *J* = 2.8 Hz, 1H, Quinoline-CH), 6.88 (s, 2H, Ether-Ph-CH), 4.01 (q, *J* = 7.2 Hz, 2H, Ester-CH_2_), 3.87 (t, *J* = 6.4 Hz, 2H, Ether-CH_2_), 2.26 (t, *J* = 7.2 Hz, 2H, Ester-CH_2_), 2.07 (s, 3H, Pyrazole-CH_3_), 1.64 (p, *J* = 6.8 Hz, 2H, CH_2_), 1.53 (p, *J* = 7.2 Hz, 2H, CH_2_), 1.34 (p, *J* = 7.6 Hz, 2H, CH_2_), 1.13 (t, *J* = 7.2 Hz, 3H, CH_3_). ^13^C NMR (101 MHz, DMSO-*d*_6_) *δ* 173.3, 156.4, 149.1, 144.7, 141.0, 137.2, 135.6, 135.3, 132.5, 131.6, 129.3, 126.9 (2 × C), 124.5, 122.3, 121.4, 115.1 (2 × C), 108.8, 67.7, 60.1, 33.9, 28.7, 25.5, 24.6, 14.6, 8.7. ESI-HRMS *m/z*: calcd for C_27_H_30_O_4_N_3_^+^ [M + H]^+^, 460.2231; found, 460.2235. HPLC: *t*_R_ 10 min, purity 99.71%.

***2-(4-(5-Methyl-4-(quinolin-6-yloxy)-1H-pyrazol-3-yl)phenoxy)acetonitrile (6d)***. ^1^H NMR (400 MHz, DMSO-*d*_6_) *δ* 8.80–8.73 (m, 1H, Quinoline-CH), 8.22 (d, *J* = 10.4 Hz, 1H, Quinoline-CH), 8.03 (d, *J* = 9.2 Hz, 1H, Quinoline-CH), 7.74 (d, *J* = 8.4 Hz, 2H, Ether-Ph-CH), 7.63 (dd, *J* = 9.2, 2.8 Hz, 1H, Quinoline-CH), 7.43 (dd, *J* = 8.4, 4.0 Hz, 1H, Quinoline-CH), 7.21 (d, *J* = 2.8 Hz, 1H, Quinoline-CH), 7.15–6.98 (m, 2H, Ether-Ph-CH), 5.12 (s, 2H, CH_2_), 2.07 (s, 3H, Pyrazole-CH_3_). ^13^C NMR (101 MHz, DMSO-*d*_6_) *δ* 156.4, 149.2, 144.7, 141.1, 137.2, 135.6, 135.3, 132.6, 131.7, 129.3, 127.2 (2 × C), 124.5, 122.3, 121.4, 117.0, 115.4 (2 × C), 108.8, 53.8, 8.7. ESI-HRMS *m/z*: calcd for C_21_H_17_O_2_N_4_^+^ [M + H]^+^, 357.1346; found, 357.1348. HPLC: *t*_R_ 10 min, purity 97.06%.

***6-((3-(4-(Cyclopentylmethoxy)phenyl)-5-methyl-1H-pyrazol-4-yl)oxy)quinoline (6e).*** ^1^H NMR (400 MHz, DMSO-*d*_6_) *δ* 8.76 (dd, *J* = 4.4, 1.6 Hz, 1H, Quinoline-CH), 8.22 (d, *J* = 8.4 Hz, 1H, Quinoline-CH), 8.02 (d, *J* = 9.2 Hz, 1H, Quinoline-CH), 7.69–7.55 (m, 3H, Ether-Ph-CH, Quinoline-CH), 7.44 (dd, *J* = 8.4, 4.0 Hz, 1H, Quinoline-CH), 7.18 (d, *J* = 2.8 Hz, 1H, Quinoline-CH), 6.96–6.86 (m, 2H, Ether-Ph-CH), 3.77 (s, 2H, CH_2_), 2.29–2.18 (m, 1H, Cyclopentane-CH), 2.06 (s, 3H, Pyrazole-CH_3_), 1.75–1.68 (m, 2H, Cyclopentane-CH_2_), 1.61–1.43 (m, 4H, Cyclopentane-CH_2_), 1.27 (q, *J* = 6.8 Hz, 2H, Cyclopentane-CH_2_). ^13^C NMR (101 MHz, DMSO-*d*_6_) *δ* 156.5, 149.2, 144.7, 141.2, 137.2, 135.6, 135.3, 132.5, 131.6, 129.3, 127.0 (2 × C), 124.5, 122.3, 120.5, 114.9 (2 × C), 108.8, 72.0, 38.9, 29.4 (2 × C), 25.4 (2 × C), 8.7. ESI-HRMS *m/z*: calcd for C_25_H_26_O_2_N_3_^+^ [M + H]^+^, 400.2020; found, 400.2019. HPLC: *t*_R_ 7 min, purity 97.07%.

***6-((5-Methyl-3-(4-((tetrahydro-2H-pyran-4-yl)methoxy)phenyl)-1H-pyrazol-4-yl)oxy)quinoline (6f).*** ^1^H NMR (400 MHz, DMSO-*d*_6_) *δ* 8.76 (s, 1H, Quinoline-CH), 8.21 (d, *J* = 8.0 Hz, 1H, Quinoline-CH), 8.02 (d, *J* = 9.2 Hz, 1H, Quinoline-CH), 7.60 (dd, *J* = 9.2, 2.8 Hz, 3H, Ether-Ph-CH, Quinoline-CH), 7.43 (dd, *J* = 8.4, 4.0 Hz, 1H, Quinoline-CH), 7.18 (d, *J* = 2.8 Hz, 1H, Quinoline-CH), 6.92–6.87 (m, 2H, Ether-Ph-CH), 3.82 (dd, *J* = 10.4, 4.0 Hz, 2H, Tetrahydropyran-CH_2_), 3.76 (s, 2H, CH_2_), 3.33–3.21 (m, 2H, Tetrahydropyran-CH_2_), 2.06 (s, 3H, Pyrazole-CH_3_), 1.92 (s, 1H, Tetrahydropyran-CH), 1.60 (d, *J* = 16.0 Hz, 2H, Tetrahydropyran-CH_2_), 1.30–1.19 (m, 2H, Tetrahydropyran-CH_2_). ^13^C NMR (101 MHz, DMSO-*d*_6_) *δ* 156.5, 149.2, 144.7, 141.0, 137.2, 135.6, 135.3, 132.5, 131.6, 129.3, 127.0 (2 × C), 124.5, 122.3, 121.4, 115.0 (2 × C), 108.8, 72.4, 67.0 (2 × C), 34.8, 29.6 (2 × C), 8.7. ESI-HRMS *m/z*: calcd for C_25_H_26_O_3_N_3_^+^ [M + H]^+^, 416.1969; found, 416.1972. HPLC: *t*_R_ 12 min, purity 94.37%.

***Ethyl 2-(4-(5-methyl-4-(quinolin-6-yloxy)-1H-pyrazol-3-yl)phenoxy)acetate (6g).*** ^1^H NMR (400 MHz, DMSO-*d*_6_) *δ* 8.76 (dd, *J* = 4.4, 2.0 Hz, 1H, Quinoline-CH), 8.22 (d, *J* = 8.4 Hz, 1H, Quinoline-CH), 8.03 (d, *J* = 9.2 Hz, 1H, Quinoline-CH), 7.65 (s, 2H, Ether-Ph-CH), 7.60 (dd, *J* = 9.2, 2.8 Hz, 1H, Quinoline-CH), 7.44 (dd, *J* = 8.4, 4.0 Hz, 1H, Quinoline-CH), 7.19 (d, *J* = 2.8 Hz, 1H, Quinoline-CH), 6.94–6.88 (m, 2H, Ether-Ph-CH), 4.73 (s, 2H, CH_2_), 4.11 (q, *J* = 7.2 Hz, 2H, Ester-CH_2_), 2.10–2.03 (m, 3H, Pyrazole-CH_3_), 1.15 (t, *J* = 7.2 Hz, 3H, CH_3_). ^13^C NMR (101 MHz, DMSO-*d*_6_) *δ* 169.1, 157.4, 156.4, 149.2, 144.7, 140.9, 135.6, 132.6, 131.6, 129.3, 127.0 (2 × C), 126.2, 122.3, 121.4, 115.5 (2 × C), 115.1, 108.8, 65.0, 61.1, 14.4, 8.7. ESI-HRMS *m/z*: calcd for C_23_H_22_O_3_N_4_^+^ [M + H]^+^, 404.1610; found, 404.1610. HPLC: *t*_R_ 7 min, purity 97.47%.

***Methyl 2-(3-(4-(2-methoxy-2-oxoethoxy)phenyl)-5-methyl-4-(quinolin-6-yloxy)-1H-pyrazol-1-yl)acetate (6h).*** ^1^H NMR (400 MHz, MeOH-*d*_4_) *δ* 8.28 (dd, *J* = 4.0, 1.6 Hz, 1H, Quinoline-CH), 7.70 (dd, *J* = 8.4, 1.6 Hz, 1H, Quinoline-CH), 7.59 (dd, *J* = 9.2, 2.4 Hz, 1H, Quinoline-CH), 7.28 (d, *J* = 8.8 Hz, 2H, Ether-Ph-CH), 7.21 (dd, *J* = 9.2, 2.8 Hz, 1H, Quinoline-CH), 7.02 (dd, *J* = 8.4, 4.4 Hz, 1H, Quinoline-CH), 6.75 (d, *J* = 2.8 Hz, 1H, Quinoline-CH), 6.42 (d, *J* = 9.2 Hz, 2H, Ether-Ph-CH), 4.65 (d, *J* = 2.8 Hz, 2H, CH_2_), 4.01 (s, 2H, Ether-CH_2_), 3.42 (s, 3H, CH_3_), 3.30 (s, 3H, CH_3_), 1.72 (s, 3H, Pyrazole-CH_3_). ^13^C NMR (101 MHz, MeOH-*d*_4_) *δ* 169.9, 168.8, 157.8, 156.6, 148.1, 143.8, 141.8, 136.0, 133.6, 133.0, 129.9, 129.4, 127.2, 124.7 (2 × C), 121.7, 121.3, 114.3 (2 × C), 108.5, 64.4, 51.9, 51.2, 50.5, 7.1. ESI-HRMS *m/z*: calcd for C_25_H_24_O_7_N_3_^+^ [M + H]^+^, 462.1659; found, 462.1658. HPLC: *t*_R_ 10 min, purity 99.10%.

***2-(3-(4-(Carboxymethoxy)phenyl)-5-methyl-4-(quinolin-6-yloxy)-1H-pyrazol-1-yl)acetic acid (6i).*** ^1^H NMR (400 MHz, DMSO-*d*_6_) *δ* 8.76 (dd, *J* = 4.4, 1.6 Hz, 1H, Quinoline-CH), 8.16 (dd, *J* = 8.4, 1.6 Hz, 1H, Quinoline-CH), 8.03 (d, *J* = 9.2 Hz, 1H, Quinoline-CH), 7.69–7.58 (m, 3H, Ether-Ph-CH, Quinoline-CH), 7.44 (dd, *J* = 8.4, 4.0 Hz, 1H, Quinoline-CH), 7.19 (d, *J* = 2.8 Hz, 1H, Quinoline-CH), 6.85 (d, *J* = 9.2 Hz, 2H, Ether-Ph-CH), 5.00 (s, 2H, CH_2_), 4.61 (s, 2H, Ether-CH_2_), 2.07 (s, 3H, Pyrazole-CH_3_). ^13^C NMR (400 MHz, DMSO-*d*_6_) *δ* 171.4, 170.8, 158.5, 157.2, 150.1, 145.5, 141.1, 136.3, 134.0, 133.5, 132.5, 130.0, 127.9 (2 × C), 125.9, 123.2, 122.0, 115.8 (2 × C), 109.7, 65.6, 52.9, 9.6. ESI-HRMS *m/z*: calcd for C_23_H_20_O_7_N_3_^+^ [M + H]^+^, 434.1346; found, 434.1332. HPLC: *t*_R_ 10 min, purity 98.93%.

***Ethyl 2-(3-(4-(2-ethoxy-2-oxoethoxy)phenyl)-5-methyl-4-(quinolin-6-yloxy)-1H-pyrazol-1-yl)acetate (6j).*** ^1^H NMR (400 MHz, DMSO-*d*_6_) *δ* 8.77 (dd, *J* = 4.0, 1.6 Hz, 1H, Quinoline-CH), 8.17 (d, *J* = 8.4 Hz, 1H, Quinoline-CH), 8.04 (d, *J* = 9.2 Hz, 1H, Quinoline-CH), 7.66 (d, *J* = 8.4 Hz, 2H, Ether-Ph-CH), 7.60 (dd, *J* = 9.2, 2.8 Hz, 1H, Quinoline-CH), 7.45 (dd, *J* = 8.4, 4.0 Hz, 1H, Quinoline-CH), 7.19 (d, *J* = 2.8 Hz, 1H, Quinoline-CH), 6.88 (d, *J* = 8.4 Hz, 2H, Ether-Ph-CH), 5.14 (s, 2H, CH_2_), 4.72 (s, 2H, Ether-CH_2_), 4.01 (q, *J* = 7.2 Hz, 2H, Ester-CH_3_), 4.11 (q, *J* = 7.2 Hz, 2H, Ester-CH_3_), 2.08 (s, 3H, Pyrazole-CH_3_), 1.25 (t, *J* = 7.2 Hz, 3H, CH_3_), 1.14 (t, *J* = 7.2 Hz, 3H, CH_3_). ^13^C NMR (101 MHz, DMSO-*d*_6_) *δ* 169.1, 168.5, 157.6, 156.3, 149.3, 144.8, 140.6, 135.5, 133.4, 132.8, 131.7, 129.2, 127.1 (2 × C), 125.2, 122.5, 121.1, 115.2 (2 × C), 108.9, 65.0, 61.8, 61.1, 51.3, 14.5, 14.4, 8.7. ESI-HRMS *m/z*: calcd for C_27_H_28_O_7_N_3_^+^ [M + H]^+^, 490.1972; found, 490.1972. HPLC: *t*_R_ 7 min, purity 95.42%.

***2-(4-(5-Methyl-1-(2-oxo-2-phenylethyl)-4-(quinolin-6-yloxy)-1H-pyrazol-3-yl)phenoxy)-1-phenylethan-1-one (6k).*** ^1^H NMR (400 MHz, DMSO-*d*_6_) *δ* 8.78 (dd, *J* = 4.2, 1.7 Hz, 1H, Quinoline-CH), 8.20 (dd, *J* = 8.4, 1.6 Hz, 1H, Quinoline-CH), 8.13 (dd, *J* = 8.4, 1.6 Hz, 2H, Carbonyl-Ph-CH), 8.05 (d, *J* = 9.2 Hz, 1H, Quinoline-CH), 8.01–7.95 (m, 2H, Carbonyl-Ph-CH), 7.74 (d, *J* = 7.2 Hz, 1H, Carbonyl-Ph-CH), 7.69–7.60 (m, 6H, Ether-Ph-CH, Carbonyl-Ph-CH, Quinoline-CH), 7.53 (t, *J* = 7.6 Hz, 2H, Carbonyl-Ph-CH), 7.47 (dd, *J* = 8.4, 4.0 Hz, 1H, Quinoline-CH), 7.25 (d, *J* = 2.8 Hz, 1H, Quinoline-CH), 6.93 (d, *J* = 8.8 Hz, 2H, Ether-Ph-CH), 5.97 (s, 2H, Ether-CH_2_), 5.54 (s, 2H, CH_2_), 2.04 (s, 3H, Pyrazole-CH_3_). ^13^C NMR (101 MHz, DMSO-*d*_6_) *δ* 194.8, 194.1, 157.9, 156.5, 149.3, 144.8, 140.5, 137.2, 135.5, 135.3, 134.8, 134.8, 134.6, 134.2, 133.7, 132.8, 131.7, 129.4 (2 × C), 129.3, 128.8, 128.3 (2 × C), 127.0 (2 × C), 125.1, 124.5, 122.5, 121.1, 115.3 (2 × C), 108.9, 70.5, 57.2, 8.7. ESI-HRMS *m/z*: calcd for C_35_H_28_O_4_N_3_^+^ [M + H]^+^, 554.2074; found, 554.2076. HPLC: *t*_R_ 10 min, purity 95.24%.

#### Synthesis of compound 7

To a solution of intermediate **5** (100 mg, 0.63 mmol, 1 equiv.) in anhydrous DMF (10 ml), K_2_CO_3_ (87 mg, 1.26 mmol, 2 equiv.) was added methyl bromoacetate (48 mg, 0.63 mmol, 1 equiv.). The reaction mixture was stirred at room temperature overnight. Upon completion, the reaction mixture was diluted with ethyl acetate, and the organic layer was washed with water and brine. The organic layer was dried over Na_2_SO_4_, filtered, and concentrated under reduced pressure. The residue was purified by silica gel column chromatography to obtain intermediate **7** as a yellow solid. Yield: 82%.

***Methyl 2-(4-(5-methyl-4-(quinolin-6-yloxy)-1H-pyrazol-3-yl)phenoxy)acetate (7).*** ^1^H NMR (400 MHz, DMSO-*d*_6_) *δ* 8.75 (dd, J = 4.4, 1.6 Hz, 1H, Quinoline-CH), 8.22 (d, J = 8.4 Hz, 1H, Quinoline-CH), 8.02 (d, J = 9.2 Hz, 1H, Quinoline-CH), 7.76–7.57 (m, 3H, Ether-Ph-CH, Quinoline-CH), 7.44 (dd, J = 8.4, 4.0 Hz, 1H, Quinoline-CH), 7.18 (d, J = 2.8 Hz, 1H, Quinoline-CH), 6.90 (s, 2H, Ether-Ph-CH), 4.74 (s, 2H, Ether-CH_2_), 3.65 (s, 3H, Ester-CH_3_), 2.06 (s, 3H, Pyrazole-CH_3_). ^13^C NMR (101 MHz, DMSO-*d*_6_) *δ* 169.1, 157.4, 156.4, 149.2, 144.7, 140.9, 135.6, 132.6, 131.6, 129.3, 127.0 (2 × C), 126.2, 122.3, 121.4, 115.5 (2 × C), 115.1, 108.8, 65.0, 61.1, 8.7. ESI-HRMS *m/z*: calcd for C_22_H_20_O_4_N_3_^+^ [M + H]^+^, 390.1454; found, 390.1447. HPLC: *t*_R_ 7 min, purity 95.30%.

#### Synthesis of compound 8

To a solution of intermediate **7** (100 mg, 0.2 mmol, 1 equiv.) in 1,4-dioxane (10 ml), NaOH (30 mg, 0.6 mmol, 3 equiv.) was added, and the mixture was stirred at 60 °C for 4 h. After the Thin-Layer Chromatography(TLC) analysis indicated complete conversion, the volatiles were evaporated in vacuum. The aqueous mixture was adjusted to acidic (pH = 6) with diluted HCl. The resulting slurry mixture was filtered and dried to obtain intermediate **8** as a yellow solid. Yield: 96%.

***2-(4-(5-Methyl-4-(quinolin-6-yloxy)-1H-pyrazol-3-yl)phenoxy)acetic acid (8).*** ^1^H NMR (400 MHz, DMSO-*d*_6_) *δ* 8.76 (dd, *J* = 4.0, 1.6 Hz, 1H, Quinoline-CH), 8.24 (d, *J* = 8.4 Hz, 1H, Quinoline-CH), 8.03 (d, *J* = 9.2 Hz, 1H, Quinoline-CH), 7.68–7.58 (m, 3H, Ether-Ph-CH, Quinoline-CH), 7.45 (dd, *J* = 8.4, 4.0 Hz, 1H, Quinoline-CH), 7.20 (d, *J* = 2.8 Hz, 1H, Quinoline-CH), 6.88 (d, *J* = 6.8 Hz, 2H, Ether-Ph-CH), 4.63 (s, 2H, Ether-CH_2_), 2.06 (s, 3H, Pyrazole-CH_3_). ^13^C NMR (101 MHz, DMSO-*d*_6_) *δ* 170.5, 157.7, 156.5, 149.0, 144.4, 137.2, 135.9, 135.4, 132.5, 131.4, 129.3, 126.9 (2 × C), 124.2, 122.3, 121.5, 115.2 (2 × C), 108.9, 64.9, 9.7. ESI-HRMS *m/z*: calcd for C_21_H_18_O_4_N_3_^+^ [M + H]^+^, 376.1297; found, 376.1292. HPLC: *t*_R_ 5 min, purity 99.90%.

#### Synthesis of compounds 9a–9l

To a solution of intermediate 7 (50 mg, 0.13 mmol, 1.0 equiv.) in DMF (2 ml) was added O-(7-azabenzotriazol-1-yl)-*N*,*N*,*N*′,*N*′-tetramethyl uranium hexafluorophosphate (HATU) (58.5 mg, 0.13 mmol, 1.0 equiv.), amine (0.13 mmol, 1.0 equiv.), and *N*,*N*-diisopropylethylamine (DIPEA) (40 mg, 0.26 mmol, 2.0 equiv.). The resulting mixture was stirred for 12 h at room temperature and quenched with water. The organic layer was collected. After extraction with ethyl acetate, the combined organic fractions were washed with brine and concentrated under reduced pressure. The residue was purified by flash chromatography on silica gel using eluents to give compounds **9a–9l**.

***N-(benzo[d]thiazol-6-yl)-2-(4-(5-methyl-4-(quinolin-6-yloxy)-1H-pyrazol-3-yl)phenoxy)acetamide (9a).*** ^1^H NMR (400 MHz, DMSO-*d*_6_) *δ* 10.38 (s, 1H, NH), 9.28 (s, 1H, Benzothiazole-CH), 8.75 (dd, *J* = 4.0, 1.6 Hz, 1H, Quinoline-CH), 8.52 (d, *J* = 2.0 Hz, 1H, Benzothiazole-CH), 8.22 (d, *J* = 8.8 Hz, 1H, Quinoline-CH), 8.02 (dd, *J* = 9.2, 2.8 Hz, 2H, H_22_, Quinoline-CH), 7.66 (d, *J* = 6.4 Hz, 2H, Ether-Ph-CH), 7.64–7.59 (m, 2H, Quinoline-CH, Benzothiazole-CH), 7.44 (dd, *J* = 8.4, 4.0 Hz, 1H, Quinoline-CH), 7.19 (d, *J* = 2.8 Hz, 1H, Quinoline-CH), 6.99 (s, 2H, Ether-Ph-CH), 4.72 (s, 2H, Ether-CH_2_), 2.09 (s, 3H, Pyrazole-CH_3_). ^13^C NMR (101 MHz, DMSO-*d*_6_) *δ* 167.2, 157.6, 156.4, 155.5, 149.9, 149.2, 144.7, 137.2, 136.5, 135.6, 135.4, 134.6, 132.6, 131.6, 129.3, 127.0 (2 × C), 124.2, 123.4, 122.3, 121.4, 119.7, 115.3 (2 × C), 112.8, 108.8, 67.5, 8.7. ESI-HRMS *m/z*: calcd for C_28_H_22_O_3_N_5_S^+^ [M + H]^+^, 508.1438; found, 508.1436. HPLC: *t*_R_ 7 min, purity 95.38%.

***2-(4-(5-Methyl-4-(quinolin-6-yloxy)-1H-pyrazol-3-yl)phenoxy)-N-(pyridin-2-yl)acetamide (9b).*** ^1^H NMR (400 MHz, DMSO-*d*_6_) *δ* 10.47 (s, 1H, NH), 8.75 (dd, *J* = 4.0, 1.6 Hz, 1H, Quinoline-CH), 8.31 (dd, *J* = 5.6, 2.0 Hz, 1H, Pyridine-CH), 8.22 (dd, *J* = 8.4, 2.0 Hz, 1H, Quinoline-CH), 8.01 (d, *J* = 9.2 Hz, 2H, Pyridine-CH, Quinoline-CH), 7.77 (t, *J* = 8.0 Hz, 1H, Pyridine-CH), 7.65 (d, *J* = 8.4 Hz, 2H, Ether-Ph-CH), 7.62–7.59 (m, 1H, Quinoline-CH), 7.43 (dd, *J* = 8.4, 4.0 Hz, 1H, Quinoline-CH), 7.19 (d, *J* = 2.8 Hz, 1H, Quinoline-CH), 7.15–7.08 (m, 1H, Pyridine-CH), 6.94 (d, *J* = 7.2 Hz, 2H, Ether-Ph-CH), 4.75 (s, 2H, Ether-CH_2_), 2.06 (s, 3H, Pyrazole-CH_3_). ^13^C NMR (101 MHz, DMSO-*d*_6_) *δ* 167.6, 157.8, 156.0, 151.8, 149.2, 148.5, 144.7, 138.8, 137.2, 135.6, 135.4, 132.6, 131.6, 129.3, 126.9 (2 × C), 124.2, 121.4, 120.3, 115.3 (2 × C), 114.0, 112.3, 108.8, 67.0, 8.8. ESI-HRMS *m/z*: calcd for C_26_H_22_O_3_N_5_S^+^ [M + H]^+^, 451.2644; found, 451.2632. HPLC: *t*_R_ 7 min, purity 96.12%.

***N-(1-methyl-1H-pyrazol-3-yl)-2-(4-(5-methyl-4-(quinolin-6-yloxy)-1H-pyrazol-3-yl)phenoxy)acetamide (9c).*** ^1^H NMR (400 MHz, DMSO-*d*_6_) *δ* 10.49 (s, 1H, NH), 8.75 (dd, *J* = 4.4, 1.6 Hz, 1H, Quinoline-CH), 8.22 (dd, *J* = 8.4, 1.6 Hz, 1H, Quinoline-CH), 8.02 (d, *J* = 9.2 Hz, 1H, Quinoline-CH), 7.69–7.58 (m, 3H, Ether-Ph-CH, Quinoline-CH), 7.54 (d, *J* = 2.0 Hz, 1H, Pyrazole-CH), 7.43 (dd, *J* = 8.4, 4.0 Hz, 1H, Quinoline-CH), 7.18 (d, *J* = 2.8 Hz, 1H, Quinoline-CH), 6.92 (d, *J* = 8.4 Hz, 2H, Ether-Ph-CH), 6.39 (d, *J* = 2.0 Hz, 1H, Pyrazole-CH), 4.62 (s, 2H, Ether-CH_2_), 3.72 (s, 3H, Pyrazole-CH_3_), 2.06 (s, 3H, Pyrazole-CH_3_). ^13^C NMR (101 MHz, DMSO-*d*_6_) *δ* 165.9, 157.8, 156.4, 149.2, 146.5, 144.7, 137.2, 135.6, 135.4, 132.6, 131.6, 131.5, 129.3, 126.9 (2 × C), 124.2, 122.3, 121.4, 115.3 (2 × C), 108.8, 97.2, 66.9, 38.8, 8.9. ESI-HRMS *m/z*: calcd for C_25_H_23_O_3_N_6_^+^ [M + H]^+^, 455.1832; found, 455.1824. HPLC: *t*_R_ 10 min, purity 99.96%.

***2-(4-(5-Methyl-4-(quinolin-6-yloxy)-1H-pyrazol-3-yl)phenoxy)-N-(thiazol-2-yl)acetamide (9d).*** ^1^H NMR (400 MHz, DMSO-*d*_6_) *δ* 8.75 (dd, *J* = 4.4, 1.6 Hz, 1H, Quinoline-CH), 8.22 (dd, *J* = 8.4, 1.6 Hz, 1H, Quinoline-CH), 8.01 (d, *J* = 9.2 Hz, 1H, Quinoline-CH), 7.68–7.57 (m, 3H, Ether-Ph-CH, Quinoline-CH), 7.43 (dd, *J* = 6.8, 3.2 Hz, 2H, Thiazole-CH, Quinoline-CH), 7.17 (dd, *J* = 9.6, 3.2 Hz, 2H, Thiazole-CH, Quinoline-CH), 6.93 (d, *J* = 8.8 Hz, 2H, Ether-Ph-CH), 4.79 (s, 2H, Ether-CH_2_), 2.07 (d, *J* = 8.0 Hz, 3H, Pyrazole-CH_3_. ^13^C NMR (101 MHz, DMSO-*d*_6_) *δ* 167.5, 158.8, 157.8, 156.4, 149.2, 144.7, 138.0, 137.2, 135.6, 135.4, 132.5, 131.6, 129.3, 126.9 (2 × C), 124.2, 122.3, 121.4, 115.3 (2 × C), 113.9, 107.0, 66.7, 31.2. ESI-HRMS *m/z*: calcd for C_24_H_20_O_3_N_5_S^+^ [M + H]^+^, 458.1287; found, 458.1273. HPLC: *t*_R_ 5 min, purity 99.16%.

***N-((1-ethylpyrrolidin-2-yl)methyl)-2-(4-(5-methyl-4-(quinolin-6-yloxy)-1H-pyrazol-3-yl)phenoxy)acetamide (9e).*** ^1^H NMR (400 MHz, DMSO-*d*_6_) *δ* 8.75 (dd, *J* = 4.0, 1.6 Hz, 1H, Quinoline-CH), 8.21 (d, *J* = 8.4 Hz, 1H, Quinoline-CH), 8.02 (d, *J* = 9.2 Hz, 1H, Quinoline-CH), 7.94 (s, 1H, NH), 7.71–7.57 (m, 3H, Ether-Ph-CH, Quinoline-CH), 7.43 (dd, *J* = 8.4, 4.0 Hz, 1H, Quinoline-CH), 7.17 (d, *J* = 2.8 Hz, 1H, Quinoline-CH), 6.92 (d, *J* = 8.4 Hz, 2H, Ether-Ph-CH), 4.47 (s, 2H, Ether-CH_2_), 3.29–3.25 (m, 1H, Amide-CH_2_), 3.05 (s, 2H, Pyrrolidine-CH_2_), 2.85 (s, 1H, Pyrrolidine-CH), 2.30 (s, 2H, Pyrrolidine-CH_2_), 2.07 (s, 3H, Pyrazole-CH_3_), 1.77–1.37 (m, 4H, Pyrrolidine-CH_2_), 0.98 (t, *J* = 7.2 Hz, 3H, CH_3_).13C NMR (101 MHz, DMSO-*d*_6_) *δ* 168.5, 157.5, 156.4, 149.2, 144.7, 137.2, 135.6, 135.4, 132.6, 131.6, 129.3, 126.9 (2 × C), 124.2, 122.3, 121.4, 115.4 (2 × C), 108.8, 67.3, 64.5, 53.4, 48.1, 40.8, 28.2, 22.4, 12.7, 8.3. ESI-HRMS *m/z*: calcd for C_28_H_32_O_3_N_5_^+^ [M + H]^+^, 486.2505; found, 486.2495. HPLC: *t*_R_ 7 min, purity 99.96%.

***N-(1H-indol-5-yl)-2-(4-(5-methyl-4-(quinolin-6-yloxy)-1H-pyrazol-3-yl)phenoxy)acetamide (9f).*** ^1^H NMR (400 MHz, MeOH-*d*_4_) *δ* 8.66 (dd, *J* = 4.4, 1.6 Hz, 1H, Quinoline-CH), 8.09 (dd, *J* = 8.4, 1.6 Hz, 1H, Quinoline-CH), 7.99 (d, *J* = 9.2 Hz, 1H, Quinoline-CH), 7.76–7.62 (m, 3H, Ether-Ph-CH, Quinoline-CH), 7.59 (dd, *J* = 9.2, 2.8 Hz, 1H, Indole-CH), 7.39 (dd, *J* = 8.4, 4.4 Hz, 1H, Quinoline-CH), 7.29 (d, *J* = 8.8 Hz, 1H, Indole-CH), 7.21 (d, *J* = 3.2 Hz, 1H, Quinoline-CH), 7.17–7.10 (m, 2H, Indole-CH), 7.00 (d, *J* = 8.8 Hz, 2H, Ether-Ph-CH), 6.37 (dd, *J* = 3.2, 0.8 Hz, 1H, Indole-CH), 4.65 (s, 2H, Ether-CH_2_), 2.13 (s, 3H, Pyrazole-CH_3_). ^13^C NMR (101 MHz, MeOH-*d*_4_) *δ* 167.6, 157.7, 156.7, 148.0, 143.8, 137.2, 136.0, 135.4, 134.0, 132.6, 129.8, 129.4, 129.3, 128.8, 127.9, 126.9 (2 × C), 125.3, 124.2, 121.6, 121.4, 116.2, 114.8 (2 × C), 113.1, 108.4, 101.1, 67.1, 13.1. ESI-HRMS *m/z*: calcd for C_29_H_24_O_3_N_5_^+^ [M + H]^+^, 490.1979; found, 490.1972. HPLC: *t*_R_ 10 min, purity 99.91%.

***2-(4-(5-Methyl-4-(quinolin-6-yloxy)-1H-pyrazol-3-yl)phenoxy)-N-(pyridin-2-ylmethyl)acetamide (9g).*** ^1^H NMR (400 MHz, DMSO-*d*_6_) *δ* 8.76 (dd, *J* = 4.4, 1.6 Hz, 1H, Quinoline-CH), 8.67 (t, *J* = 6.4 Hz, 1H, Pyridine-CH), 8.44 (d, *J* = 2.0 Hz, 1H, Pyridine-CH), 8.36 (dd, *J* = 4.8, 1.6 Hz, 1H, Pyridine-CH), 8.22 (dd, *J* = 8.4, 1.6 Hz, 1H, Quinoline-CH), 8.02 (d, *J* = 9.2 Hz, 1H, Quinoline-CH), 7.68–7.52 (m, 4H, Ether-Ph-CH, Quinoline-CH_,_ NH), 7.44 (dd, *J* = 8.4, 4.0 Hz, 1H, Quinoline-CH), 7.24–7.16 (m, 2H, Pyridine-CH, Quinoline-CH), 6.92 (s, 2H, Ether-Ph-CH), 4.51 (s, 2H, Ether-CH_2_), 4.31 (d, *J* = 6.0 Hz, 2H, Amine-CH_2_), 2.07 (s, 3H, Pyrazole-CH_3_). ^13^C NMR (151 MHz, DMSO-*d*_6_) *δ* 168.4, 157.6, 156.4, 149.2, 148.5, 144.7, 141.0, 137.2, 135.6, 135.4, 135.2, 132.6, 131.6, 129.3, 126.9 (2 × C), 126.2, 124.2, 123.8, 122.3, 121.4, 115.4 (2 × C), 108.8, 67.4, 11.1, 8.7. ESI-HRMS *m/z*: calcd for C_27_H_24_O_3_N_5_^+^ [M + H]^+^, 466.1879; found, 466.1865. HPLC: *t*_R_ 10 min, purity 99.31%.

***2-(4-(5-Methyl-4-(quinolin-6-yloxy)-1H-pyrazol-3-yl)phenoxy)-N-(pyridin-3-ylmethyl)acetamide (9h).*** ^1^H NMR (400 MHz, DMSO-*d*_6_) *δ* 8.76 (dd, *J* = 4.4, 1.6 Hz, ^1^H, Quinoline-CH), 8.68 (t, *J* = 6.0 Hz, 1H, Pyridine-CH), 8.44 (d, *J* = 2.4 Hz, 1H, Pyridine-CH), 8.36 (dd, *J* = 4.8, 1.6 Hz, 1H, Pyridine-CH), 8.22 (d, *J* = 8.4 Hz, 1H, Quinoline-CH), 8.03 (d, *J* = 9.2 Hz, 1H, Quinoline-CH), 7.66 (s, 2H, Ether-Ph-CH), 7.63–7.59 (m, 1H, Quinoline-CH), 7.56 (d, *J* = 8.0 Hz, 1H, NH), 7.44 (dd, *J* = 8.4, 4.4 Hz, 1H, Quinoline-CH), 7.21 (s, 1H, Pyridine-CH), 7.19 (d, *J* = 2.8 Hz, 1H, Quinoline-CH), 7.04–6.85 (m, 2H, Ether-Ph-CH), 4.52 (s, 2H, Ether-CH_2_), 4.31 (d, *J* = 6.0 Hz, 2H, Amine-CH_2_), 2.07 (s, 3H, Pyrazole-CH_3_). ^13^C NMR (101 MHz, DMSO-*d*_6_) *δ* 168.4, 163.4, 157.5, 156.4, 149.2, 148.5, 144.7, 141.5, 137.2, 135.6, 135.4, 135.2, 132.6, 131.6, 129.3, 126.9 (2 × C), 124.2, 123.8, 122.3, 121.4, 115.2 (2 × C), 108.8, 67.3, 11.1, 8.8. ESI-HRMS *m/z*: calcd for C_27_H_24_O_3_N_5_^+^ [M + H]^+^, 466.1879; found, 466.1870. HPLC: *t*_R_ 7 min, purity 95.68%.

***2-(4-(5-methyl-4-(quinolin-6-yloxy)-1H-pyrazol-3-yl)phenoxy)-N-(pyrazin-2-ylmethyl)acetamide (9i).*** ^1^H NMR (600 MHz, DMSO-*d*_6_) *δ* 8.8–8.7 (m, 2H, Quinoline-CH), 8.5 (d, *J* = 1.8 Hz, 1H, Pyrimidine-CH), 8.5 (t, *J* = 2.4 Hz, 1H, Pyrimidine-CH), 8.5 (d, *J* = 2.4 Hz, 1H, Pyrimidine-CH), 8.2 (dd, *J* = 8.4, 1.8 Hz, 1H, Quinoline-CH), 8.0 (d, *J* = 9.0 Hz, 1H, Quinoline-CH), 7.7 (s, 2H, Ether-Ph-CH), 7.6 (dd, *J* = 9.0, 1.8 Hz, 1H, Quinoline-CH), 7.4 (dd, *J* = 8.4, 4.2 Hz, 1H, Quinoline-CH), 7.2 (d, *J* = 2.4 Hz, 1H, Quinoline-CH), 7.0 (s, 2H, Ether-Ph-CH), 4.6 (s, 2H, Ether-CH_2_), 4.5 (d, *J* = 6.0 Hz, 2H, Amine-CH_2_), 2.1 (s, 3H, Pyrazole-CH_3_). ^13^C NMR (151 MHz, DMSO-*d*_6_) *δ* 168.6, 157.6, 156.4, 154.3, 149.2, 144.7, 144.2, 143.8, 143.6, 137.2, 135.6, 135.3, 132.6, 131.6, 129.3, 126.9 (2 × C), 124.2, 122.3, 121.4, 115.5 (2 × C), 108.8, 67.3, 42.4, 8.9. ESI-HRMS *m/z*: calcd for C_26_H_23_O_3_N_6_^+^ [M + H]^+^, 467.1826; found, 467.1828. HPLC: *t*_R_ 5 min, purity 97.14%.

***N-((6-Methoxypyridin-3-yl)methyl)-2-(4-(5-methyl-4-(quinolin-6-yloxy)-1H-pyrazol-3-yl)phenoxy)acetamide (9j).*** ^1^H NMR (600 MHz, DMSO-*d*_6_) *δ* 8.76 (d, *J* = 2.4 Hz, 1H, Quinoline-CH), 8.59 (t, *J* = 6.0 Hz, 1H, Pyridine-CH), 8.22 (d, *J* = 9.0 Hz, 1H, Quinoline-CH), 8.03 (d, *J* = 9.0 Hz, 2H, Quinoline-CH), 7.73–7.58 (m, 3H, Ether-Ph-CH, Quinoline-CH), 7.54 (dd, *J* = 8.4, 2.4 Hz, 1H, Pyridine-CH), 7.44 (dd, *J* = 8.4, 4.2 Hz, 1H, Quinoline-CH), 7.20 (d, *J* = 3.0 Hz, 1H, Quinoline-CH), 6.92 (s, 2H, Ether-Ph-CH), 6.72 (d, *J* = 8.4 Hz, 1H, Pyridine-CH), 4.49 (s, 2H, Ether-CH_2_), 4.24 (d, *J* = 6.0 Hz, 2H, Amine-CH_2_), 3.80 (s, 3H, Methoxy-CH_3_), 2.07 (s, 3H, Pyrazole-CH_3_). ^13^C NMR (151 MHz, DMSO-*d*_6_) *δ* 168.1, 163.1, 157.5, 156.4, 149.2, 146.2, 144.7, 139.2, 137.2, 135.6, 135.3, 132.6, 131.6, 129.3, 128.3, 126.9 (2 × C), 124.2, 122.3, 121.4, 115.3 (2 × C), 110.6, 108.8, 67.4, 67.1, 53.5, 8.7. ESI-HRMS *m/z*: calcd for C_28_H_25_O_4_N_5_^+^ [M + H]^+^, 496.1979; found, 496.1984. HPLC: *t*_R_ 7 min, purity 95.78%.

***2-(4-(5-Methyl-4-(quinolin-6-yloxy)-1H-pyrazol-3-yl)phenoxy)-N-((tetrahydro-2H-pyran-4-yl)methyl)acetamide (9k).*** ^1^H NMR (600 MHz, DMSO-*d*_6_) *δ* 8.76 (d, *J* = 4.2 Hz, 1H, Quinoline-CH), 8.22 (d, *J* = 8.4 Hz, 1H, Quinoline-CH), 8.01–8.05 (m, 2H, Quinoline-CH), 7.75–7.56 (m, 3H, Ether-Ph-CH, Quinoline-CH), 7.43 (dd, *J* = 8.4, 4.2 Hz, 1H, Quinoline-CH), 7.19 (s, 1H, Quinoline-CH), 6.93 (s, 2H, Ether-Ph-CH), 4.45 (s, 2H, Ether-CH_2_), 3.84–3.69 (m, 2H, Amine-CH_2_), 3.14 (t, *J* = 11.8 Hz, 2H, Tetrahydropyran-CH_2_), 2.97 (t, *J* = 6.6 Hz, 2H, Tetrahydropyran-CH_2_), 2.06 (s, 3H, Pyrazole-CH_3_), 1.61 (s, 1H, Tetrahydropyran-CH), 1.43 (d, *J* = 12.4 Hz, 2H, Tetrahydropyran-CH_2_), 1.13–1.03 (m, 2H, Tetrahydropyran-CH_2_). ^13^C NMR (151 MHz, DMSO-*d*_6_) *δ* 168.0, 163.1, 156.4, 149.2, 146.2, 144.7, 139.2, 137.2, 135.6, 135.3, 132.6, 131.6, 129.3, 126.9 (2 × C), 122.3, 121.4, 115.3 (2 × C), 110.6, 108.8, 67.4, 67.1, 44.4, 35.2, 30.8, 8.7. ESI-HRMS *m/z*: calcd for C_27_H_28_O_4_N_4_^+^ [M + H]^+^, 473.2183; found, 473.2185. HPLC: *t*_R_ 5 min, purity 97.55%.

***N-((1H-pyrazol-3-yl)methyl)-2-(4-(5-methyl-4-(quinolin-6-yloxy)-1H-pyrazol-3-yl)phenoxy)acetamide (9l).*** ^1^H NMR (600 MHz, DMSO-*d*_6_) *δ* 8.76 (dd, *J* = 4.2, 1.8 Hz, 1H, Quinoline-CH), 8.45 (s, 1H, Pyrazole-CH), 8.23 (dd, *J* = 8.4, 1.8 Hz, 1H, Quinoline-CH), 8.03 (d, *J* = 9.0 Hz, 1H, Quinoline-CH), 7.74–7.58 (m, 3H, Ether-Ph-CH, Quinoline-CH), 7.44 (dd, *J* = 8.4, 4.2 Hz, 1H, Quinoline-CH), 7.20 (d, *J* = 3.0 Hz, 1H, Quinoline-CH), 6.93 (s, 2H, Ether-Ph-CH), 6.05 (d, *J* = 1.8 Hz, 1H, Pyrazole-CH), 4.49 (s, 2H, Ether-CH_2_), 4.30 (d, *J* = 6.0 Hz, 2H, Amine-CH_2_), 2.00 (s, 3H, Pyrazole-CH_3_). ^13^C NMR (151 MHz, DMSO-*d*_6_) *δ* 170.8, 167.9, 158.2, 156.4, 144.7, 141.5, 137.2, 135.6, 135.3, 132.6, 131.6, 129.3, 126.9 (2 × C), 124.5, 122.3, 121.4, 115.8 (2 × C), 108.8, 103.5, 67.3, 60.2 8.7. ESI-HRMS *m/z*: calcd for C_25_H_22_O_3_N_6_^+^ [M + H]^+^, 455.1826; found, 455.1829. HPLC: *t*_R_ 5 min, purity 98.83%.

### Biology

#### Cell cultures

Mouse microglial cell line BV2 cells (CL-0493, Wuhan Pricella Biotechnology Co., Ltd.) were cultured in high glucose-containing Dulbecco’s Modified Eagle Medium supplemented with 10% foetal bovine serum (C0232, GIBCO) in 95% humidified air and 5% CO_2_ at 37 °C.

#### Cell viability assay

Cell viability was detected with the CCK-8 Cell Counting Kit (Vazyme, China), according to the manufacture’s instruction. Briefly, BV2 cells were treated with 29 compounds (10 μM) for 24 h. Then, 10 μl of CCK-8 solution was added to each well for 1–2 h. Finally, the absorbance was measured at 450 nm.

#### Quantitative real-time PCR (qRT-PCR)

BV2 cells were pre-treated with 22 compounds for 2 h, and then stimulated with LPS for 4 h. Total RNA was extracted from BV2 cells, and inversely transcribed into cDNA. Then, qRT-PCR was performed. The upstream and downstream primers, 2 × ChamQ Univegsal SYBG qPCR Master Mix, cDNA and ddH_2_O were mixed for qRT-PCR. The primer information was shown in Table 4.

**Table 4.** The primer information of qRT-PCR.

Genes		Sequence (5′ → 3′)
Glyceraldehyde-3-phosphate dehydrogenase	Forward	ACCCAGAAGACTGTGGATGG
	Reverse	TGCTGTAGCCAAATTCGTTG
IL-1β	Forward	TCGTGCTGTCGGACCCATAT
	Reverse	GTCGTTGCTTGGTTCTCCTTGT
IL-6	Forward	TACTCGGCAAACCTAGTGCG
	Reverse	GTGTCCCAACATTCATATTGTCAGT
TNF-α	Forward	CCCTCACACTCAGATCATCTTCT
	Reverse	GCTACGACGTGGGCTACAG
IL-10	Forward	GCTCTTACTGACTGGCATGAG
	Reverse	CGCAGCTCTAGGAGCATGTG
CD206	Forward	CTCTGTTCAGCTATTGGACGC
	Reverse	CGGAATTTCTGGGATTCAGCTTC
Nrf2	Forward	TCTTGGAGTAAGTCGAGAAGTGT
	Reverse	GTTGAAACTGAGCGAAAAAGGC
HO-1	Forward	AAGCCGAGAATGCTGAGTTCA
	Reverse	GCCGTGTAGATATGGTACAAGGA
SOD	Forward	CAGACCTGCCTTACGACTATGG
	Reverse	CTCGGTGGCGTTGAGATTGTT
NF-κB	Forward	ATGGCAGACGATGATCCCTAC
	Reverse	TGTTGACAGTGGTATTTCTGGTG
LPA2	Forward	TGCTACTACAACGAGACCATCG
	Reverse	ATGGCTGCAATAACCAGCAGA

#### Nitric oxide analysis

BV2 cells were treated with 10 μM compound for 2 h, and then stimulated with LPS for 24 h. Then, the nitric oxide test kit (s0021, Beyotime) was used and the expression of nitric oxide (NO) *in vitro*.

#### Immunofluorescence staining analysis

BV2 cells were treated with 10 μM compound for 12 h, and then stimulated with LPS for 4 h. BV2 cells were fixed with 4% paraformaldehyde, and immunofluorescence was performed. The primary antibody CD206 antibody (18704-1-AP, Proteintech), CD68 (65187-1-Ig, Proteintech) and Nrf2 (16396-1-AP, Proteintech) were used.

#### ROS staining analysis

BV2 cells were treated with 10 μM compound for 12 h, and then stimulated with LPS for 4 h. ROS experiment was performed according to the kit instruction (s0033, Beyotime). 2′,7′-Dichlorodihydrofluorescein diacetate was diluted in phosphate-buffered saline at 1:1000 and incubated at 37 °C for 20 min before being observed under a microscope.

#### Mitochondrial membrane potential detection

BV2 cells were administrated with **6g** for 2 h, and then stimulated with LPS for 4 h. The mitochondrial membrane potential was detected by using the enhanced mitochondrial membrane potential detection reagent kit (C2003, Beyotime).

#### Statistical analysis

Data were analysed using the GraphPad Prism 8.0 software (GraphPad Software Inc.). Differences between groups were analysed by the Student’s *t*-test or one-way analysis of variance/Bonferroni multiple-comparison *post hoc* test. Significant probability values are denoted as *P < 0.05.

## Supplementary Material

Supporting_Information_ Clean.docx

## Data Availability

The authors confirm that the data supporting the findings of this study are available within the article and the datasets presented in the current study are available from the corresponding author upon reasonable request.
