# Supplementary material for: Synthesis of novel pyrazole derivatives and neuroprotective effect investigation
Source: J Enzyme Inhib Med Chem. 2025 Nov 12;40(1):2583820. doi: 10.1080/14756366.2025.2583820 (PMC12613307; doi:10.1080/14756366.2025.2583820)
Supplement: Supporting_Information_ Clean.docx [file IENZ_A_2583820_SM8336.docx]

**Supporting Information**

**Synthesis of novel pyrazole derivatives and neuroprotective effect investigation**

**CONTENTS**

[2D NMR spectra of representative compounds S2](#_Toc204264036)

[^1^H and ^13^C NMR spectra of synthetic compounds S5](#_Toc204264037)

[HRMS spectra of target compounds S34](#_Toc204264038)

[HPLC spectra of target compounds S41](#_Toc204264039)

[Supplementary Tables S55](#_Toc204264040)

2D NMR spectra of representative compounds


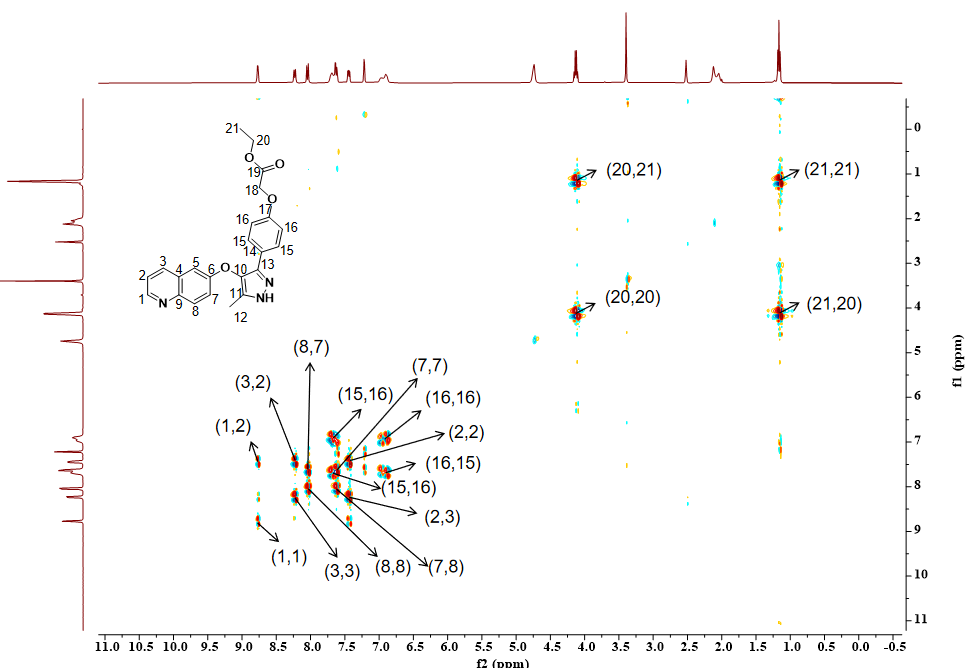


COSY spectra of compound **6g**


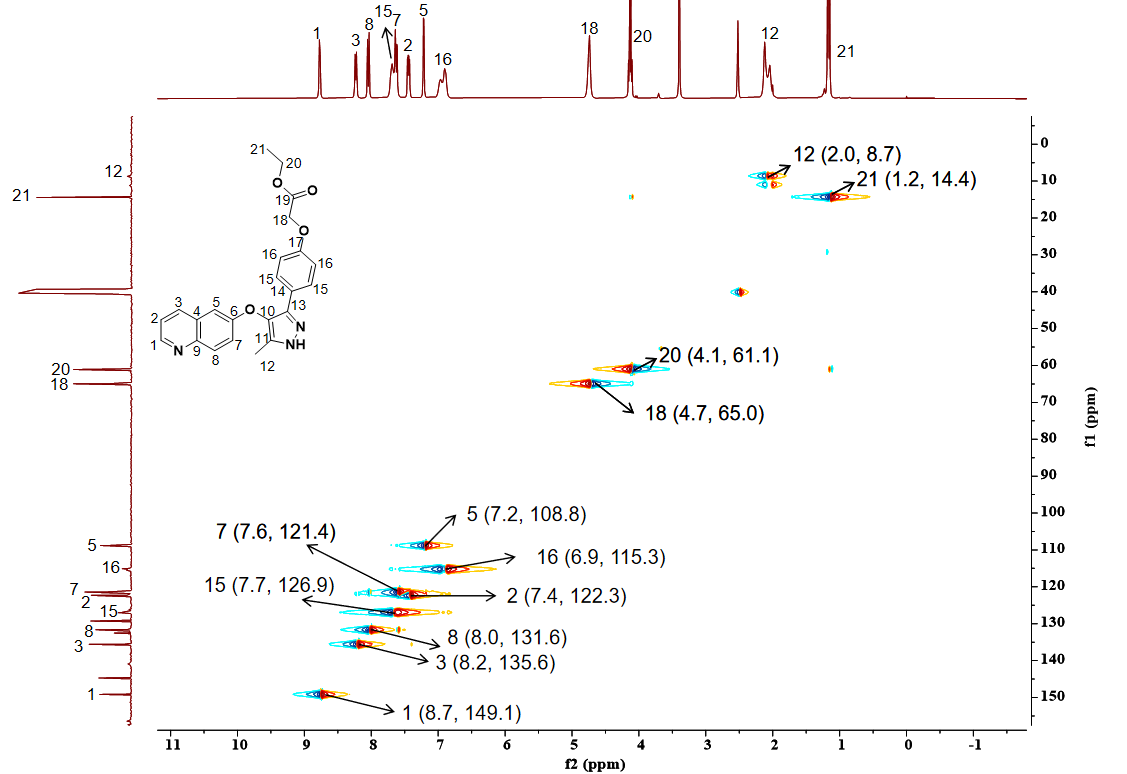


HSQC spectra of compound **6g**


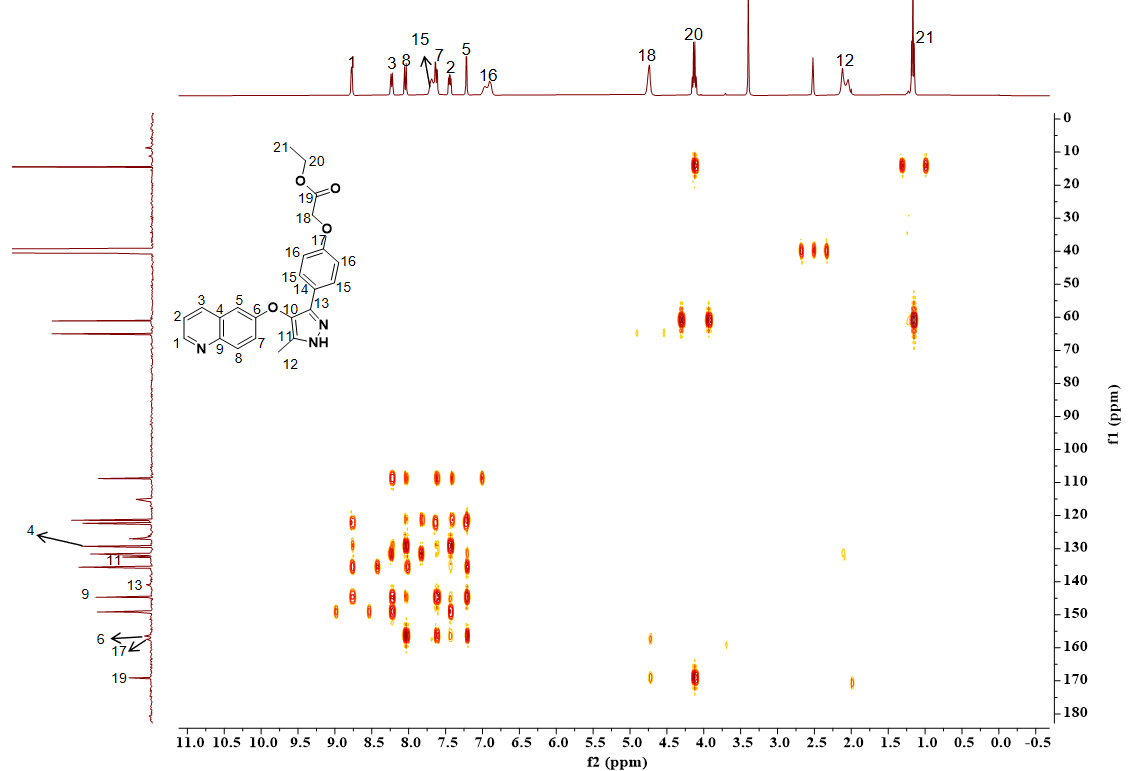


HMBC spectra of compound **6g**


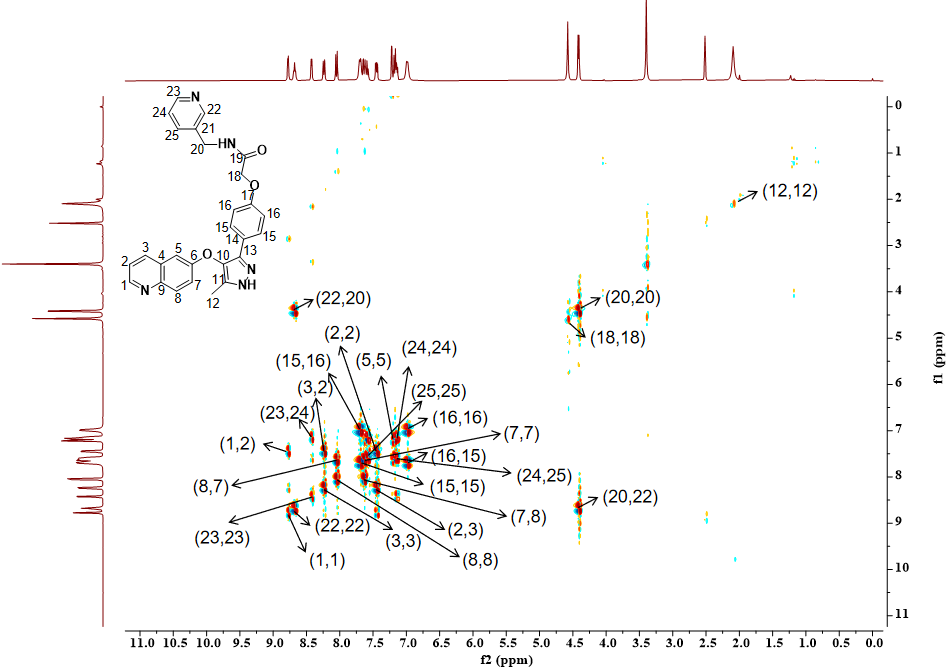


COSY spectra of compound **9h**


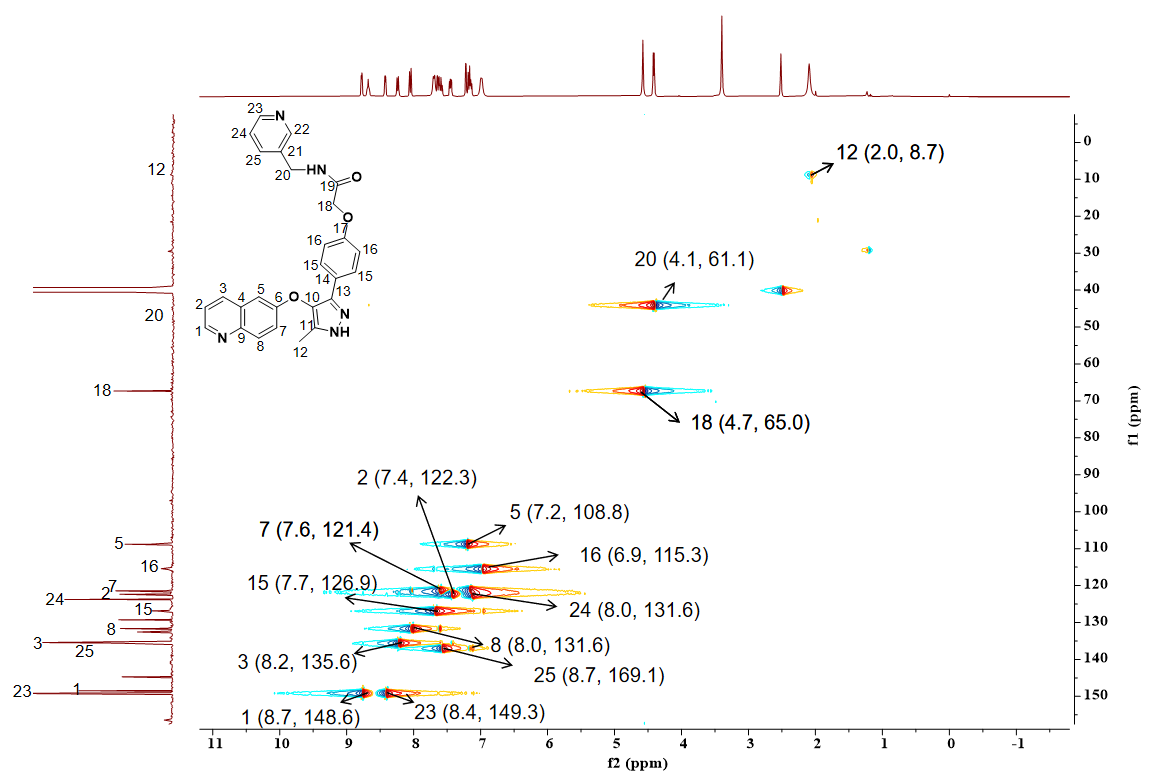


HSQC spectra of compound **9h**


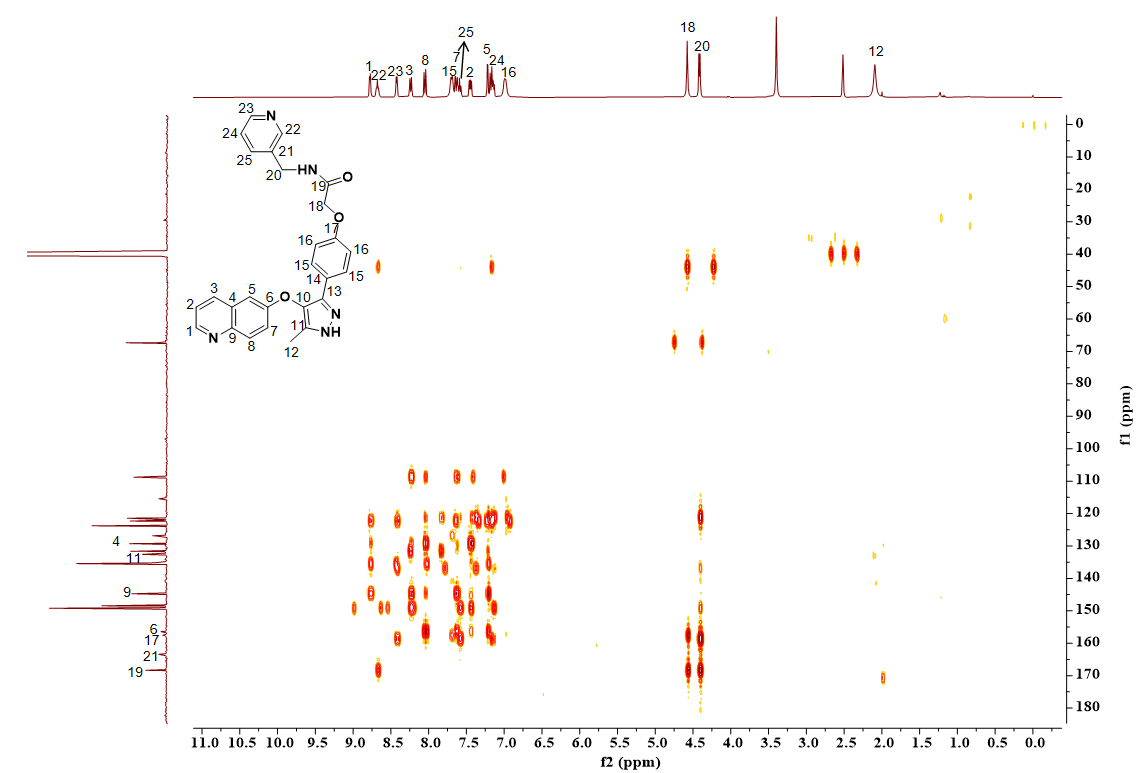


HMBC spectra of compound **9h**

^1^H and ^13^C NMR spectra of synthetic compounds

^1^H NMR


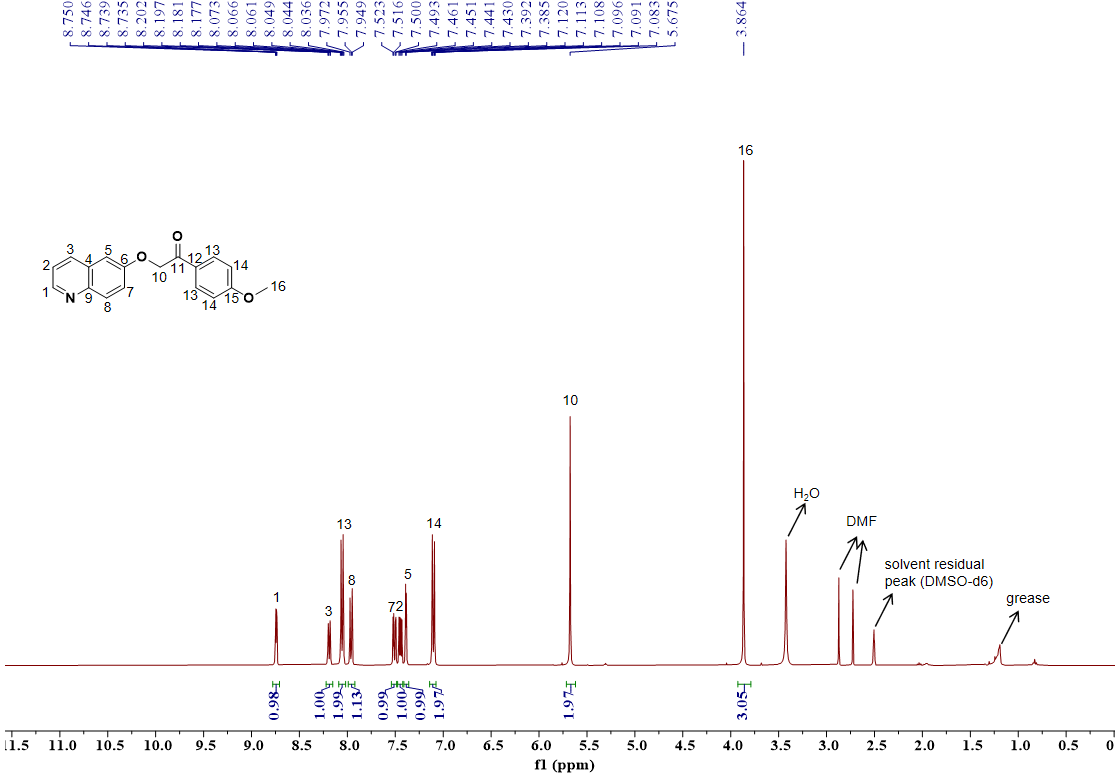


^1^H NMR spectra of compound **2**


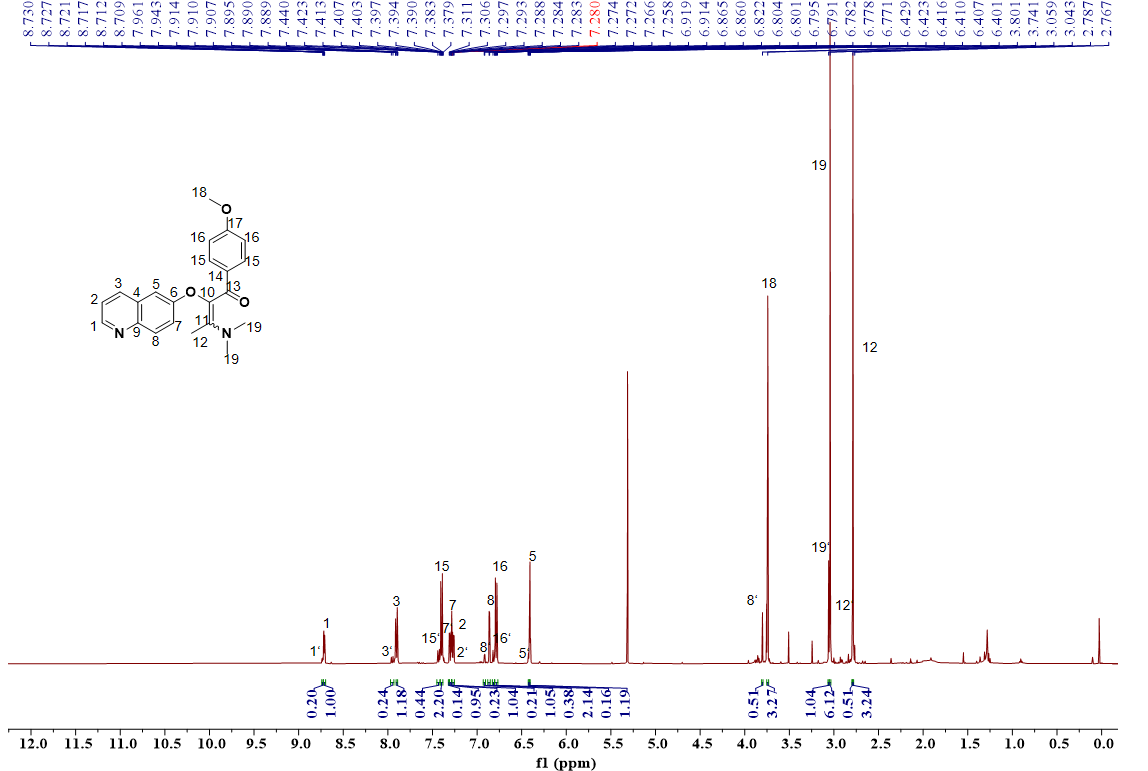


^1^H NMR spectra of compound **3**

^
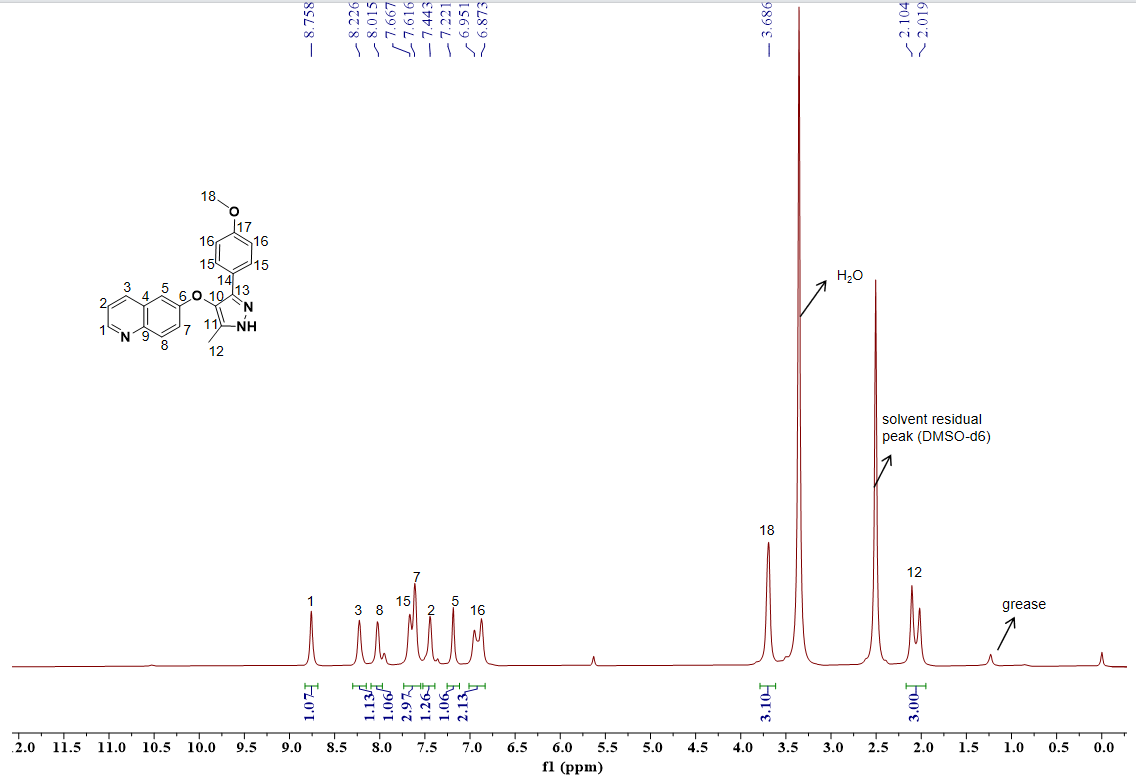
1^H NMR spectra of compound **4**

^
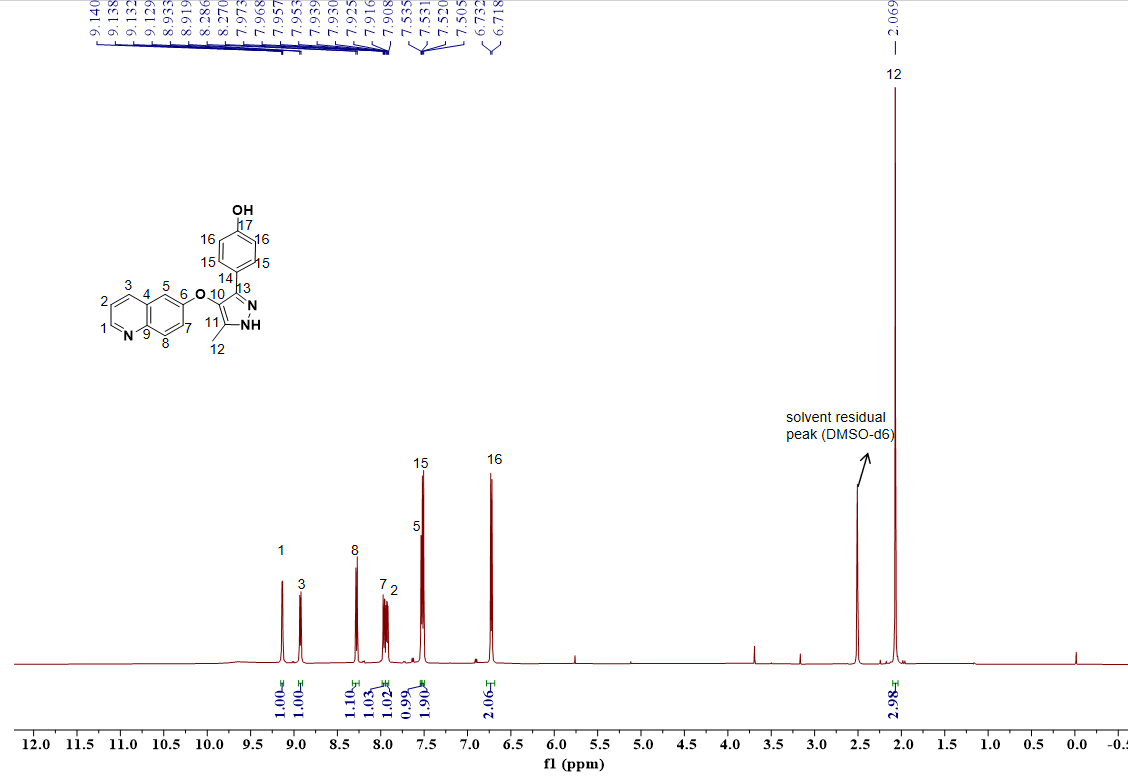
1^H NMR spectra of compound **5**


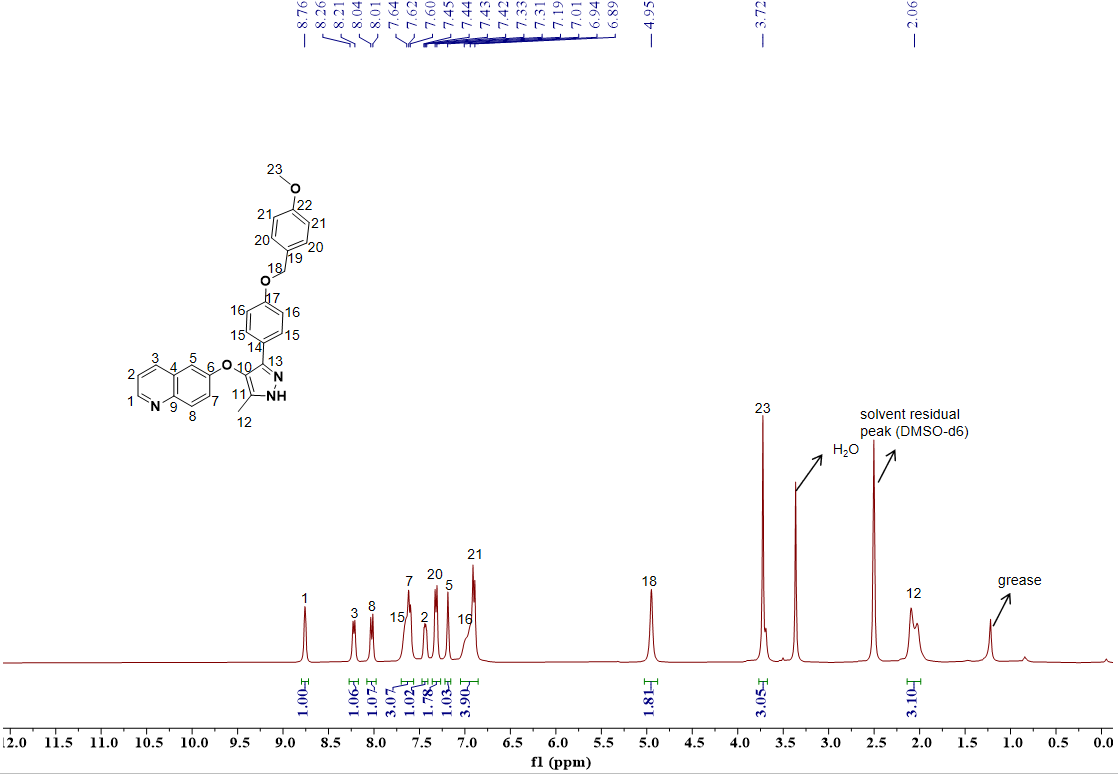


^1^H NMR spectra of compound **6a**

**
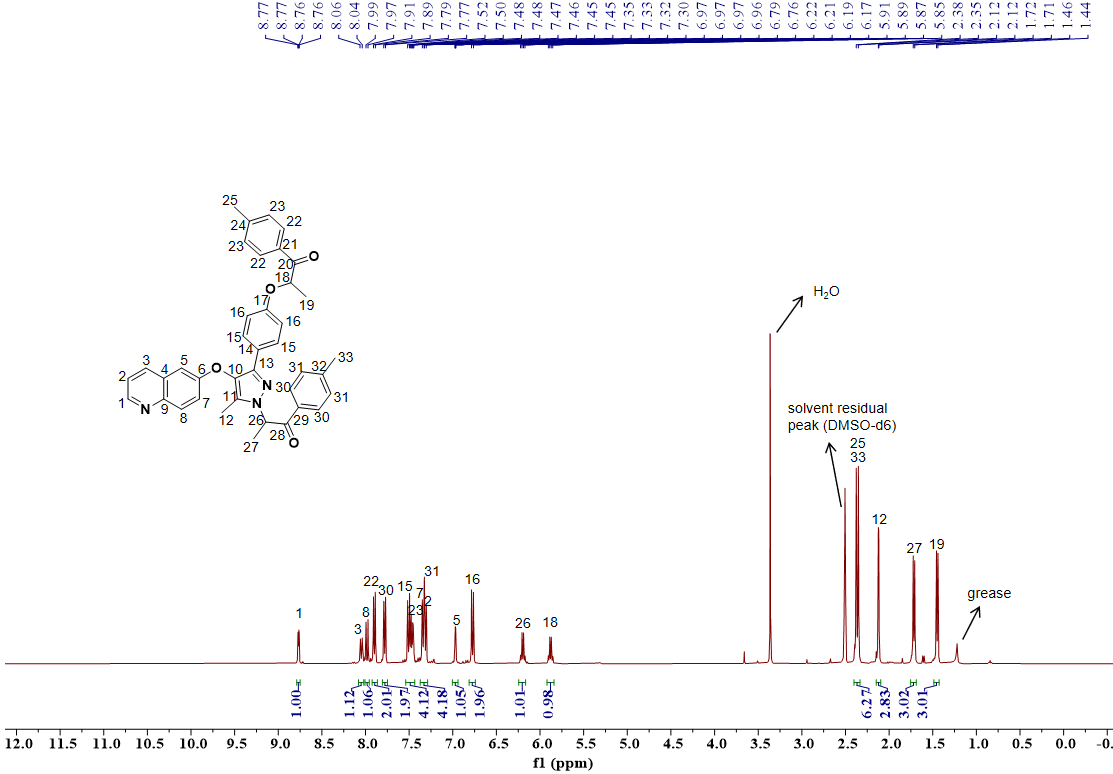
**

^1^H NMR spectra of compound **6b**

**
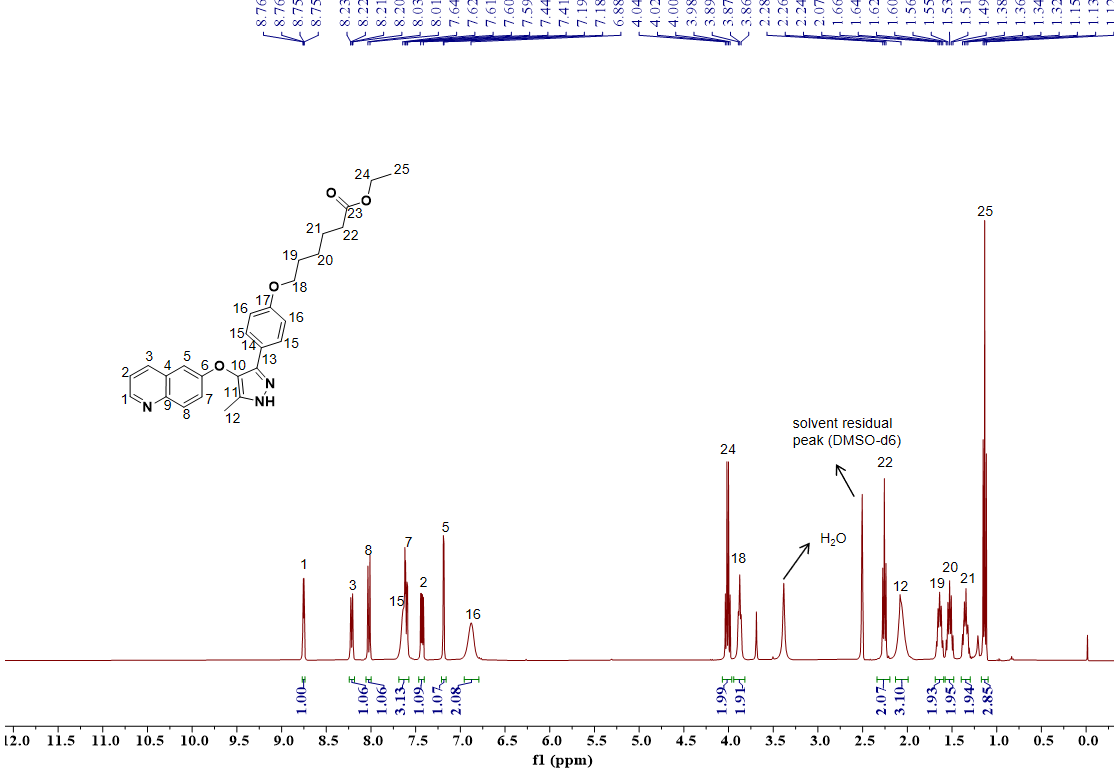
**

^1^H NMR spectra of compound **6c**

**
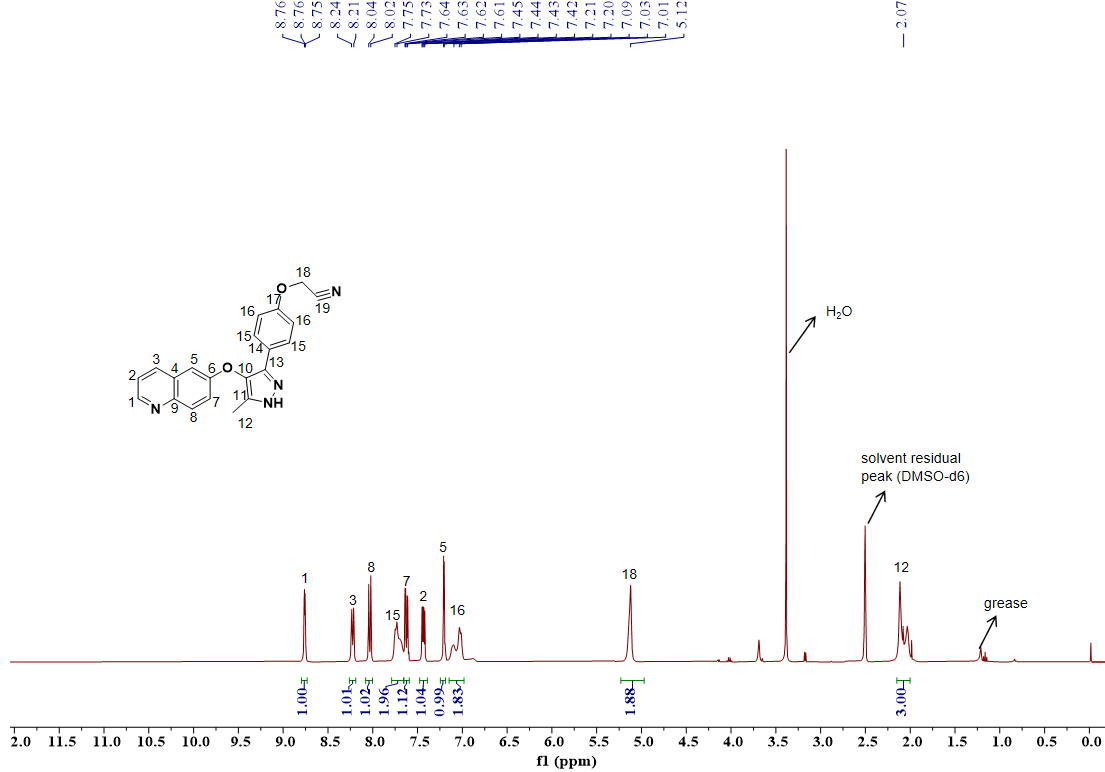
**

^1^H NMR spectra of compound **6d
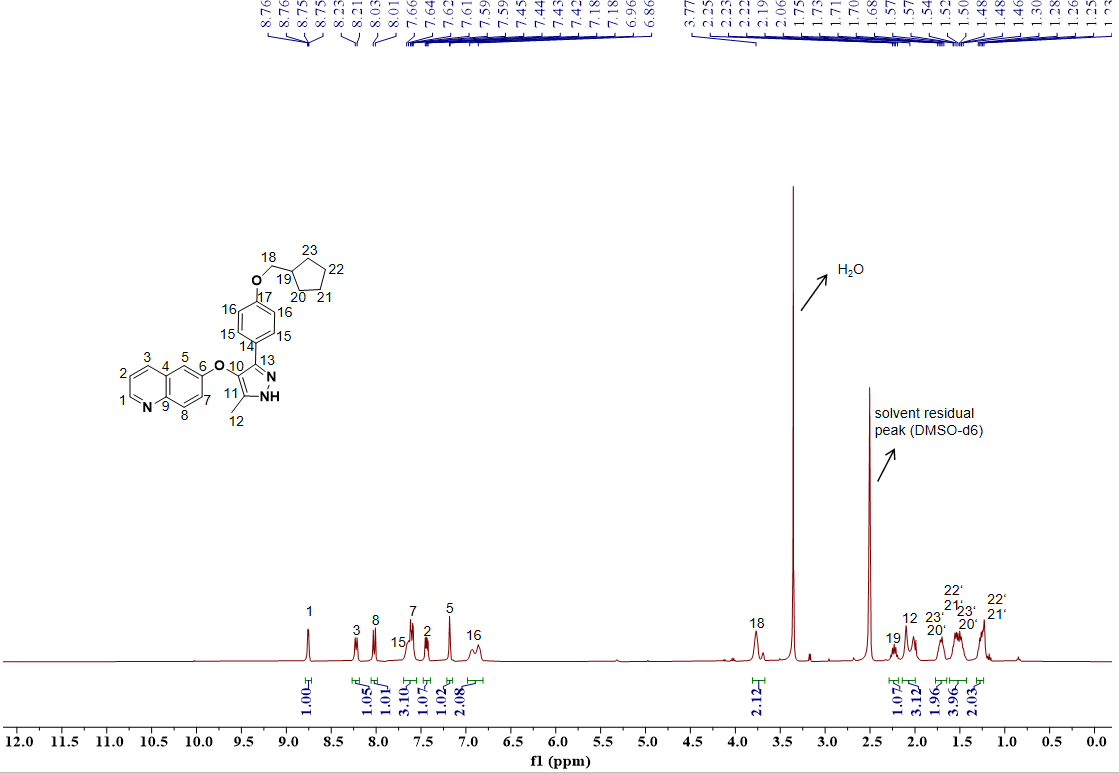
**

^1^H NMR spectra of compound **6e**

**
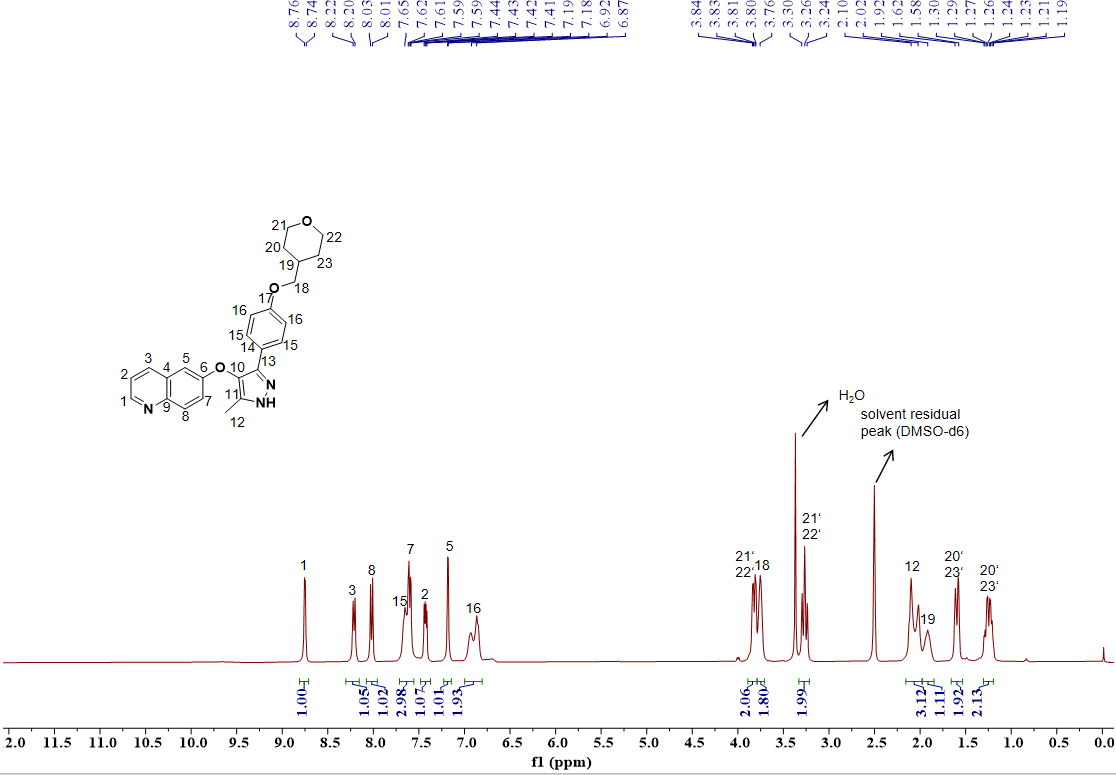
**

^1^H NMR spectra of compound **6f**

^
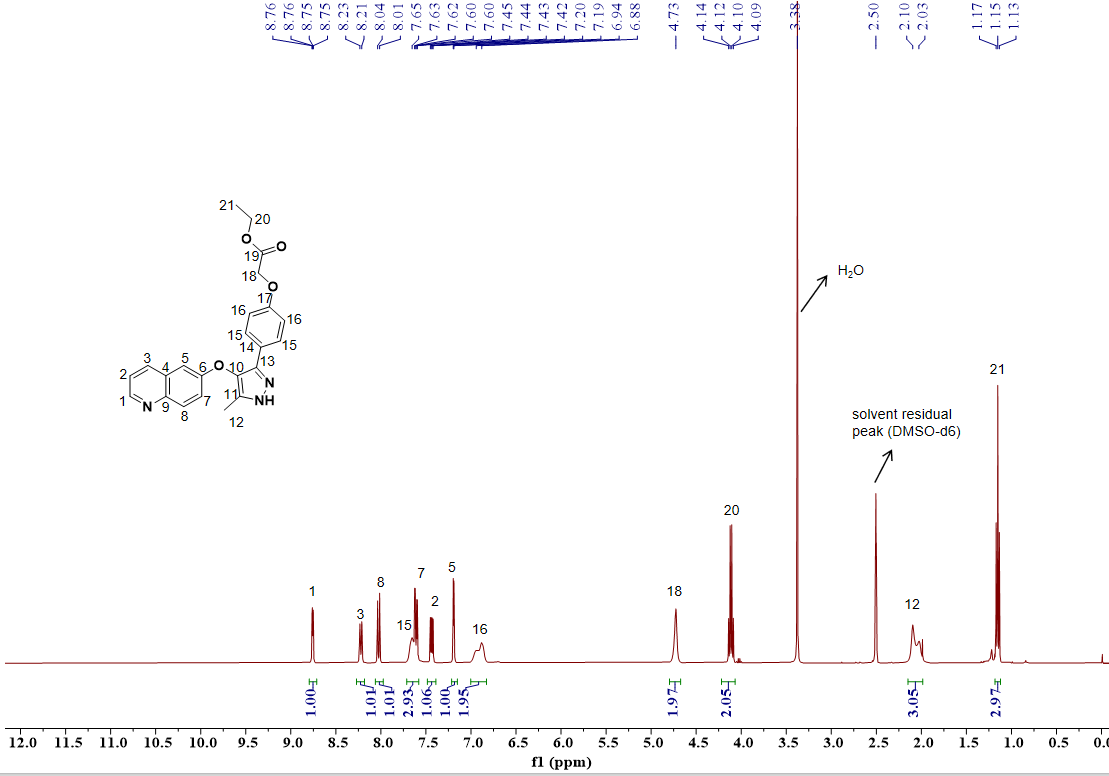
1^H NMR spectra of compound **6g**


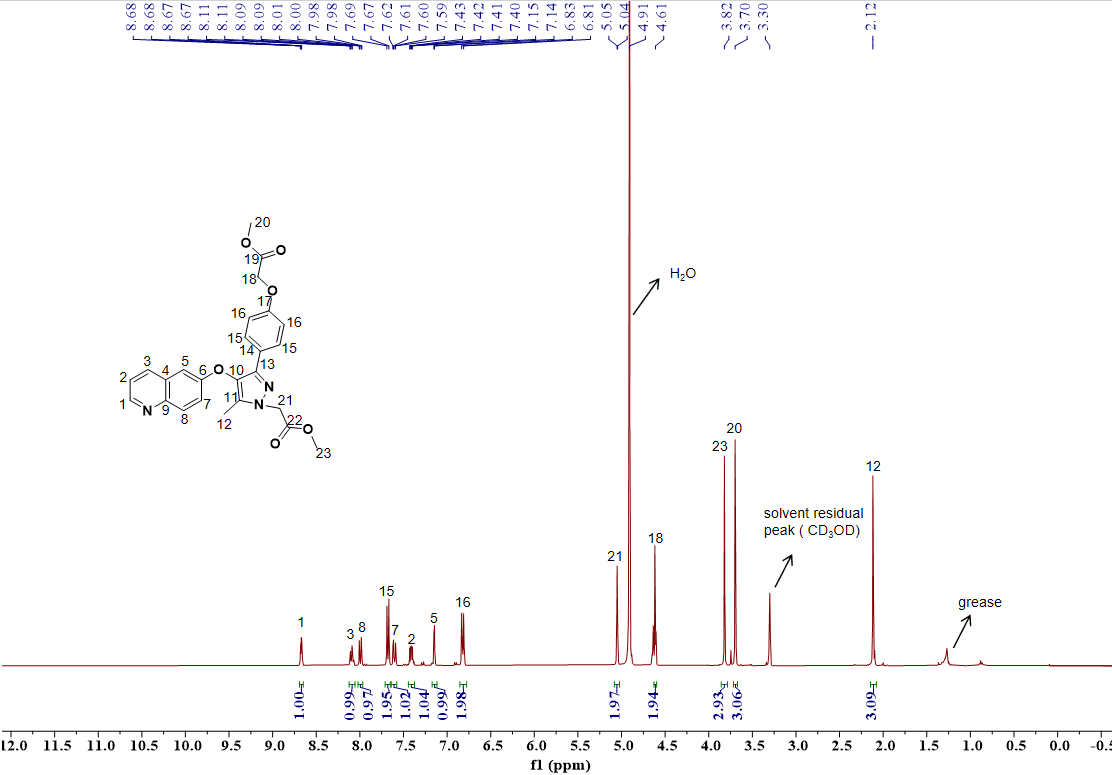


^1^H NMR spectra of compound **6h**


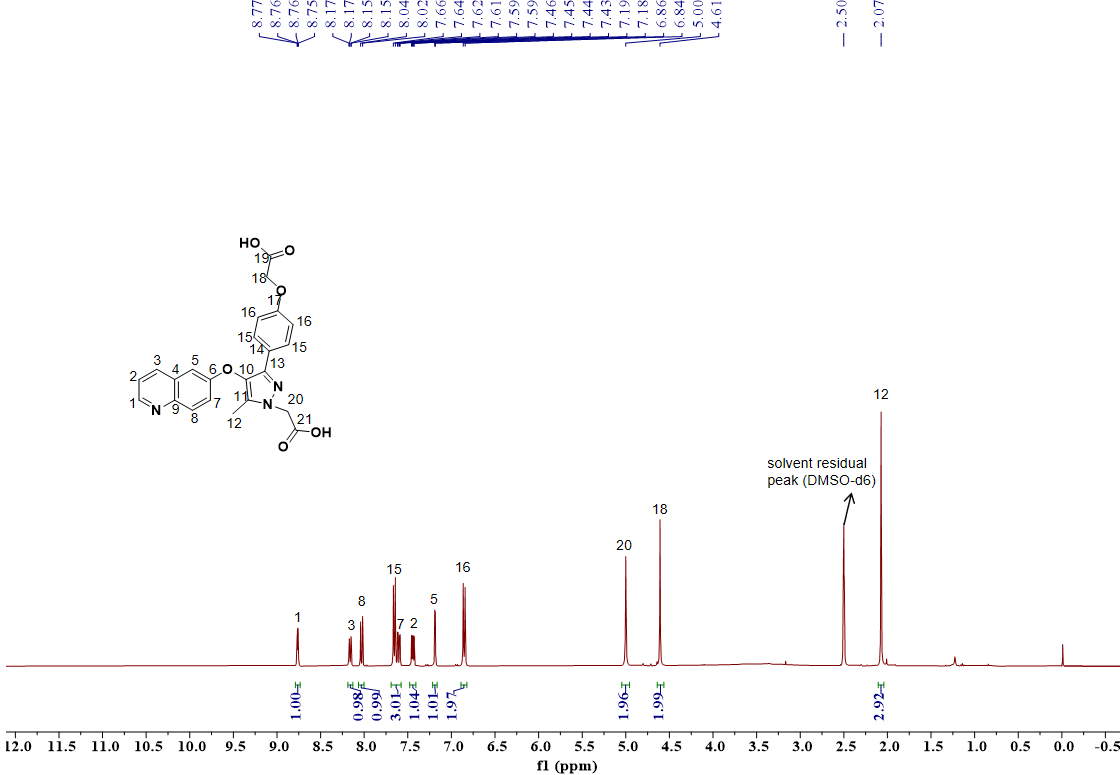


^1^H NMR spectra of compound **6i**


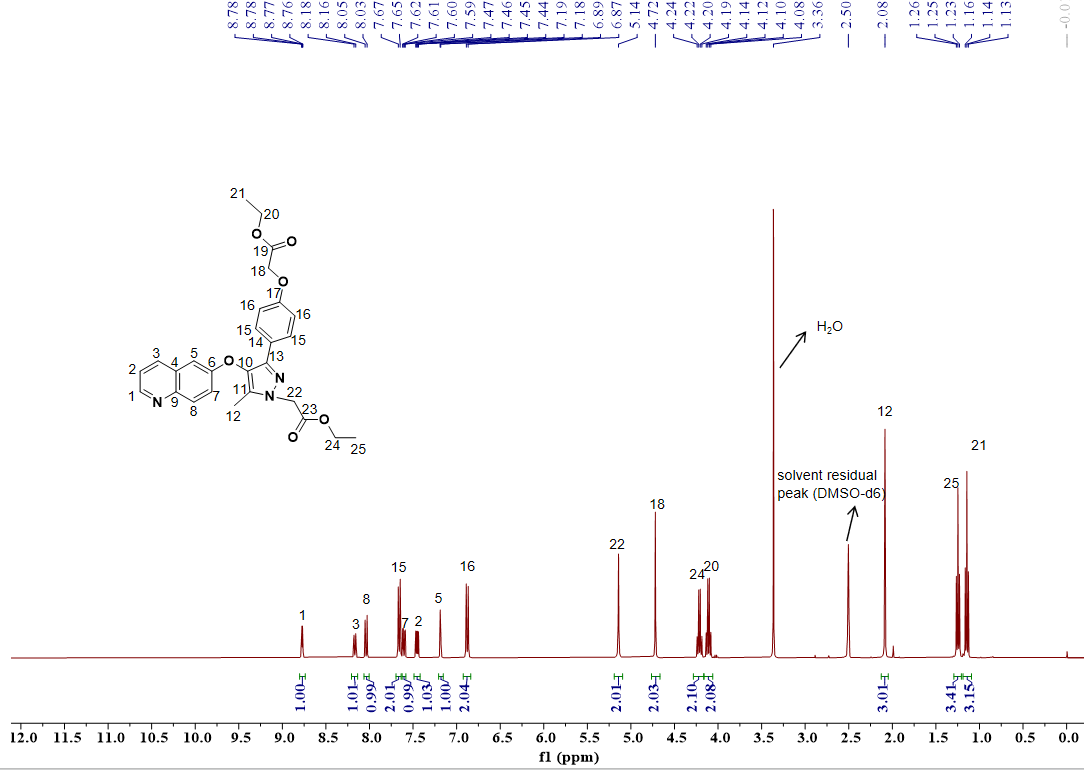


^1^H NMR spectra of compound **6j**


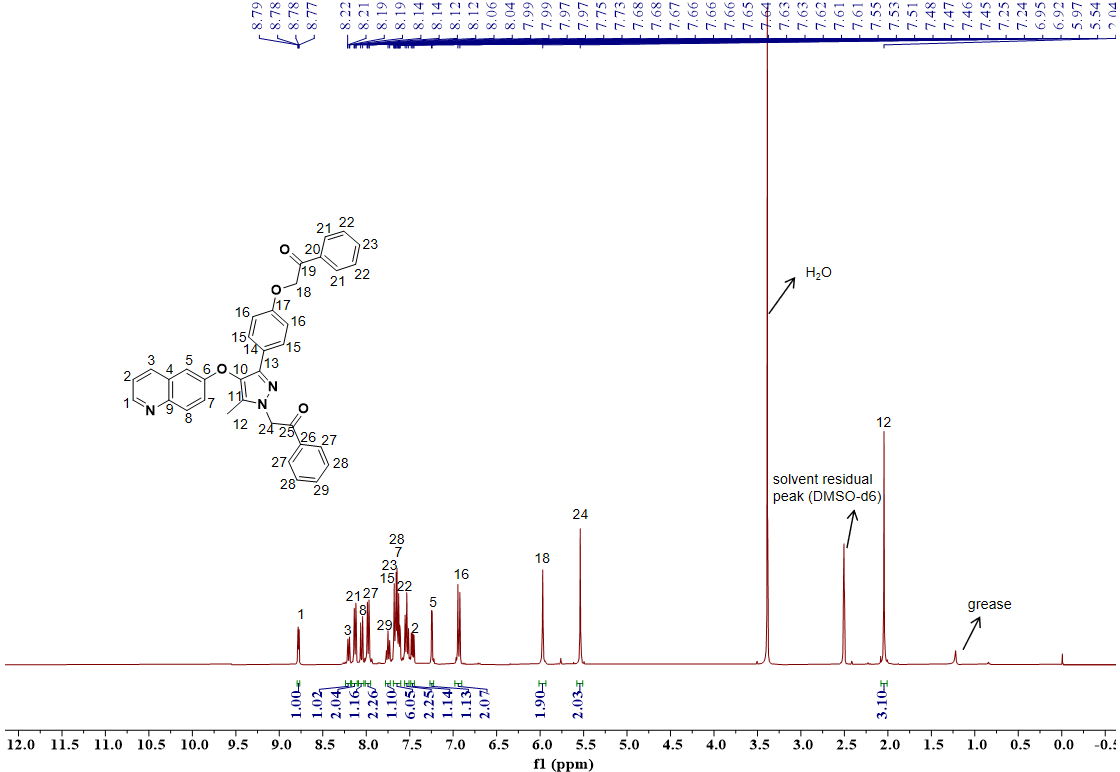


^1^H NMR spectra of compound **6k**


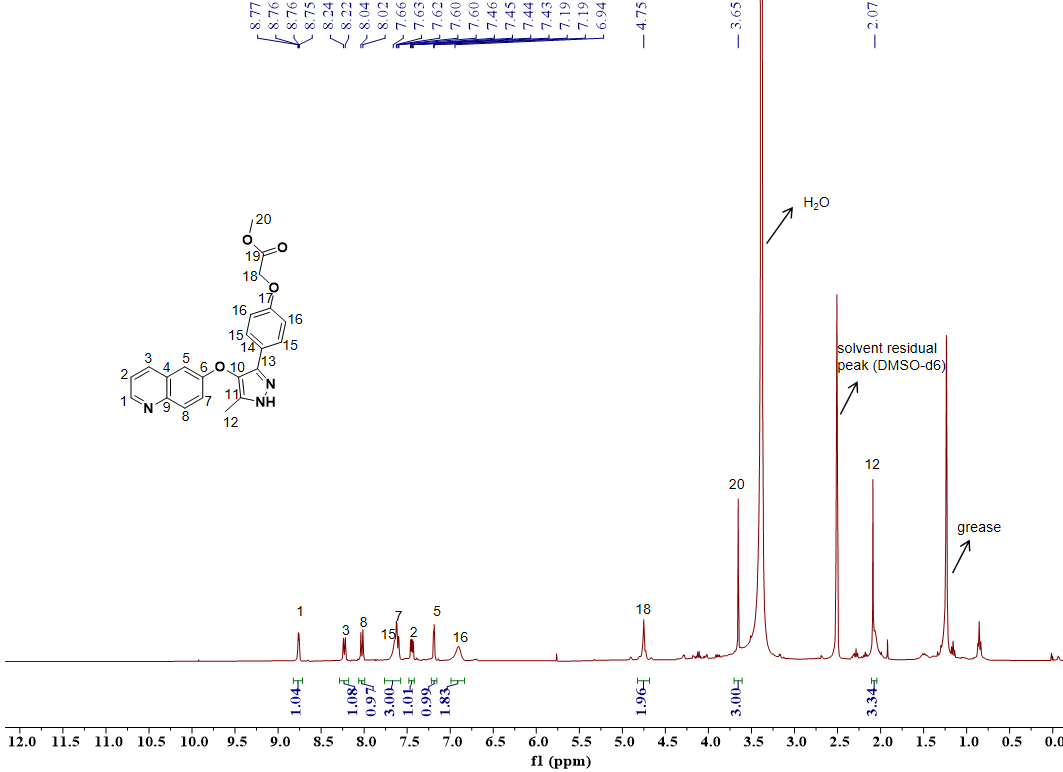


^1^H NMR spectra of compound **7**


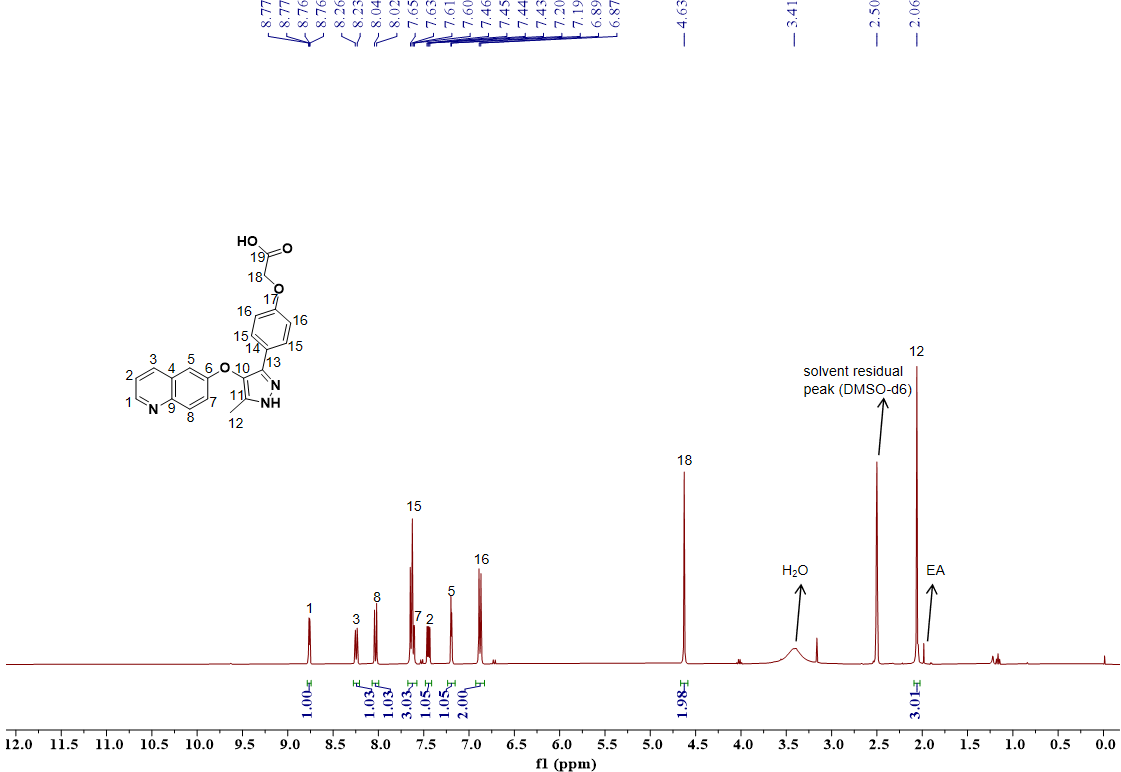


^1^H NMR spectra of compound **8**


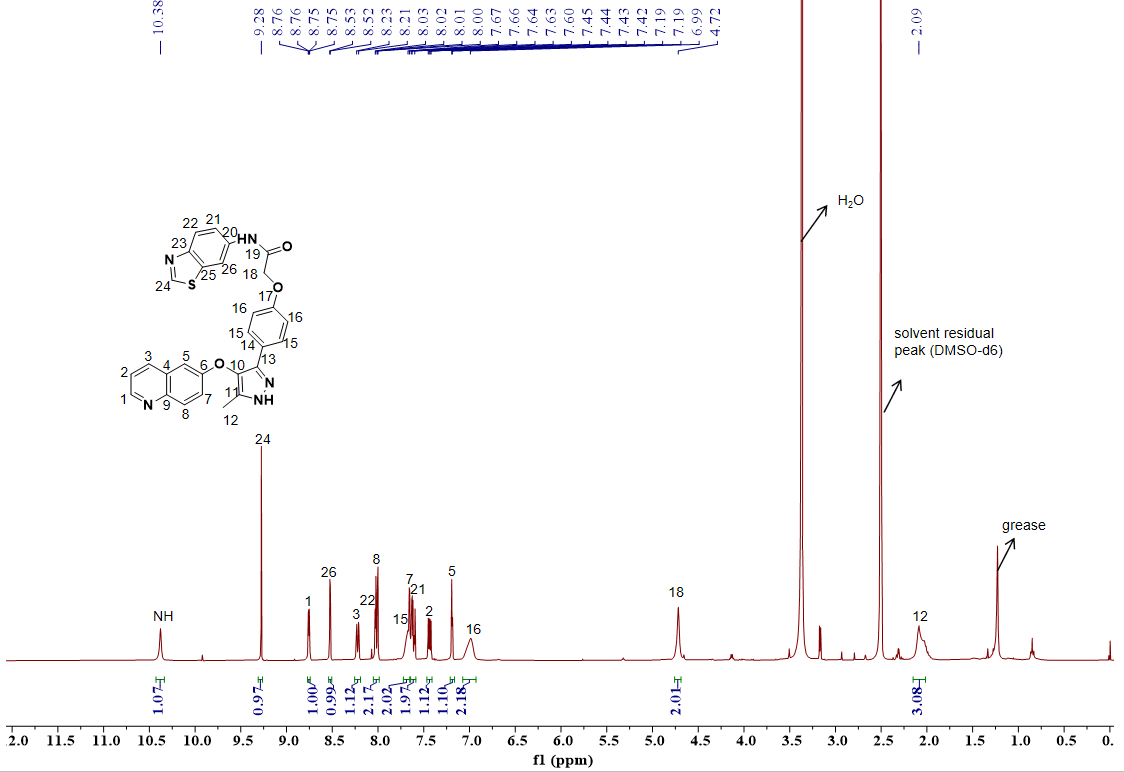


^1^H NMR spectra of compound **9a**


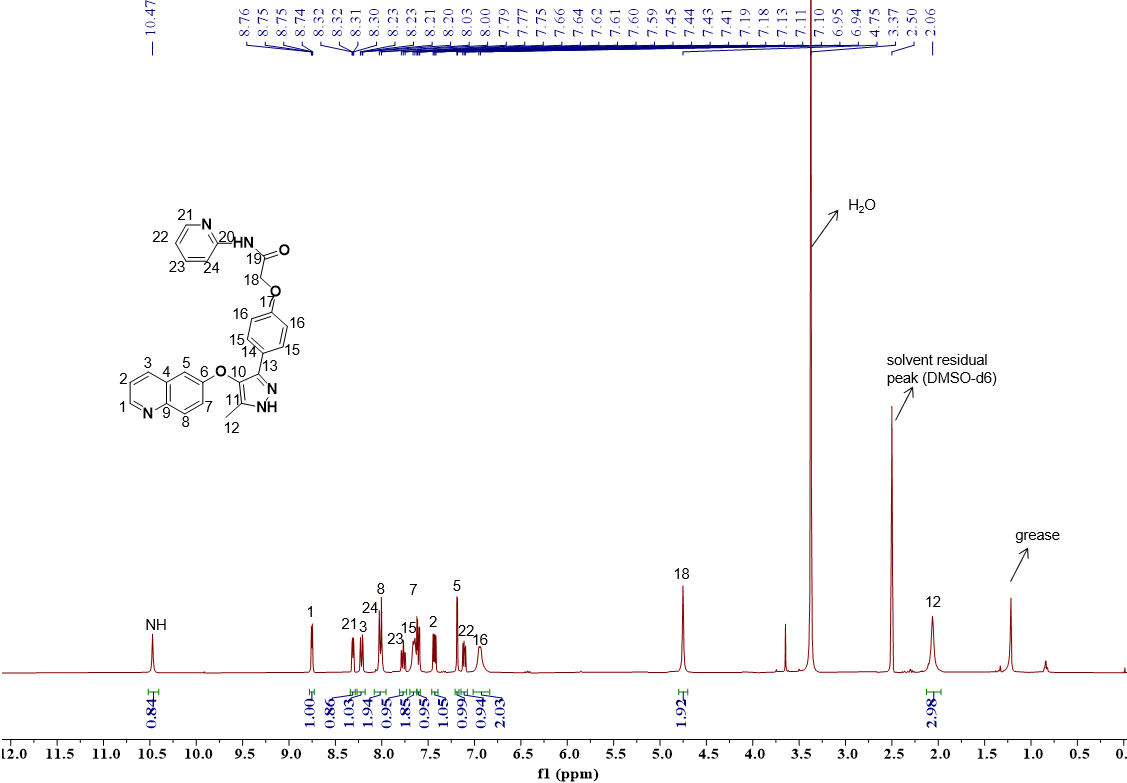


^1^H NMR spectra of compound **9b**


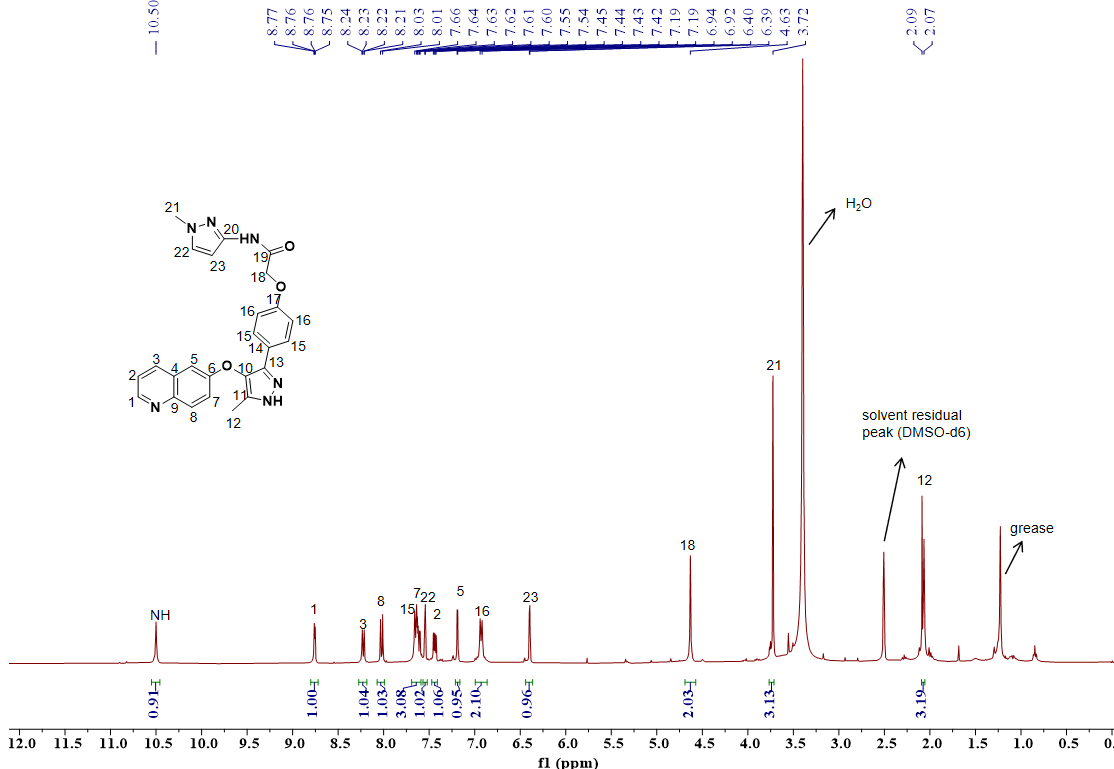


^1^H NMR spectra of compound **9c**


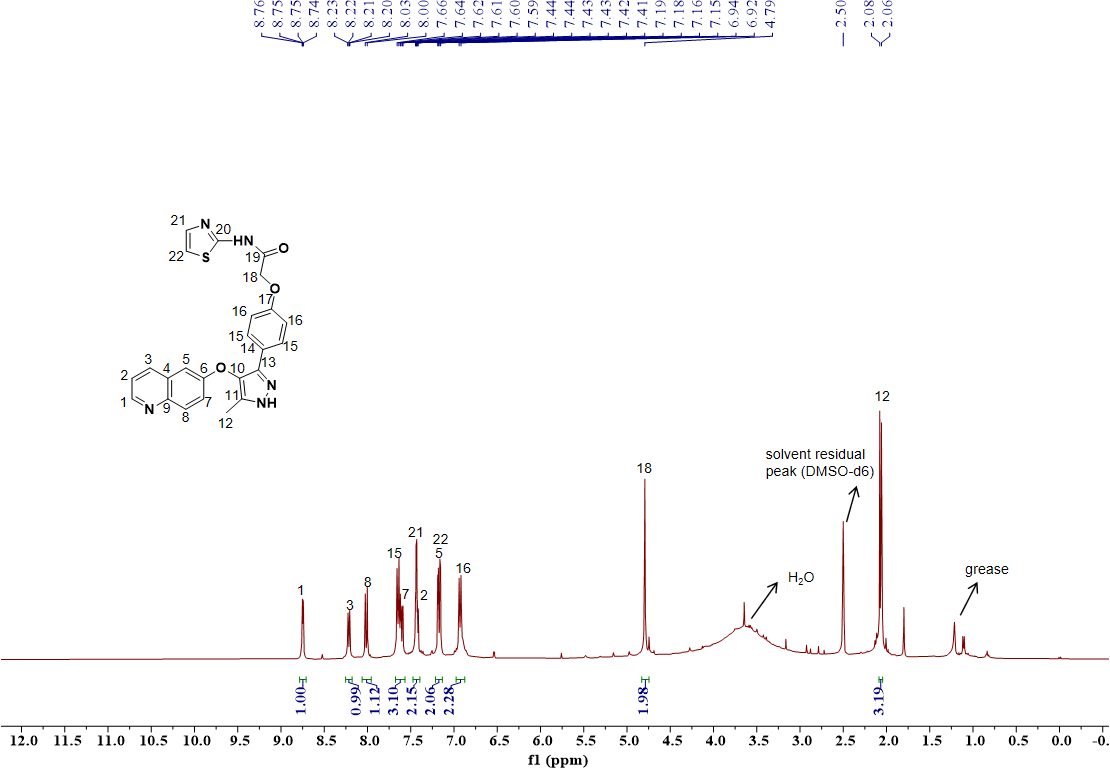


^1^H NMR spectra of compound **9d**


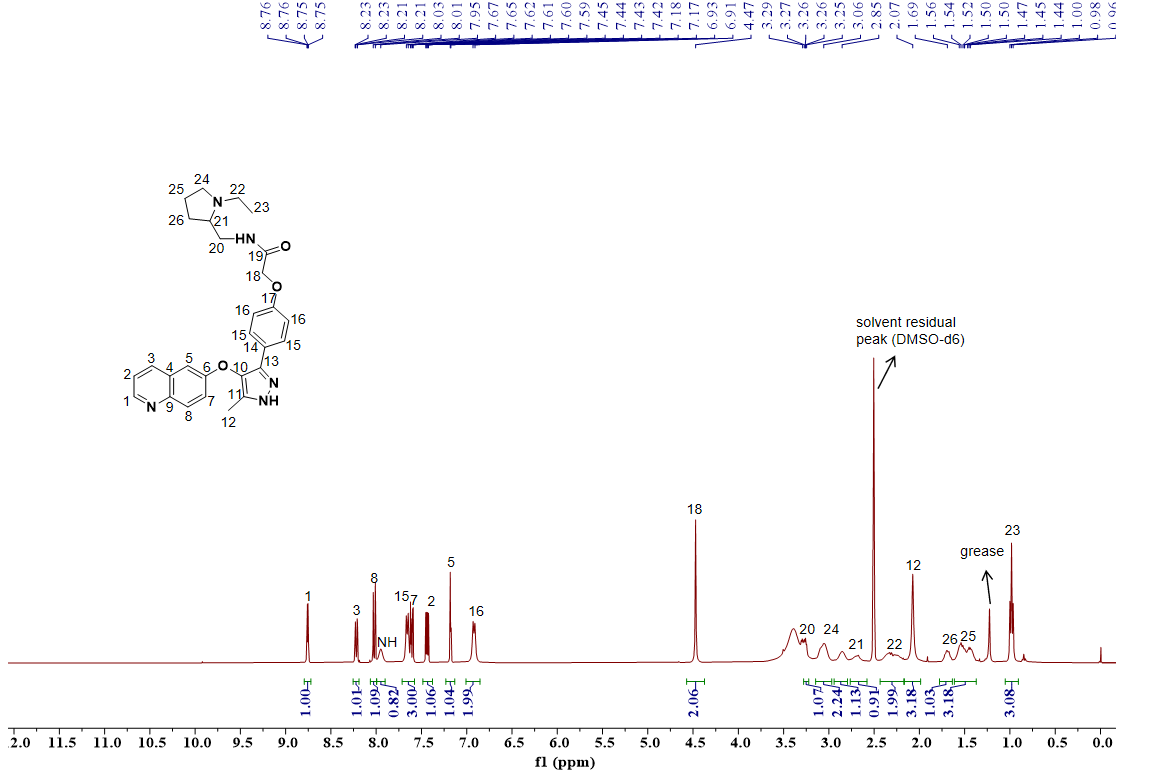


^1^H NMR spectra of compound **9e**


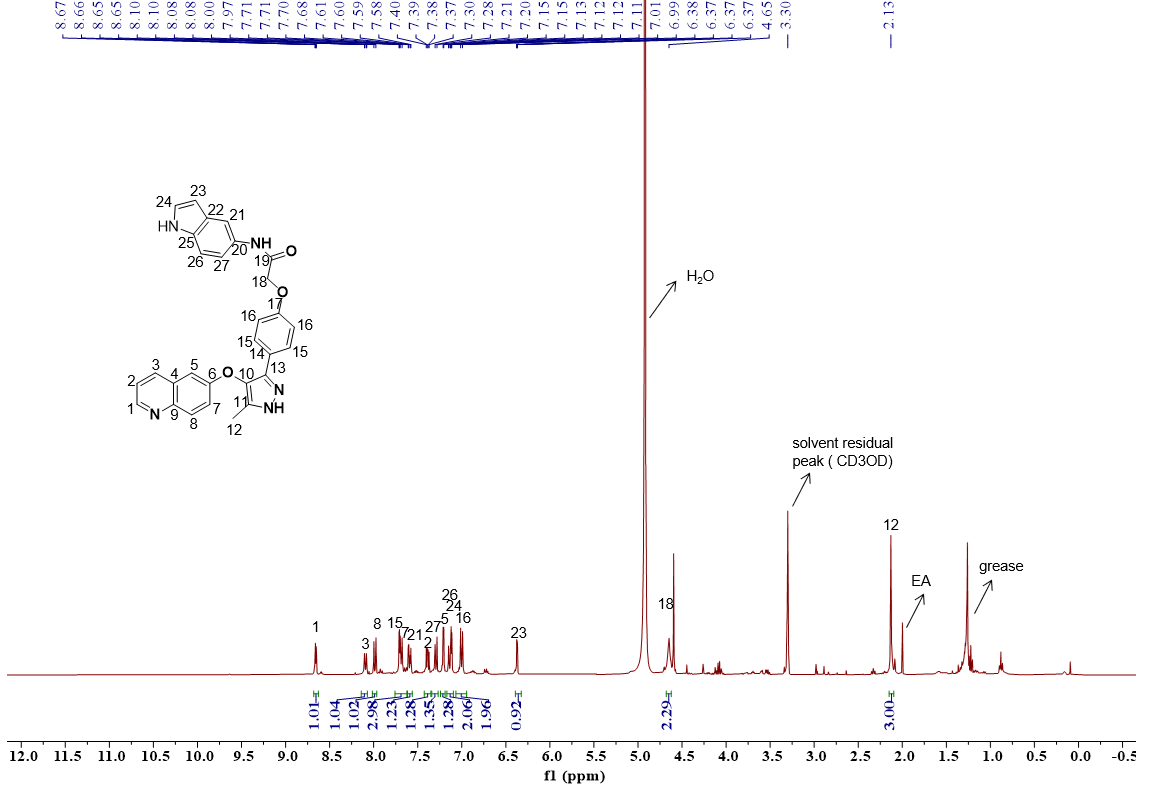


^1^H NMR spectra of compound **9f**


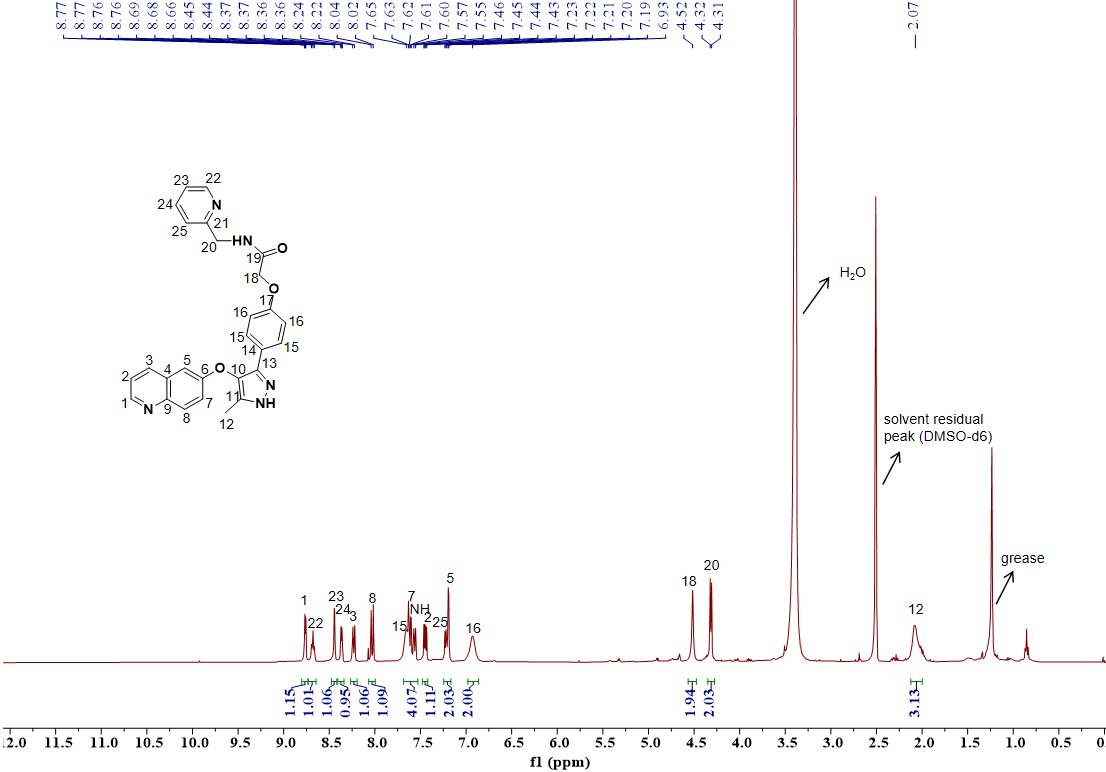


^1^H NMR spectra of compound **9g**


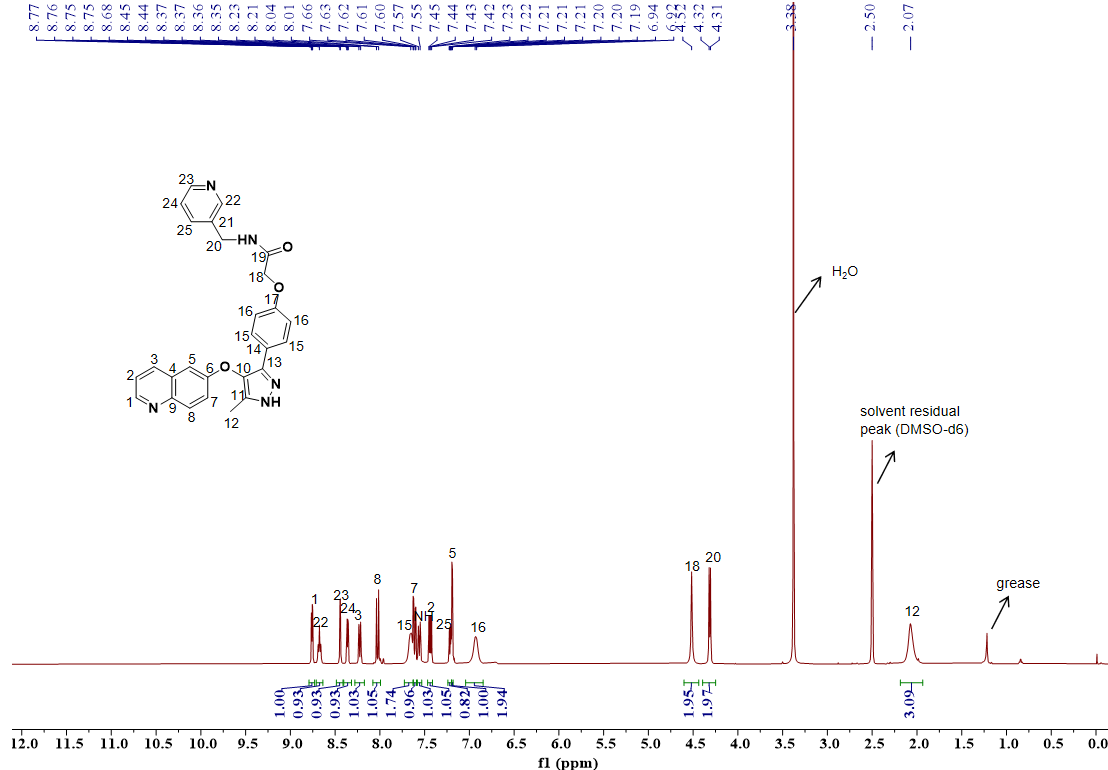


^1^H NMR spectra of compound **9h**

**
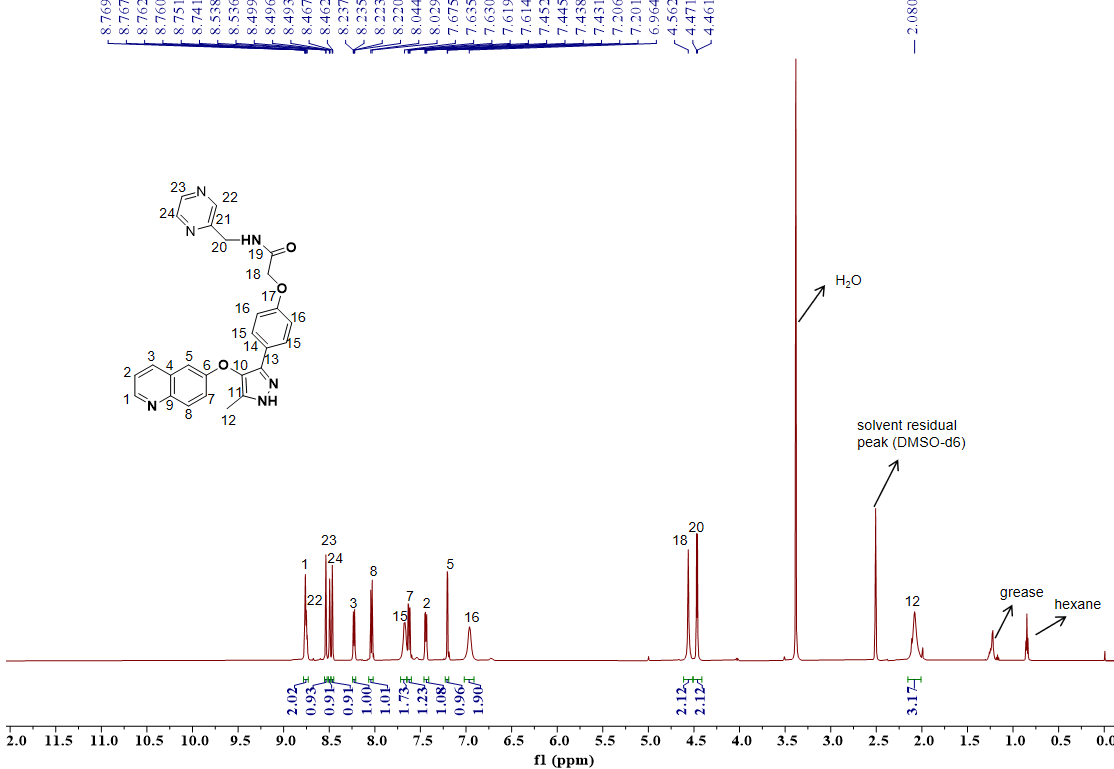
**

^1^H NMR spectra of compound **9i**


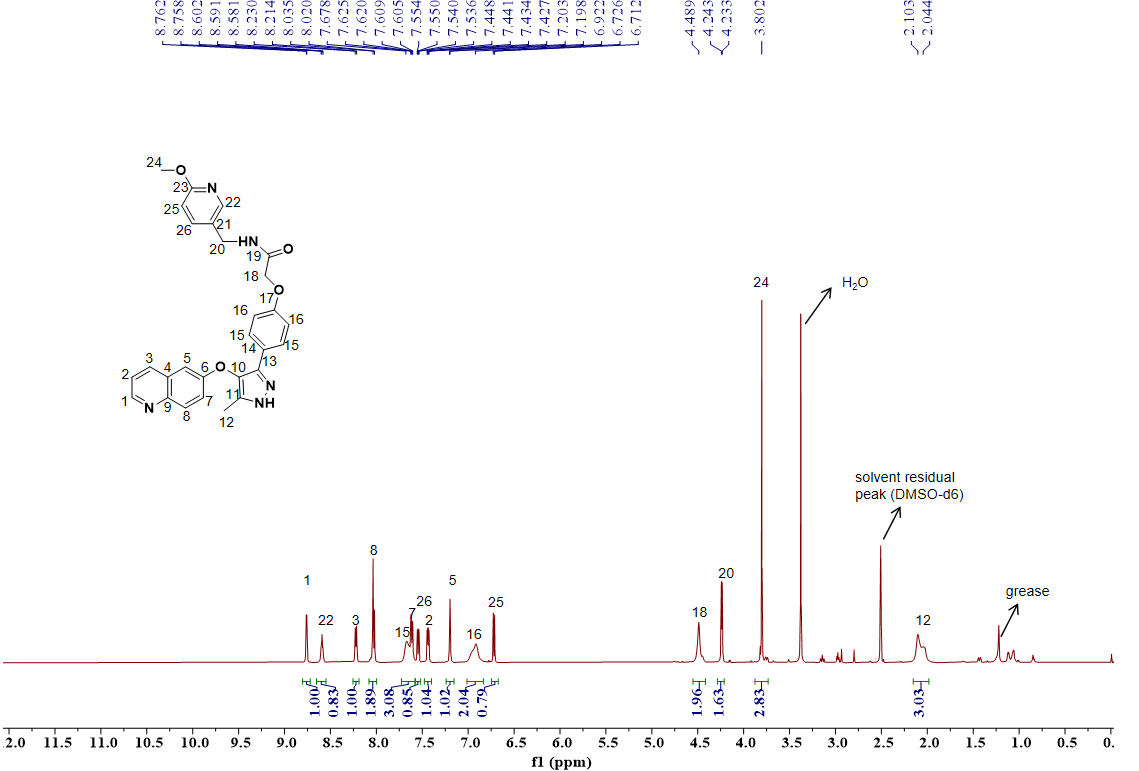


^1^H NMR spectra of compound **9j**


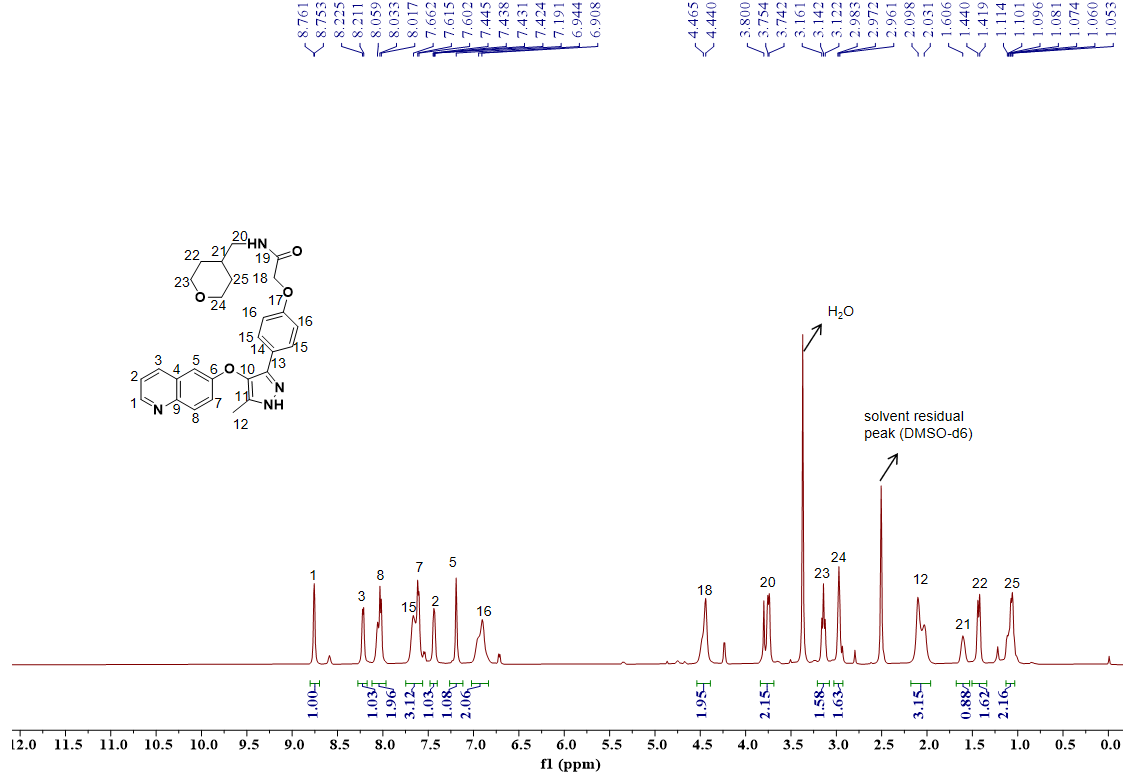


^1^H NMR spectra of compound **9k**


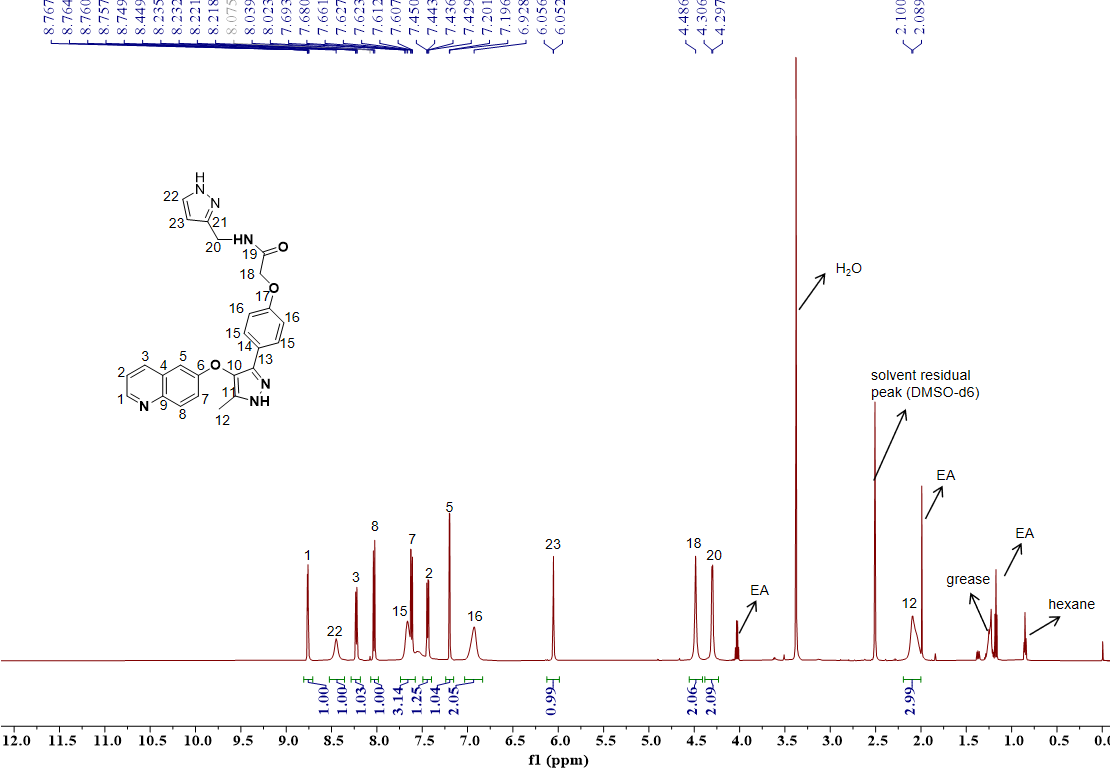


^1^H NMR spectra of compound **9l**

^13^C NMR


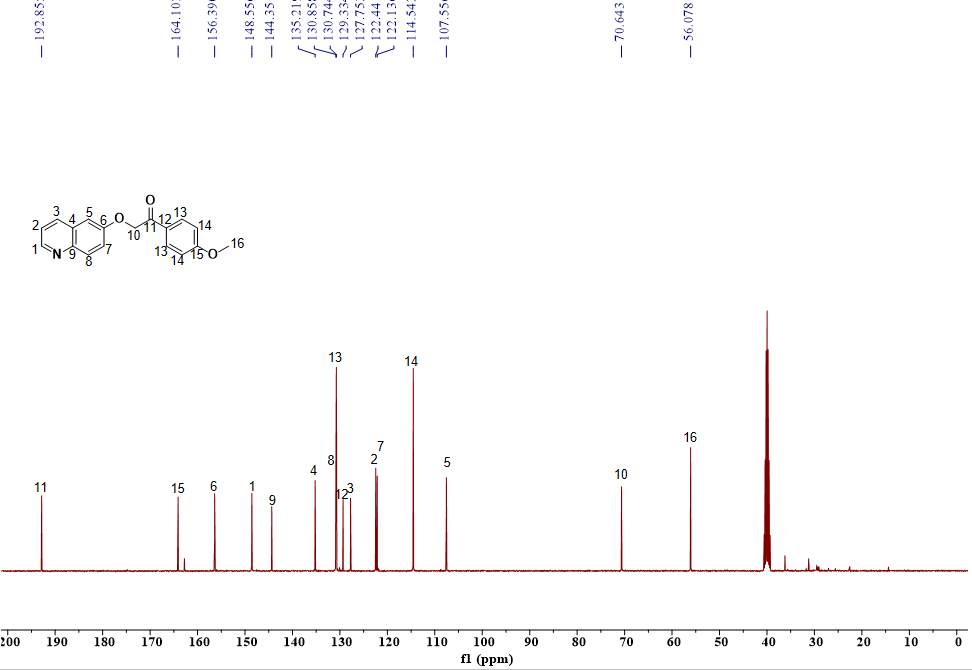


^13^C NMR spectra of compound **2**


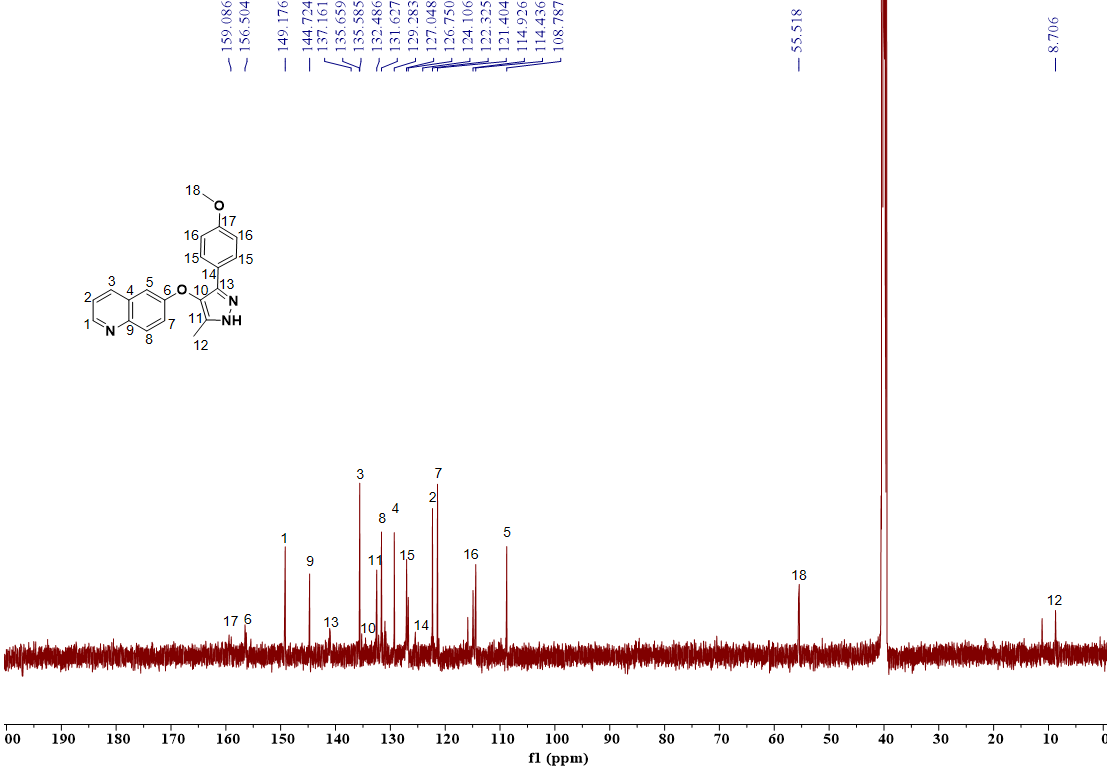


^13^C NMR spectra of compound **4**


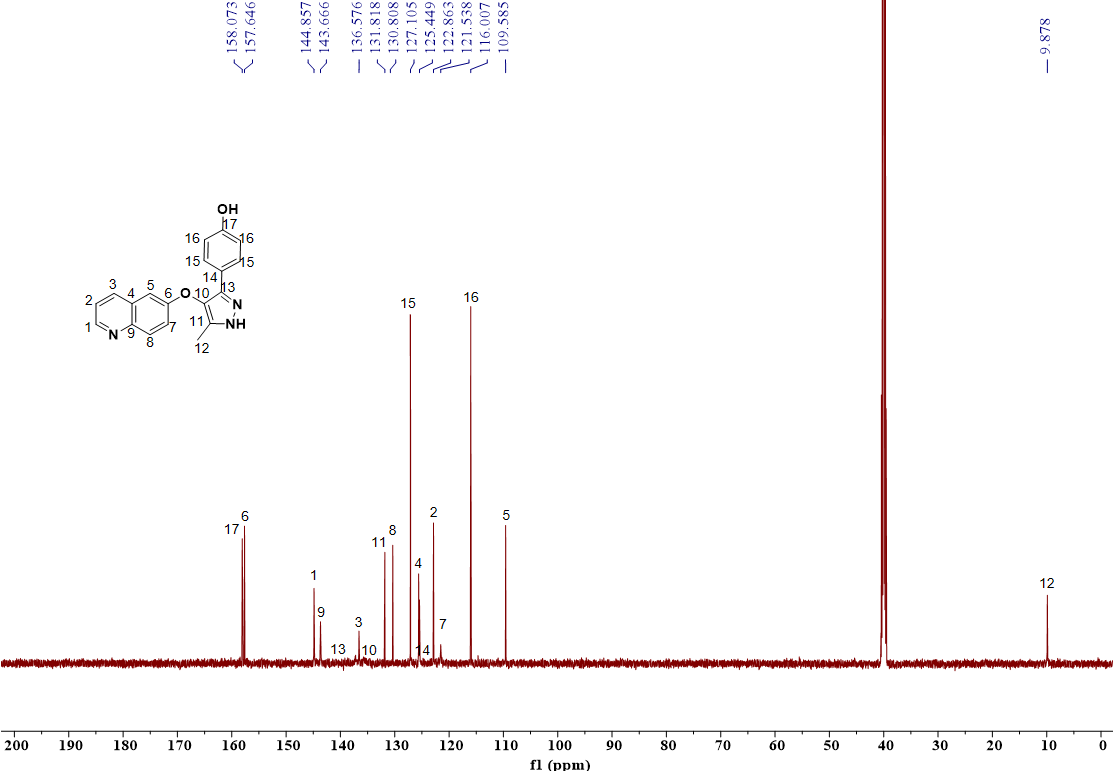


^13^C NMR spectra of compound **5**


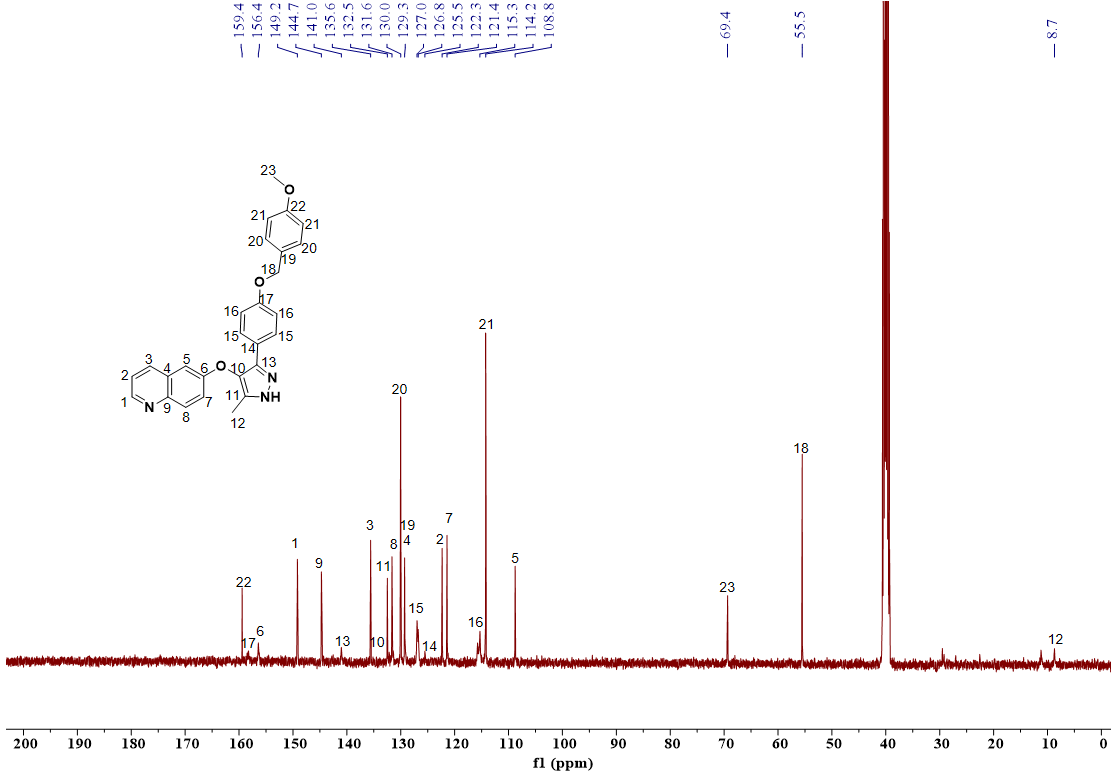


^13^C NMR spectra of compound **6a**


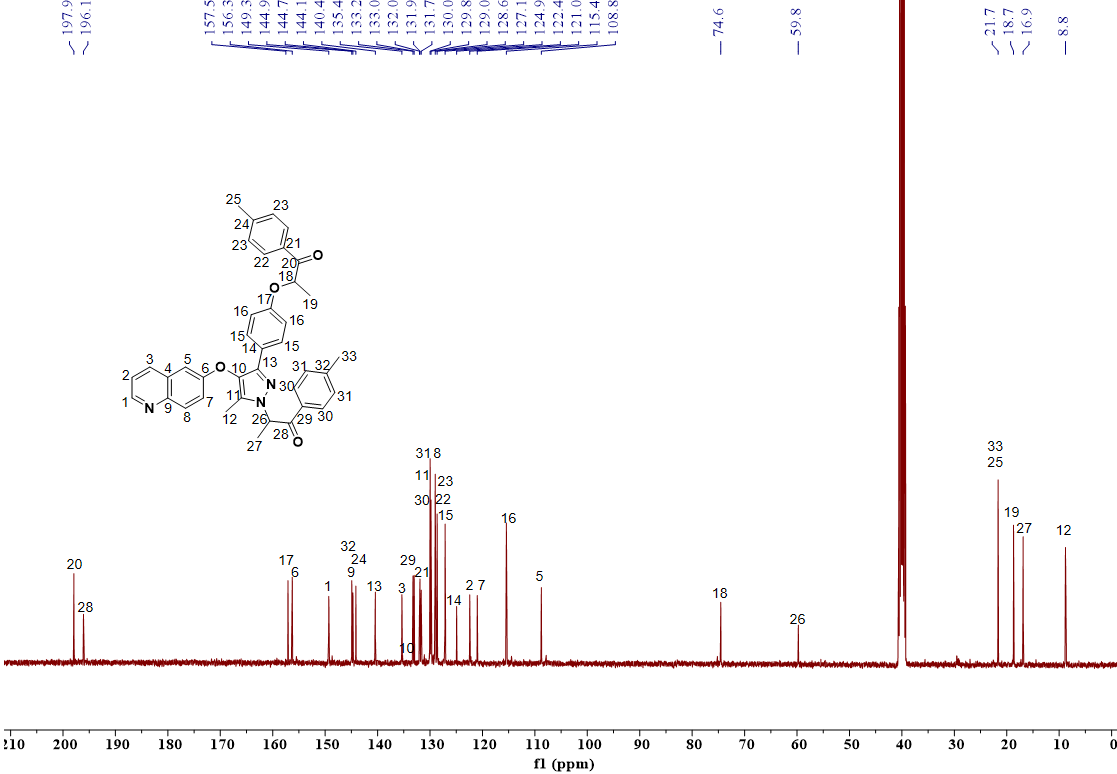


^13^C NMR spectra of compound **6b**


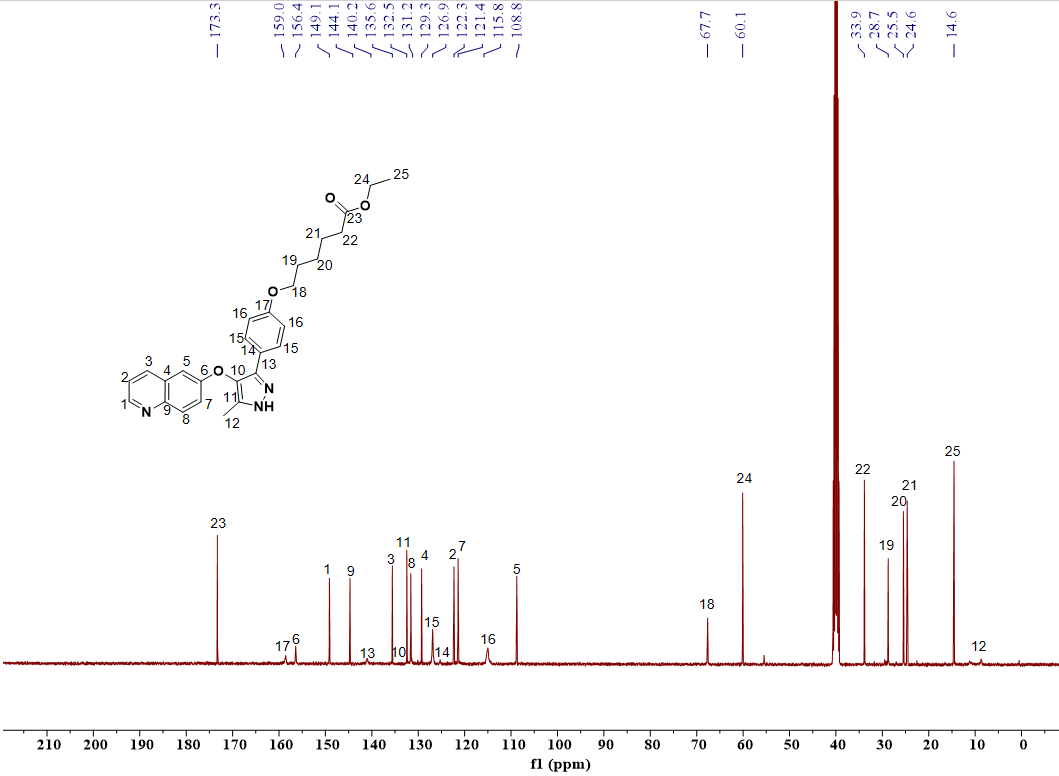


^13^C NMR spectra of compound **6c**


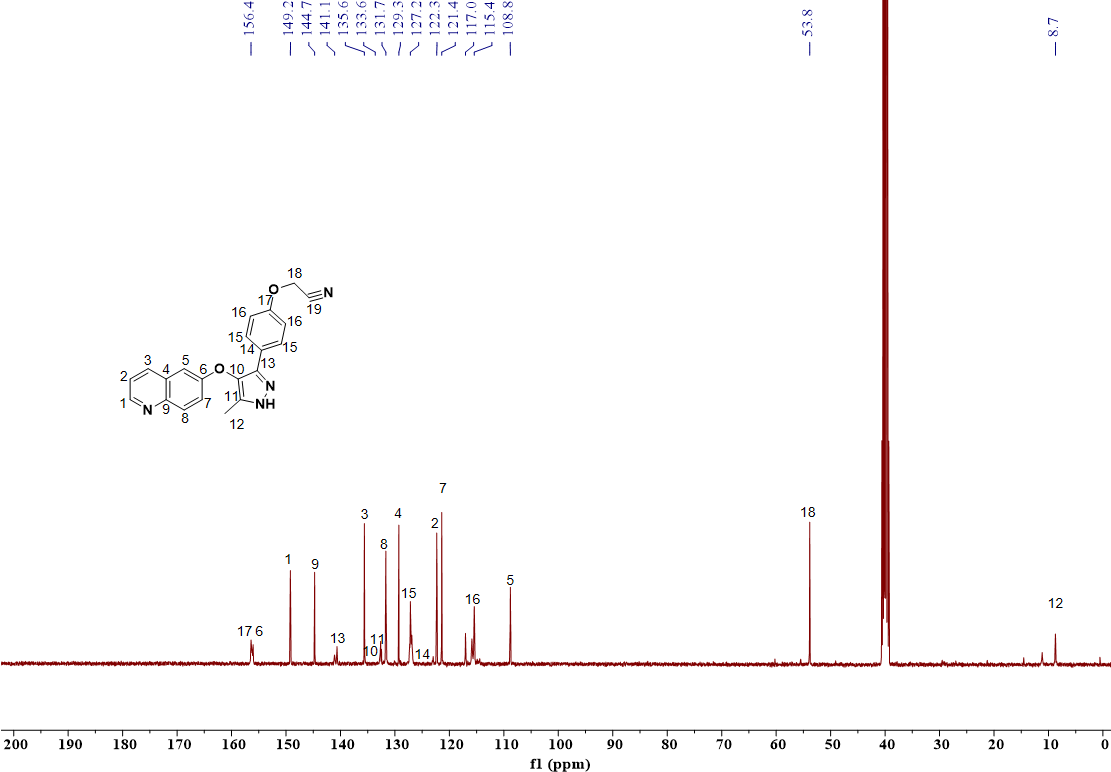


^13^C NMR spectra of compound **6d**


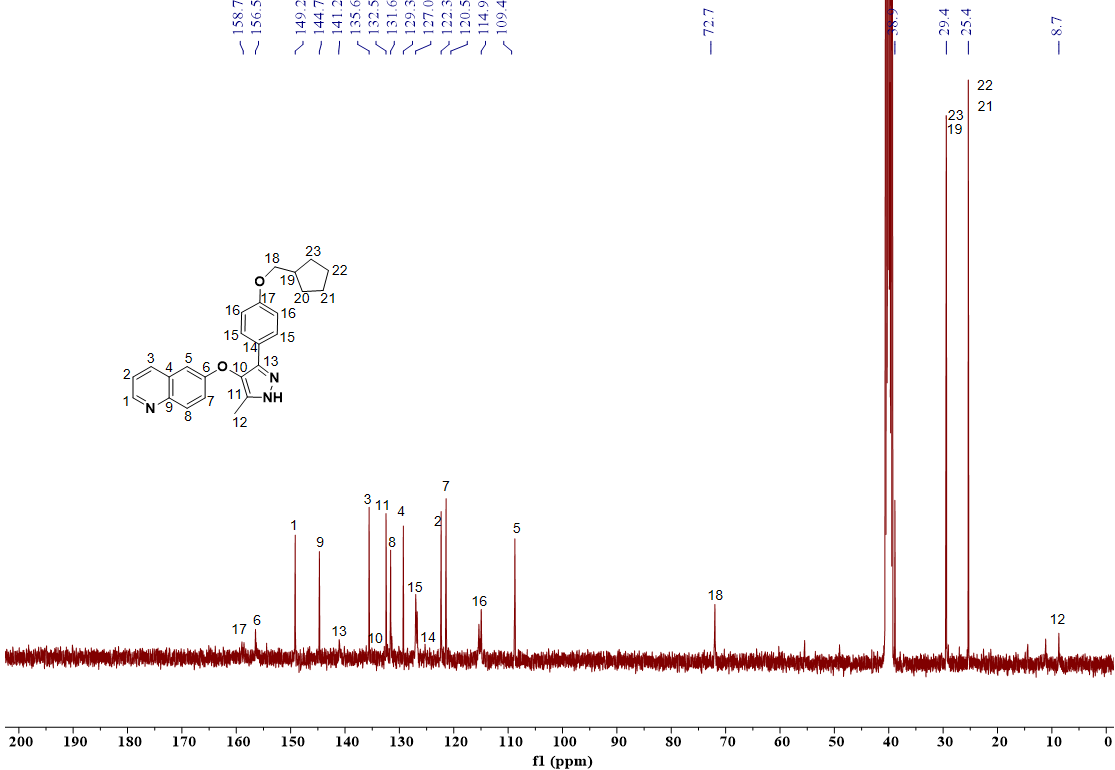


^13^C NMR spectra of compound **6e**


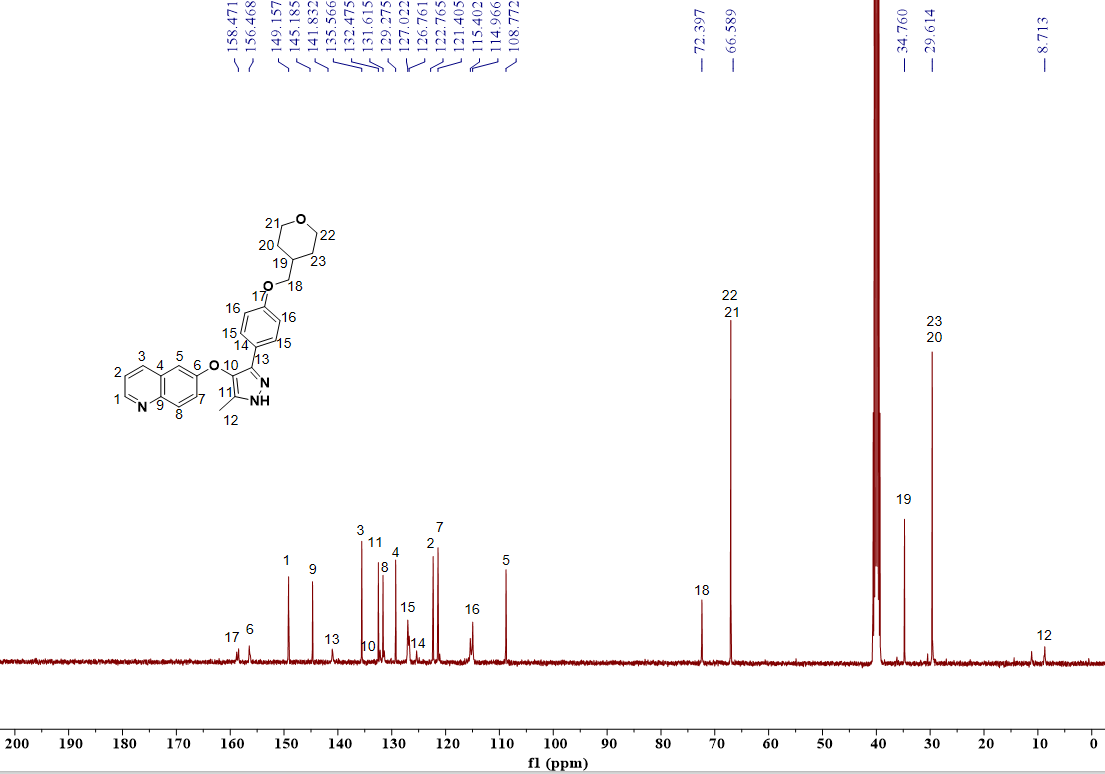


^13^C NMR spectra of compound **6f**


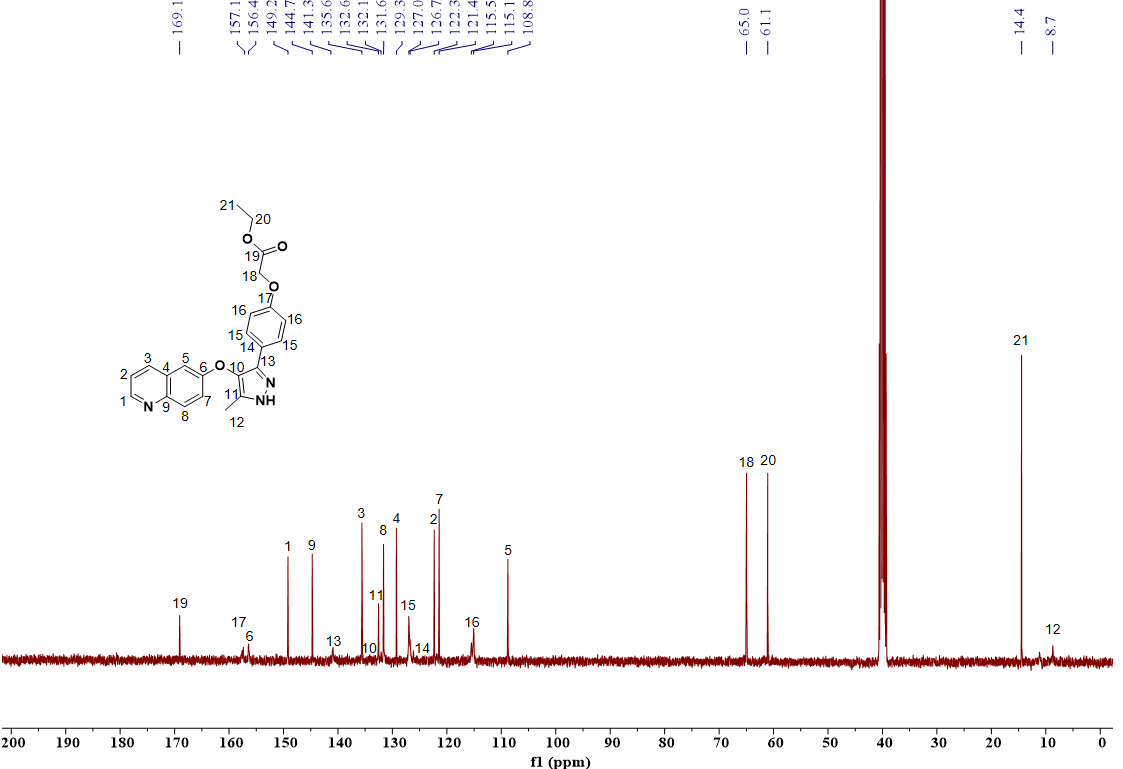


^13^C NMR spectra of compound **6g**


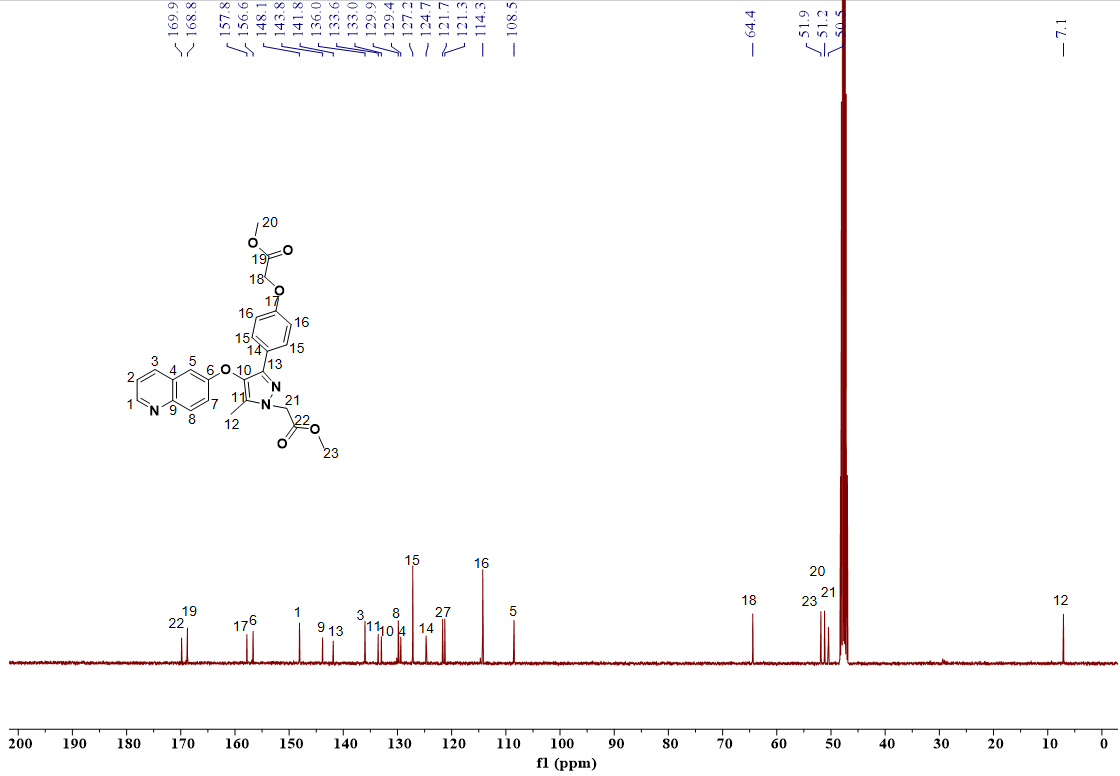


^13^C NMR spectra of compound **6h**


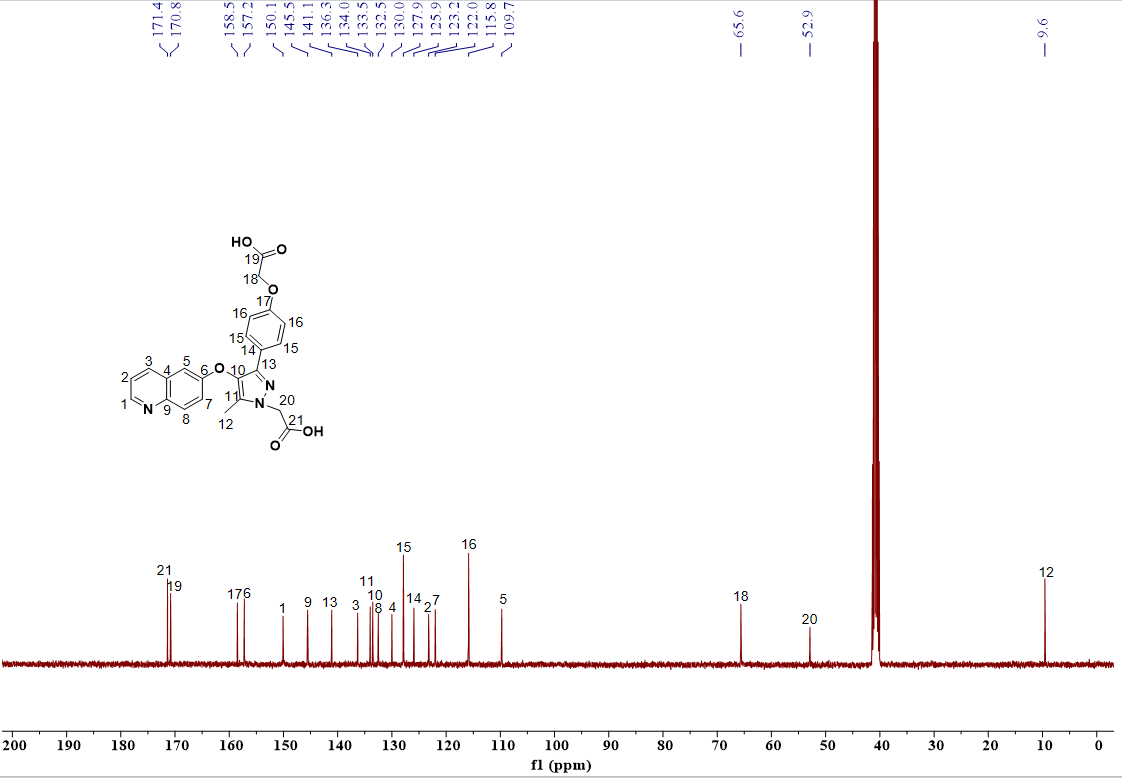


^13^C NMR spectra of compound **6i**


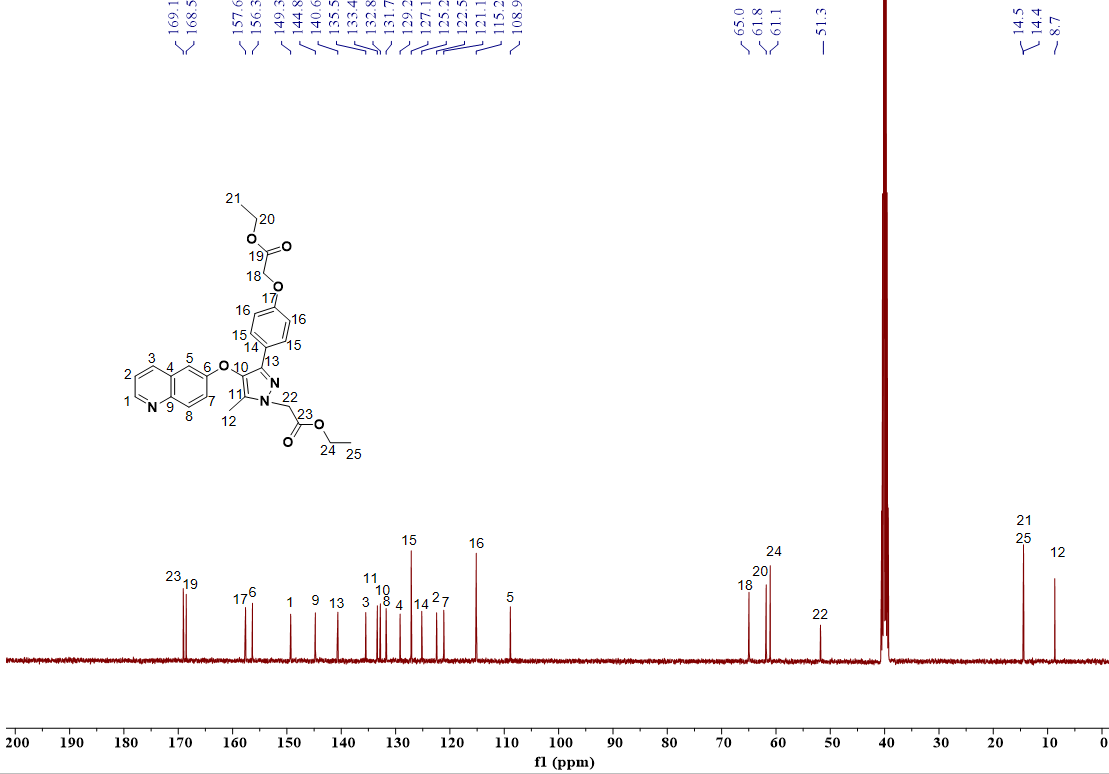


^13^C NMR spectra of compound **6j**


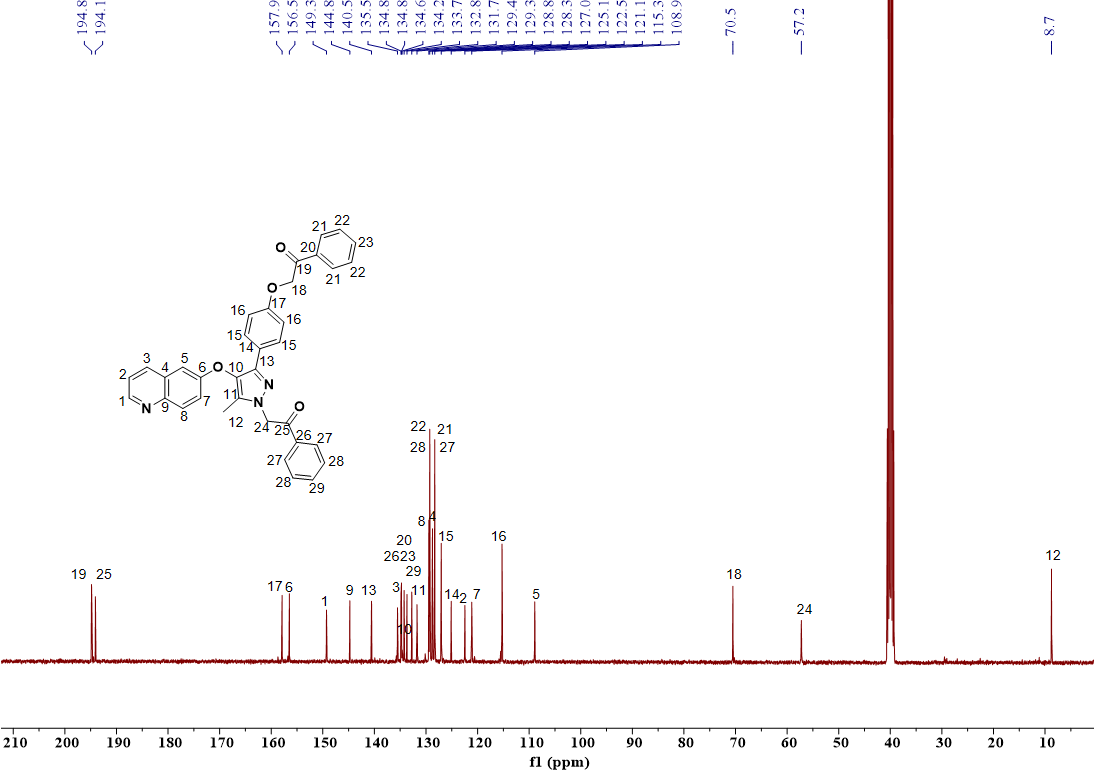


^13^C NMR spectra of compound **6k**


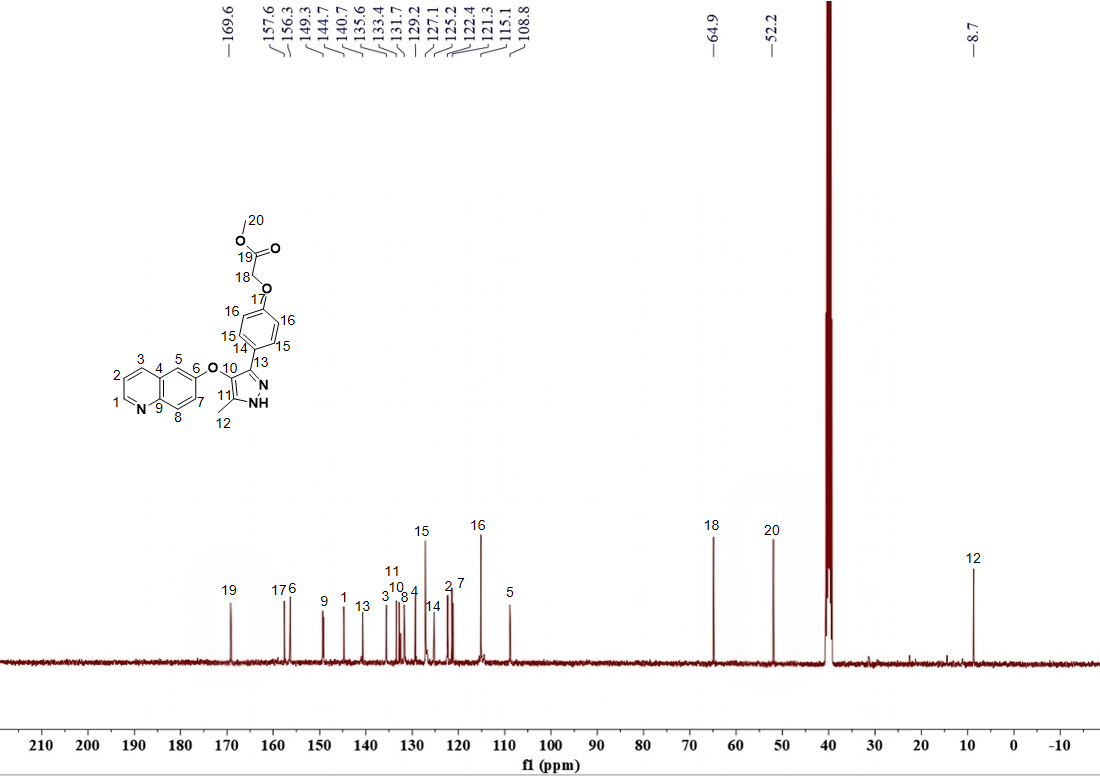


^13^C NMR spectra of compound **7**


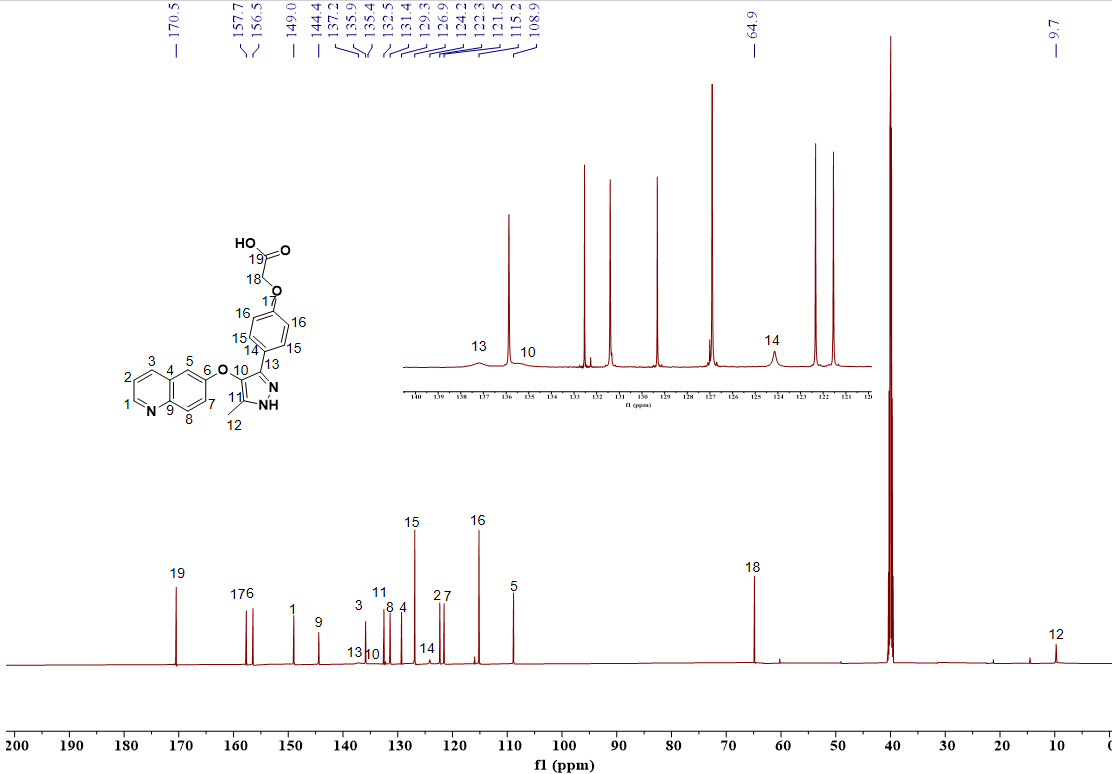


^13^C NMR spectra of compound **8**


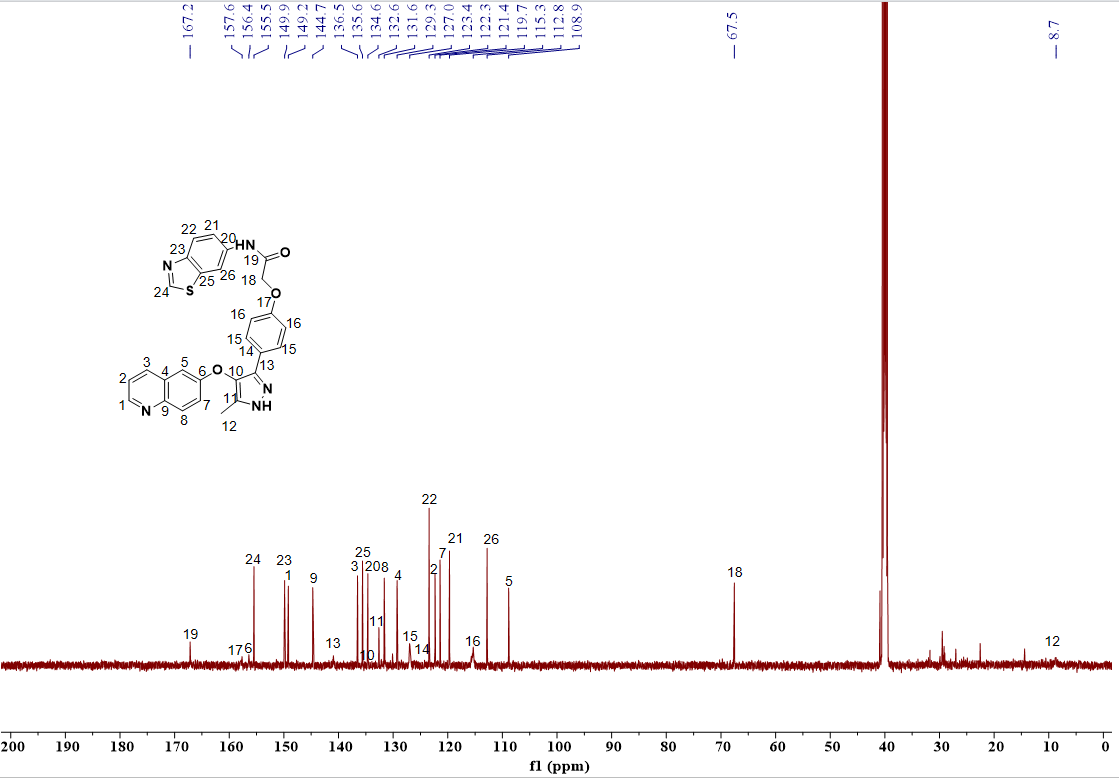


^13^C NMR spectra of compound **9a**


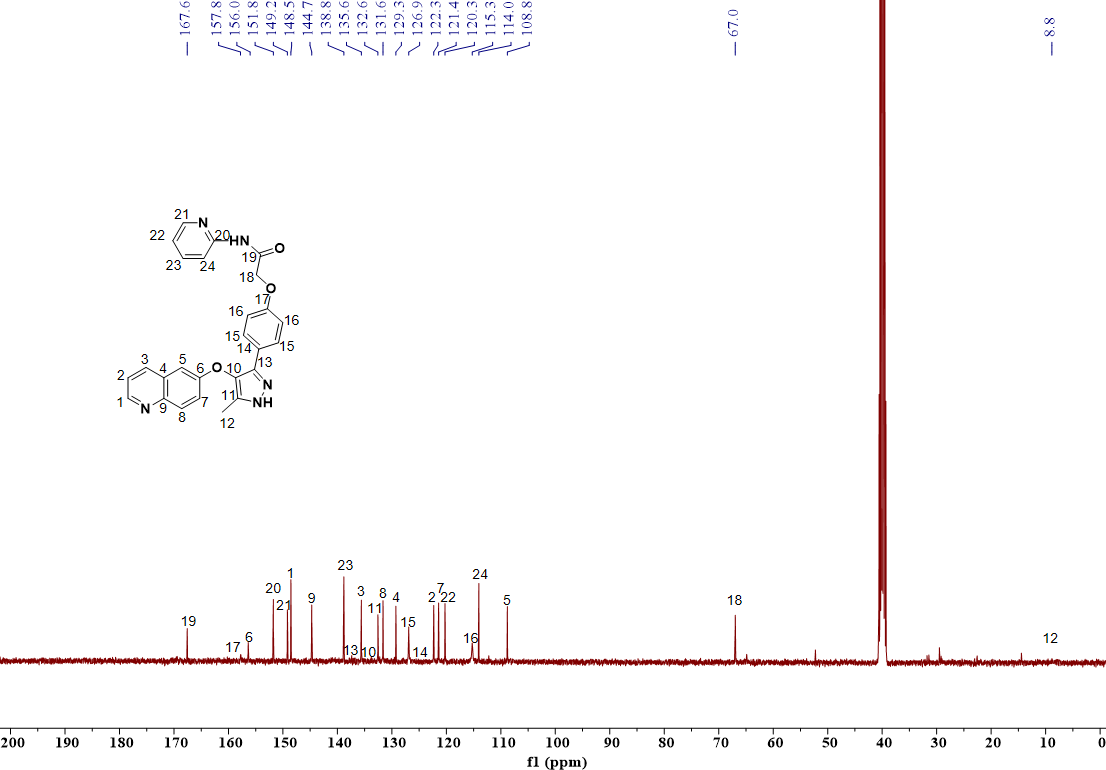


^13^C NMR spectra of compound **9b**


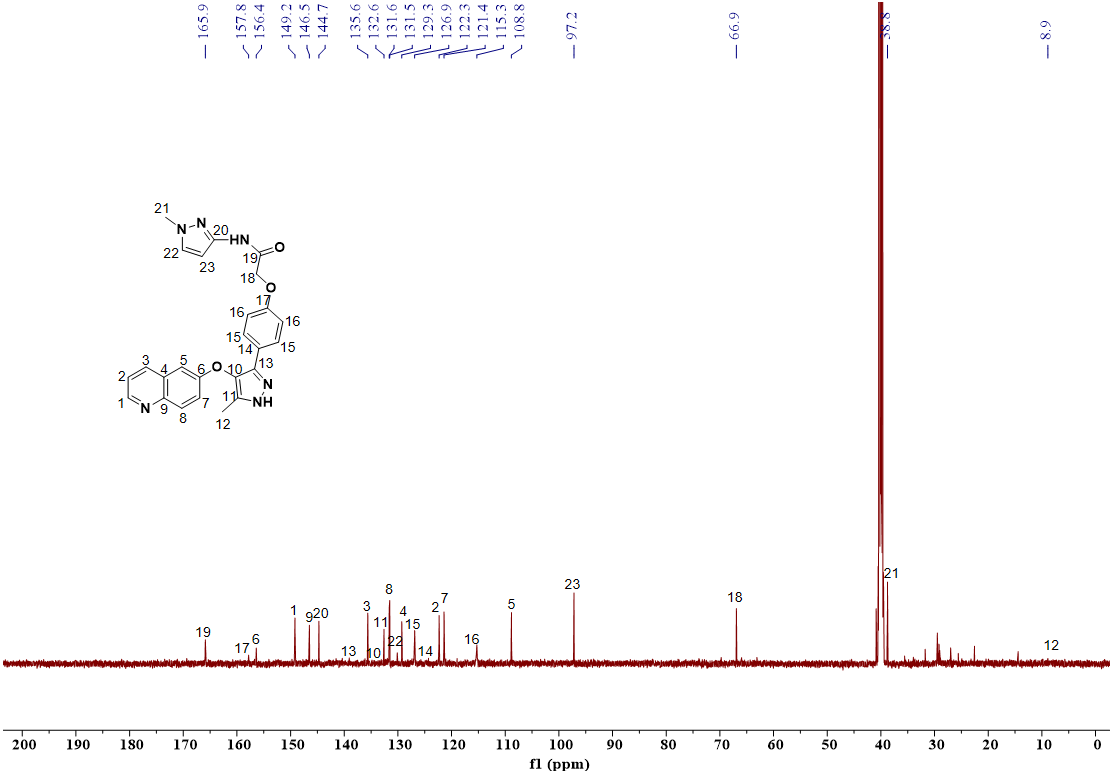


^13^C NMR spectra of compound **9c**


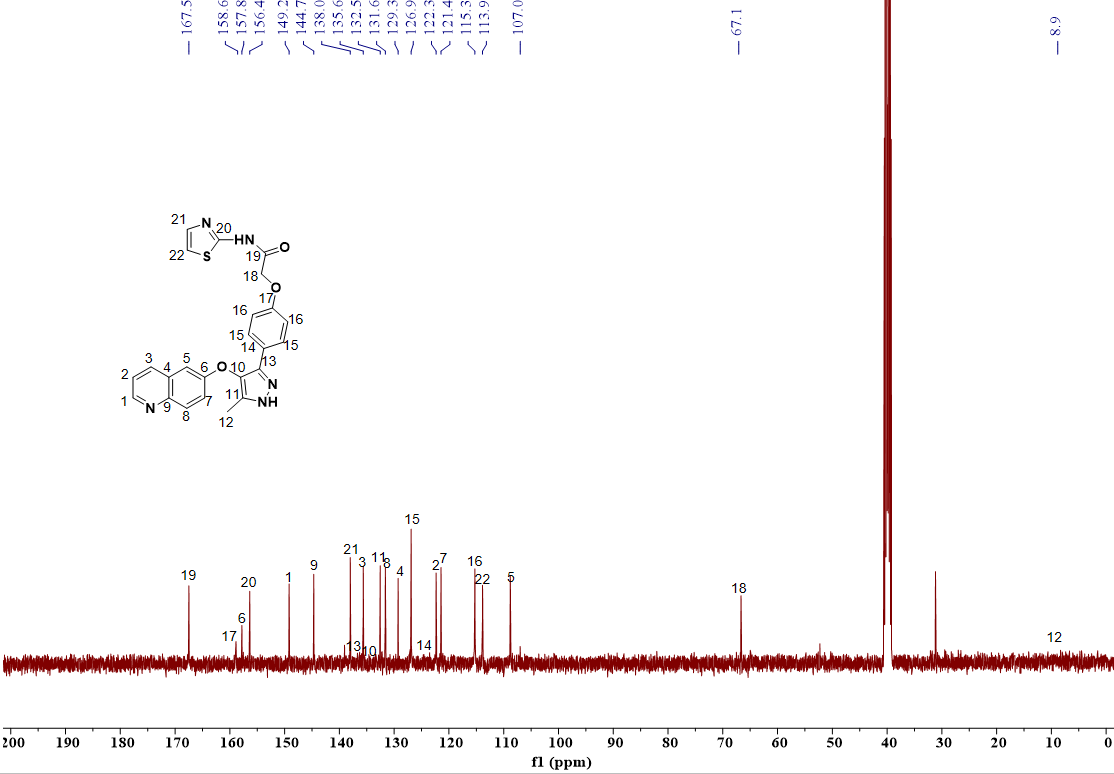


^13^C NMR spectra of compound **9d**


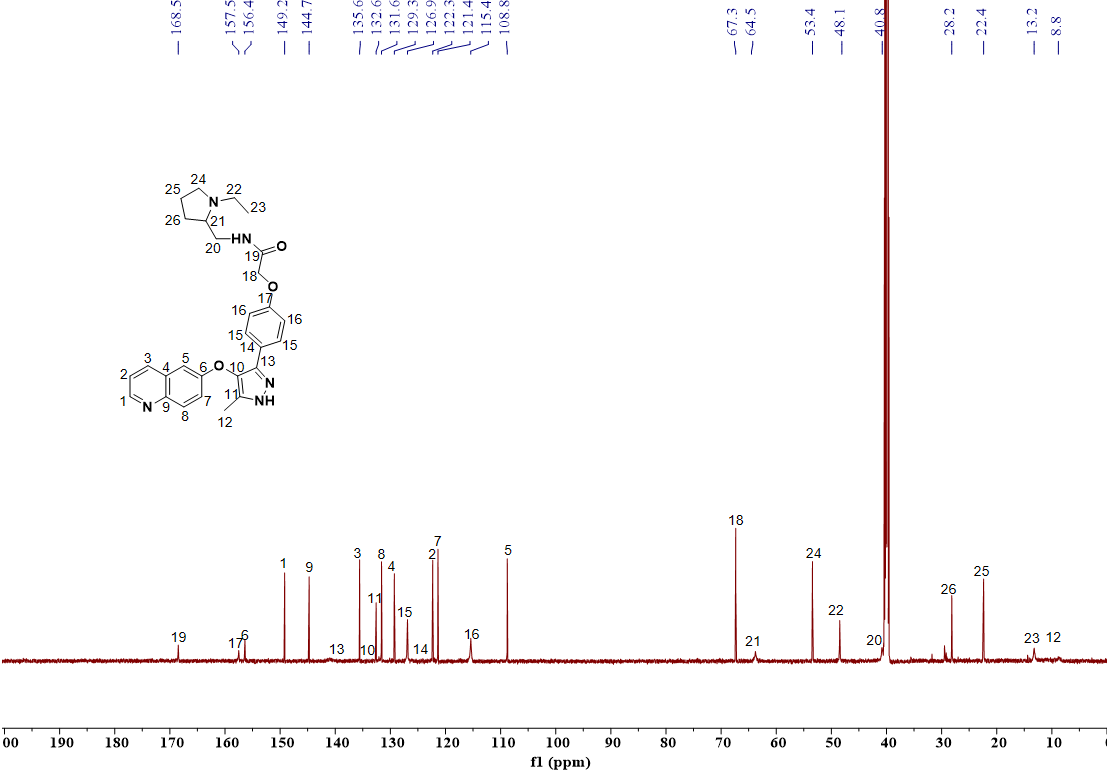


^13^C NMR spectra of compound **9e**


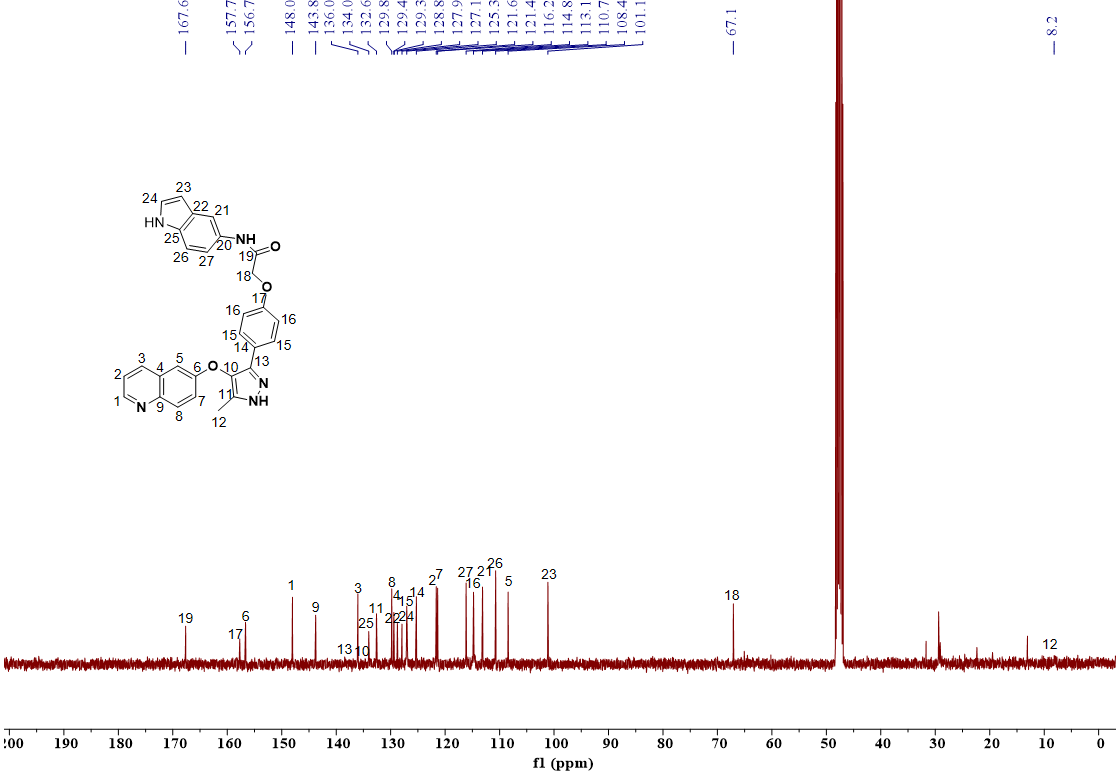


^13^C NMR spectra of compound **9f**


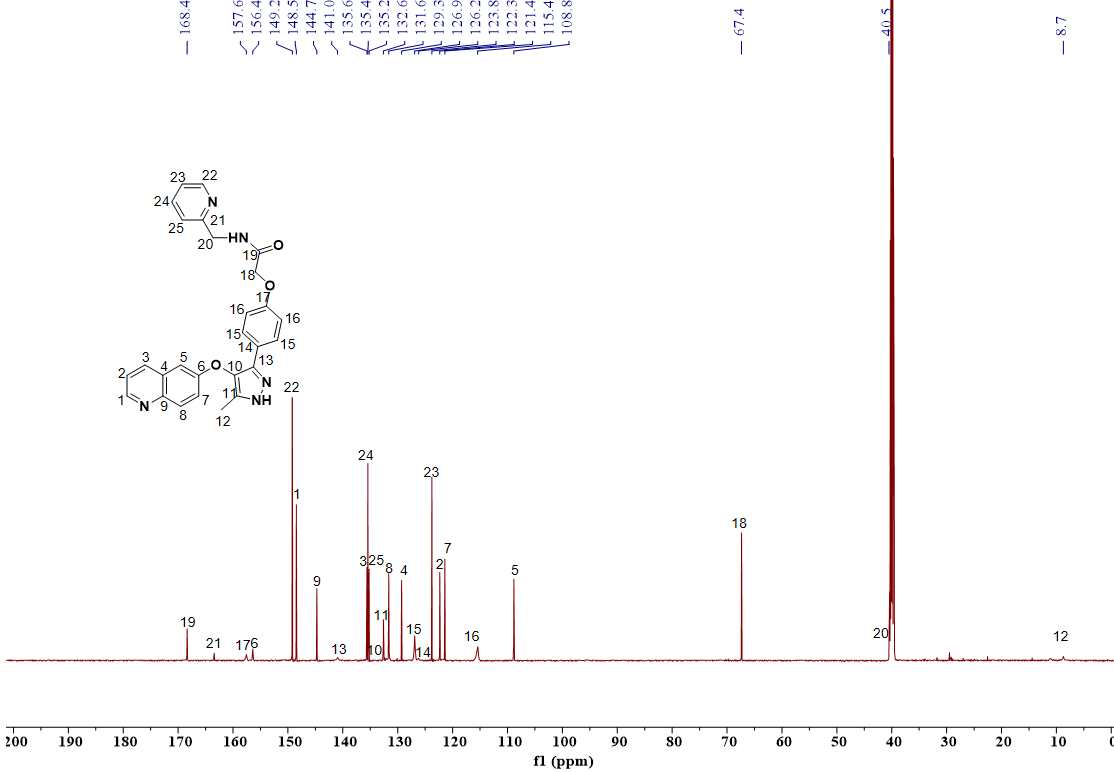


^13^C NMR spectra of compound **9g**


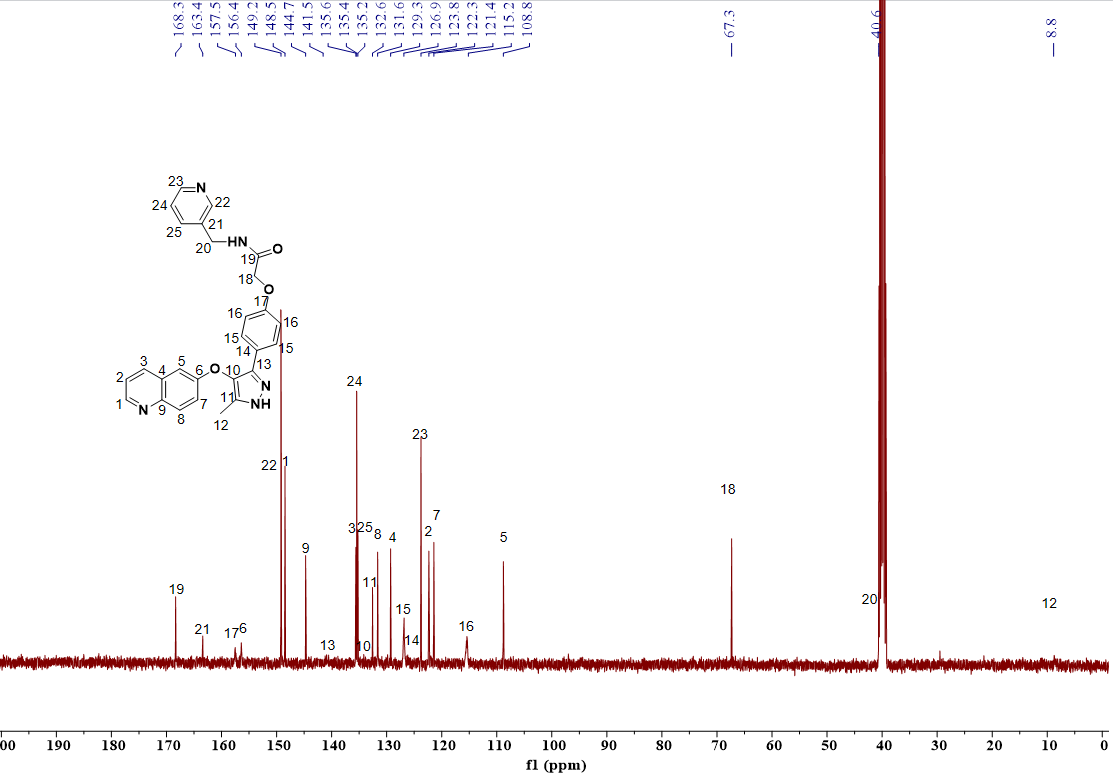


^13^C NMR spectra of compound **9h**


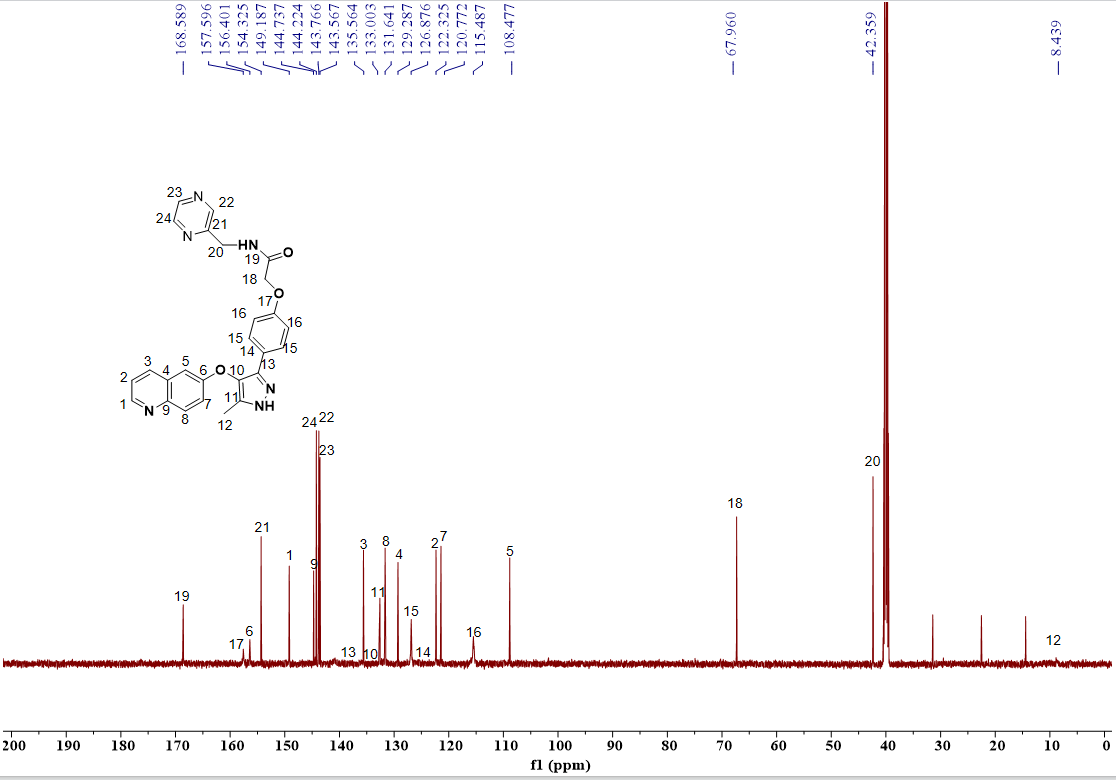


^13^C NMR spectra of compound **9i**


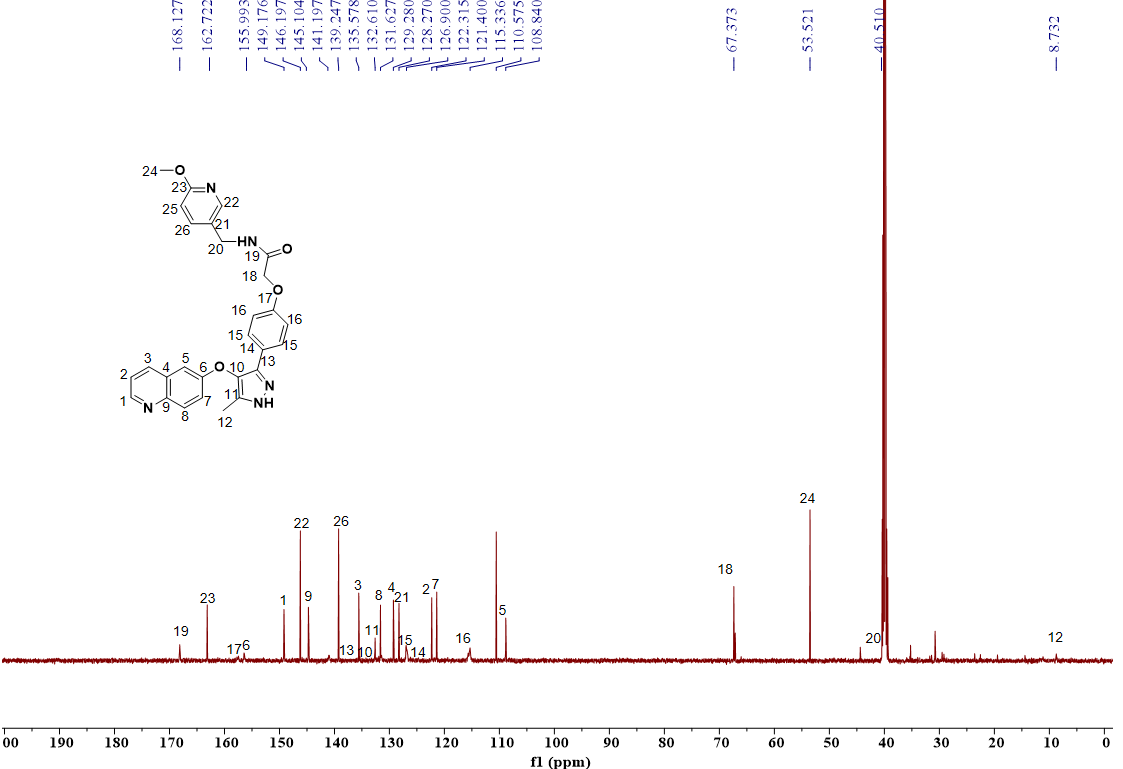


^13^C NMR spectra of compound **9j**


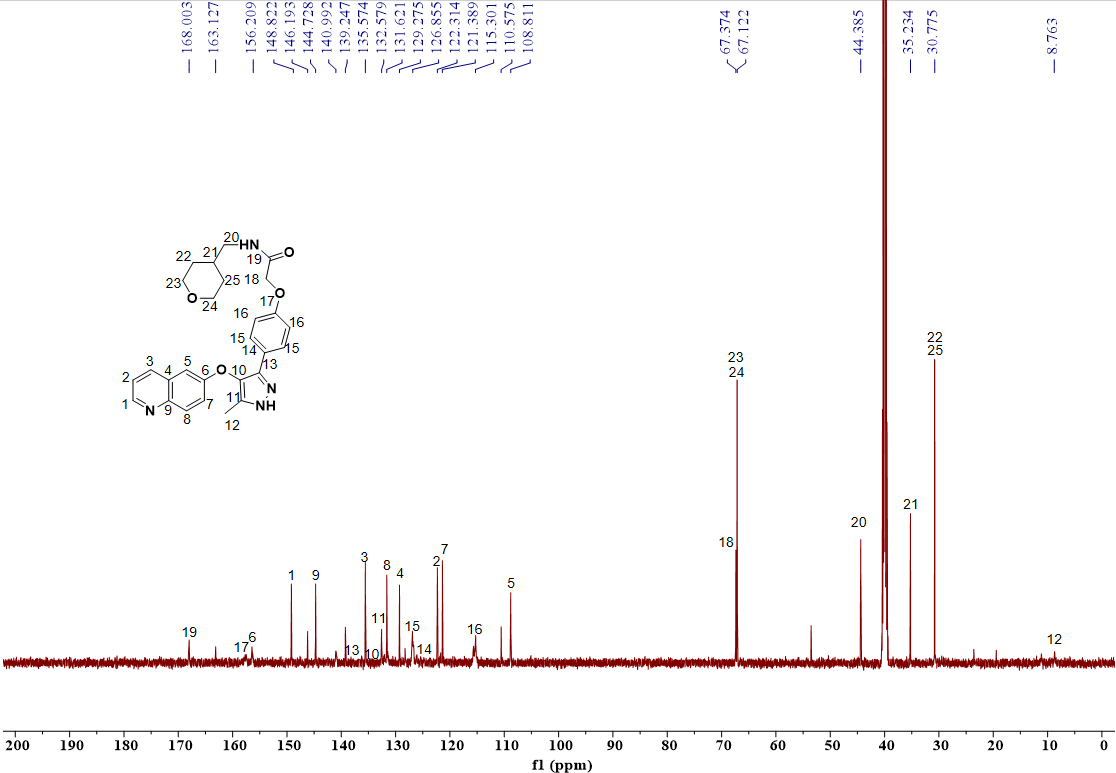


^13^C NMR spectra of compound **9k**


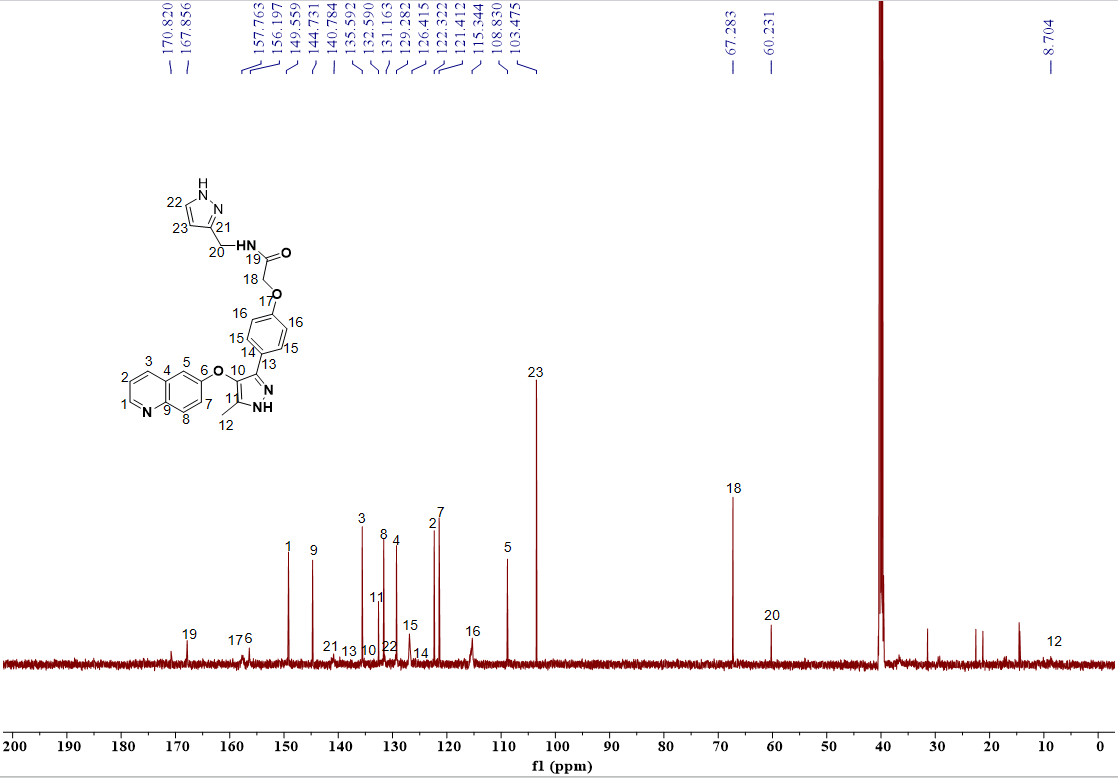


^13^C NMR spectra of compound **9l**

HRMS spectra of target compounds

Fragmentation and isotopic pattern analysis


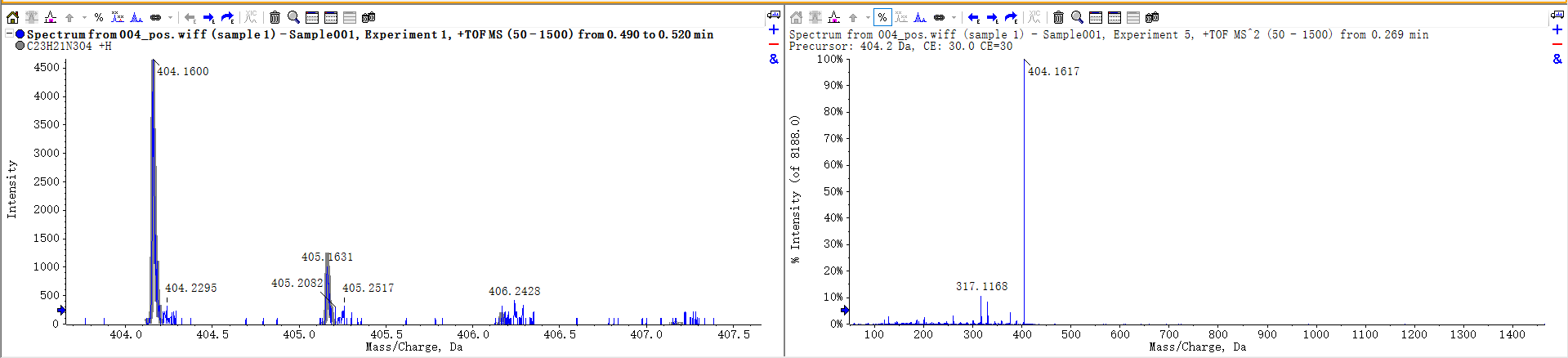


HRMS spectra of compound **6g**

m/z 404=[M+H]^+^

The prominent fragment at m/z 317.1168 suggests cleavage of the ether group. m/z 317=[M+H]^+^-87.

Inferred pathway of pyrolysis


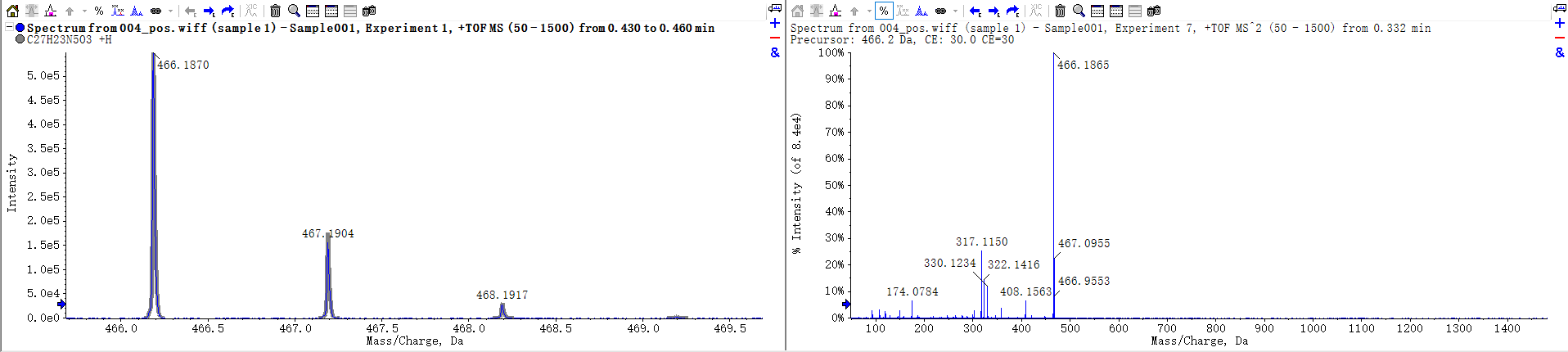


HRMS spectra of compound **9h**

m/z 466=[M+H]^+^

The fragment observed at m/z 317.1150 arises from cleavage of the aryl ether bond (C–O). Subsequent elimination of the quinoline-containing moiety as a neutral fragment yields the ion at m/z 174.0784. And the signal at m/z 330.1234 is tentatively assigned to the loss of the N-(pyridin-3-ylmethyl)acetamide group. m/z 317=[M+H]^+^-149, m/z 174=[M+H]^+^-149-143, m/z 330=[M+H]^+^-136.

Inferred pathway of pyrolysis

The isotopic patterns and fragment peaks of other compounds are similar to those of the above two compounds.


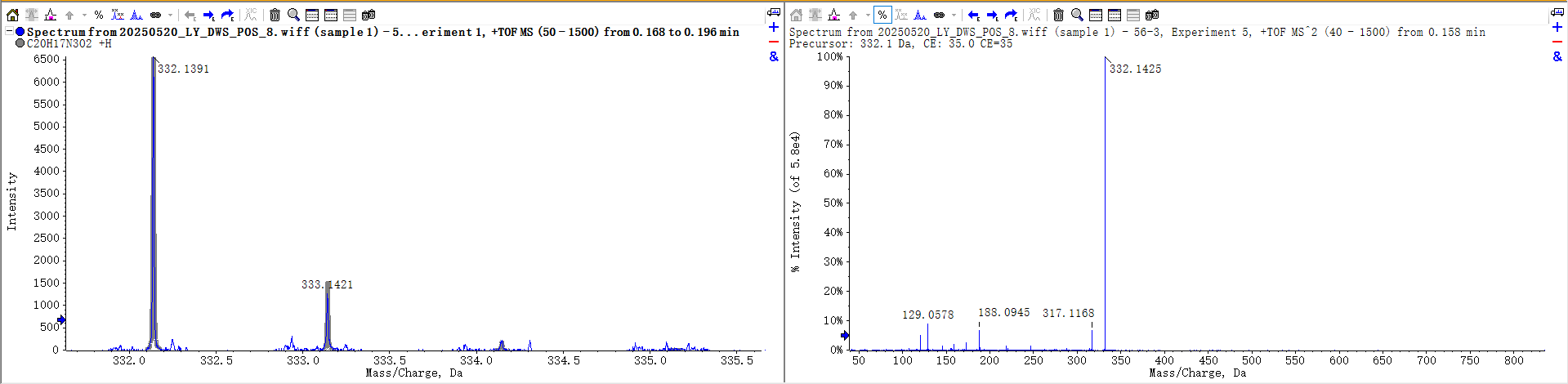


HRMS spectra of compound **4**


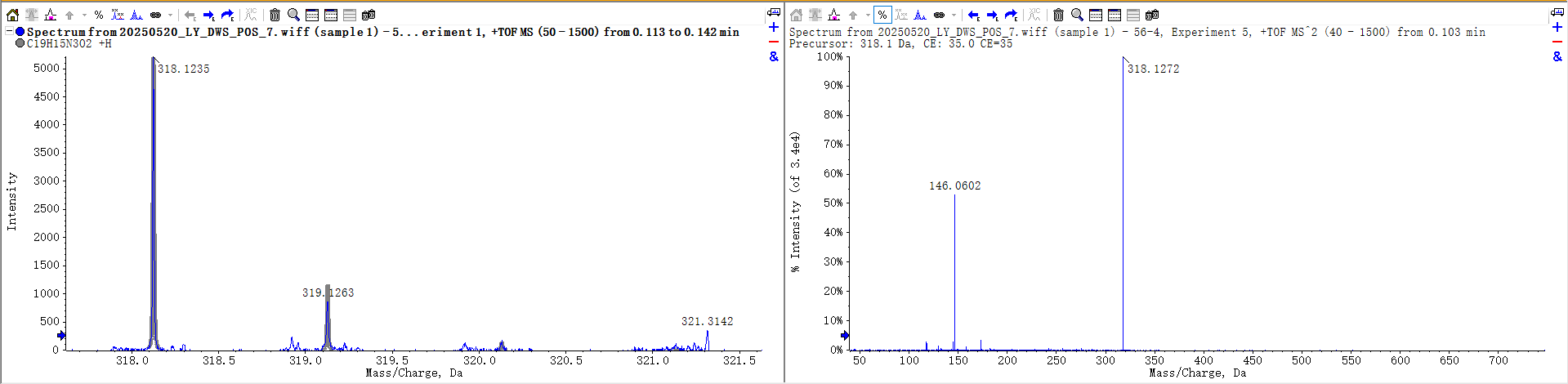


HRMS spectra of compound **5**


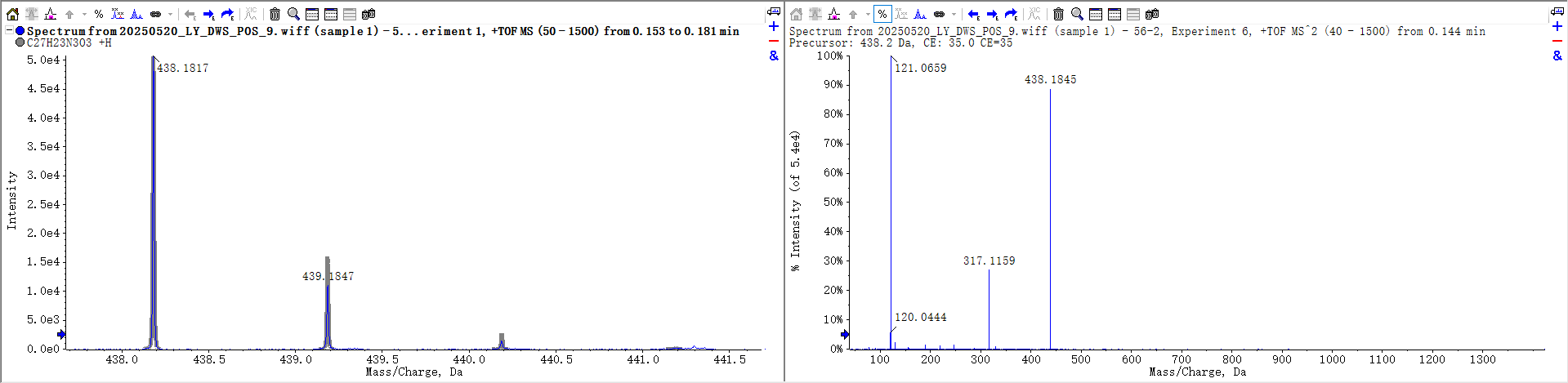


HRMS spectra of compound **6a**


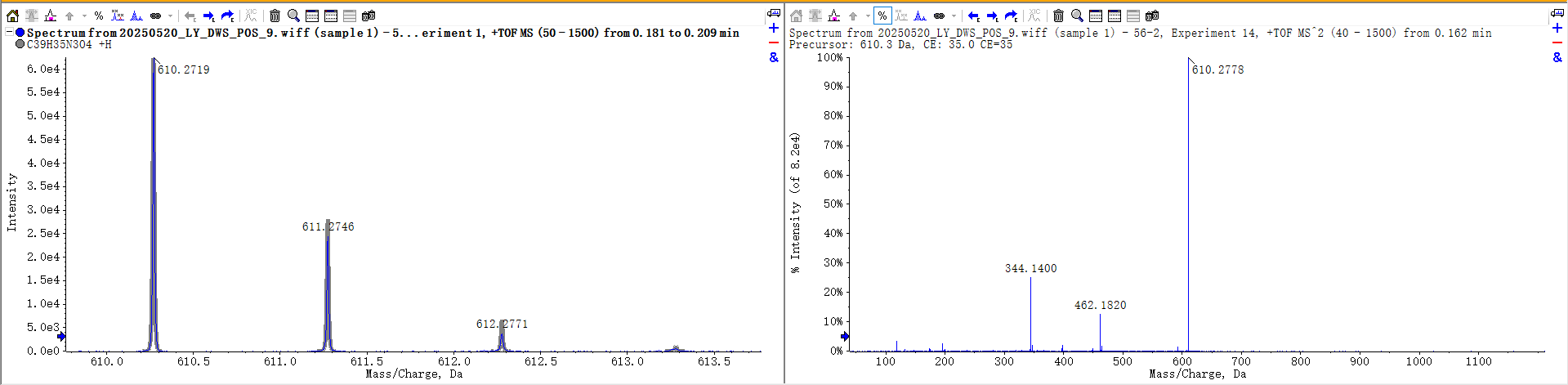


HRMS spectra of compound **6b**


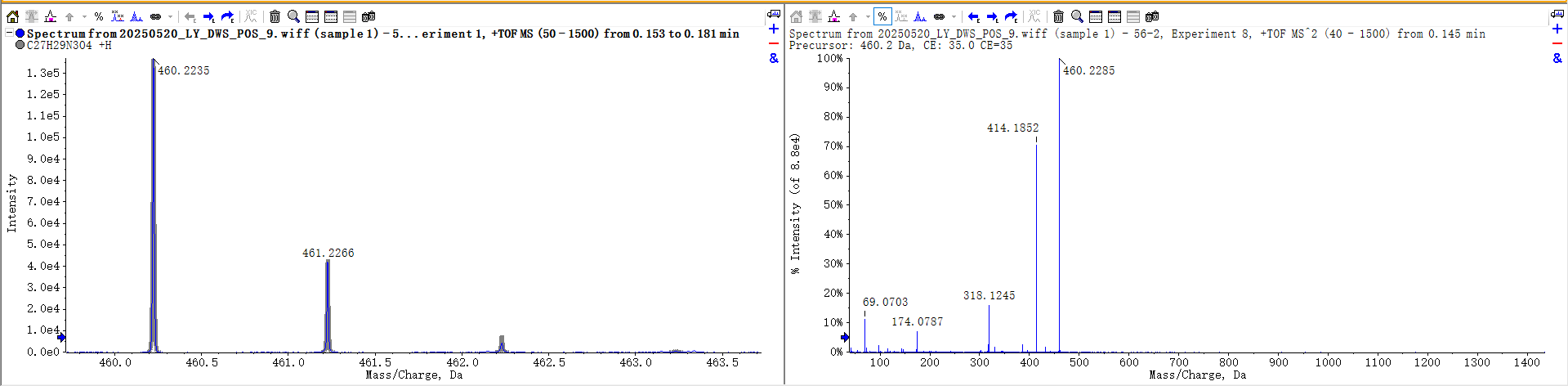


HRMS spectra of compound **6c**


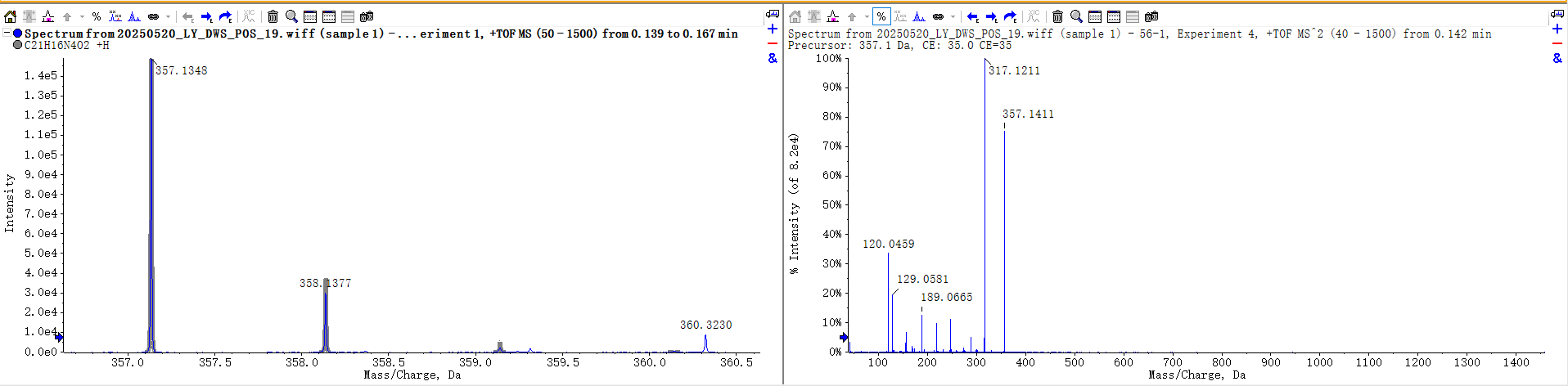


HRMS spectra of compound **6d**


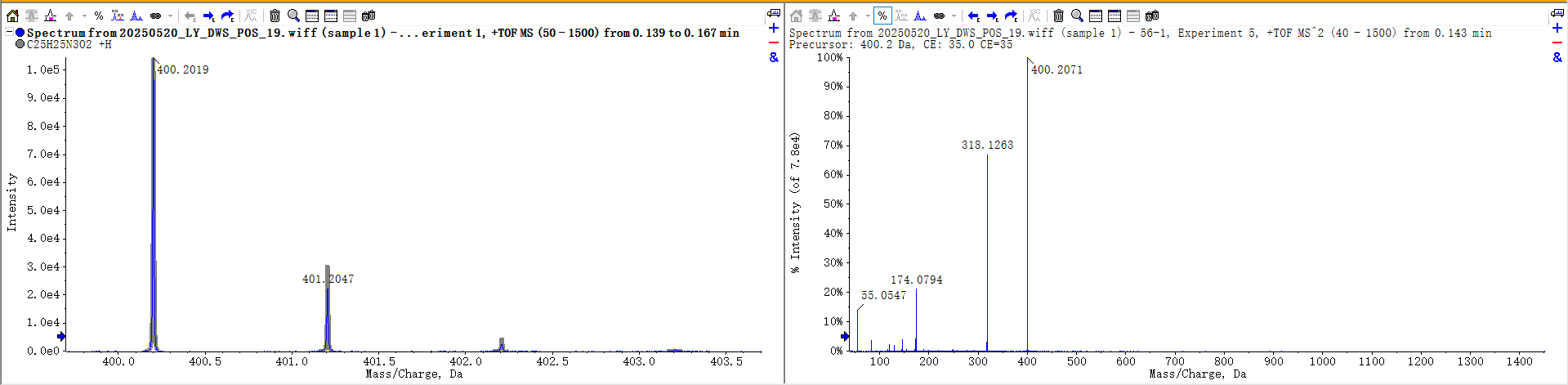


HRMS spectra of compound **6e**


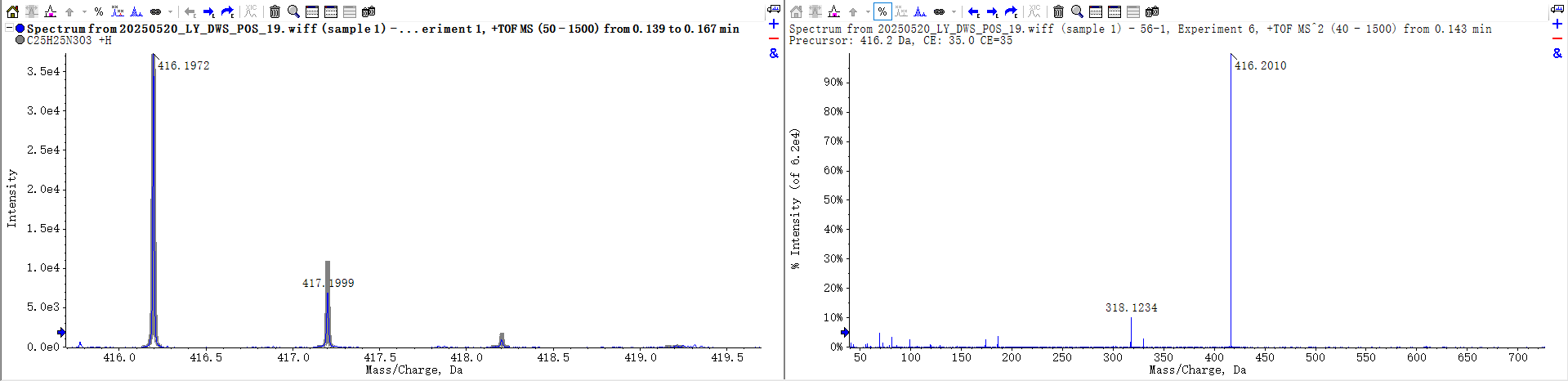


HRMS spectra of compound **6f**


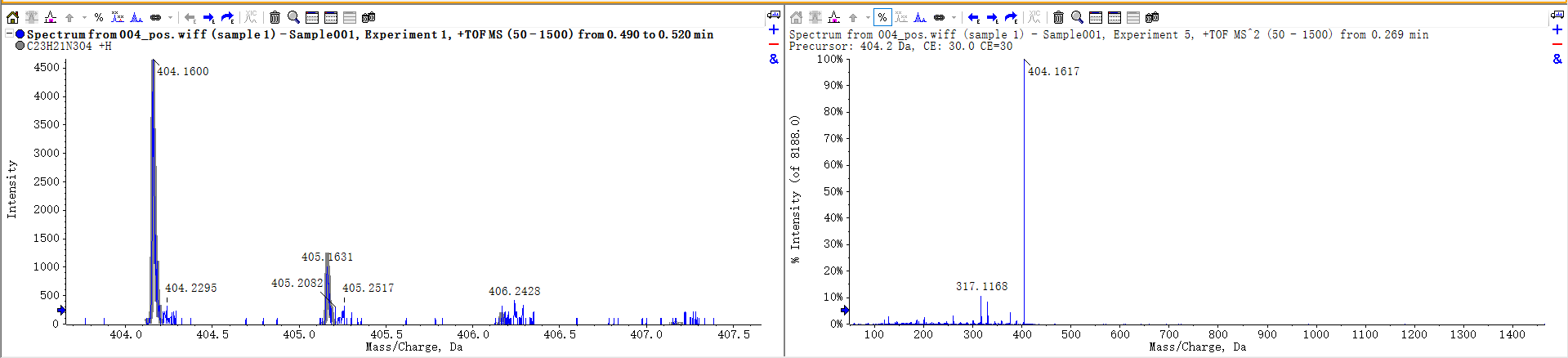


HRMS spectra of compound **6g**


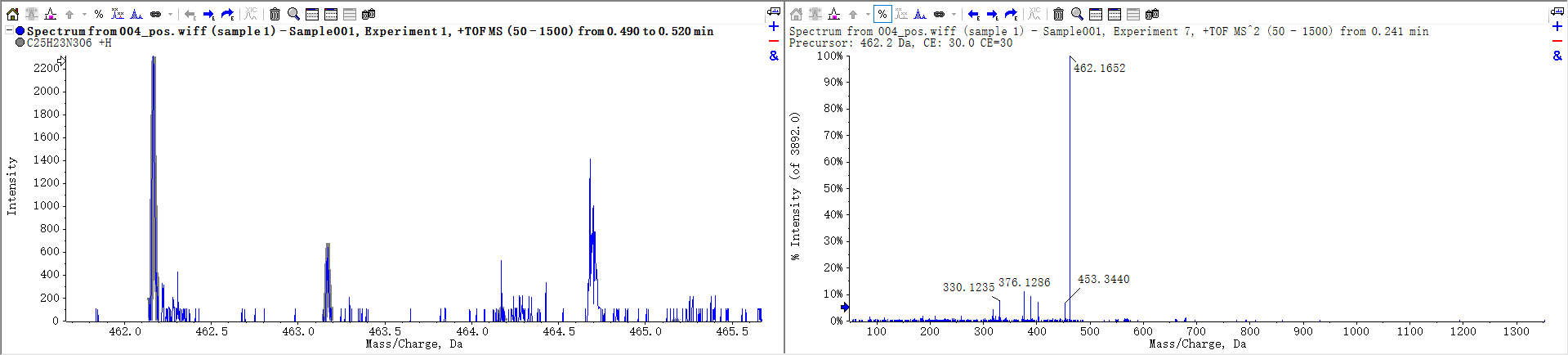


HRMS spectra of compound **6h**


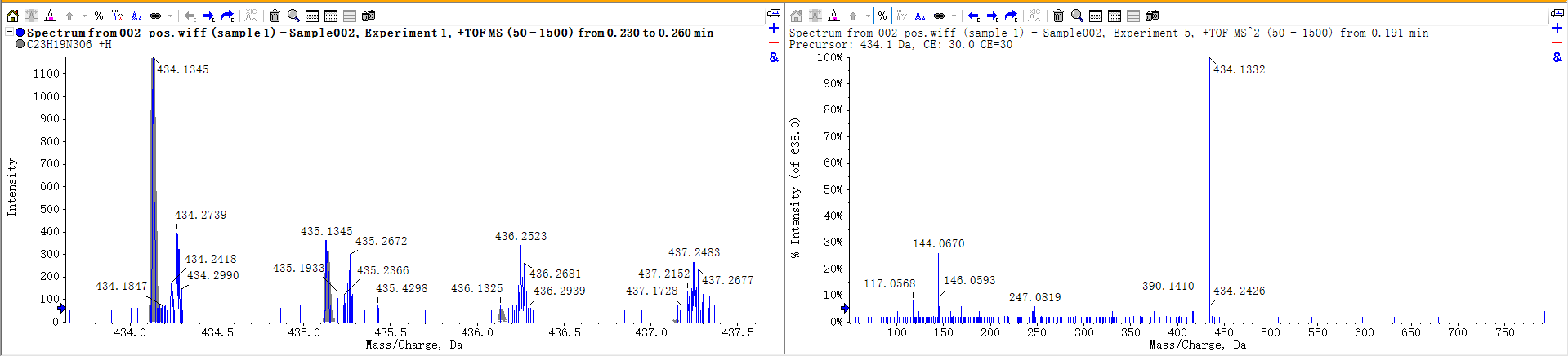


HRMS spectra of compound **6i**


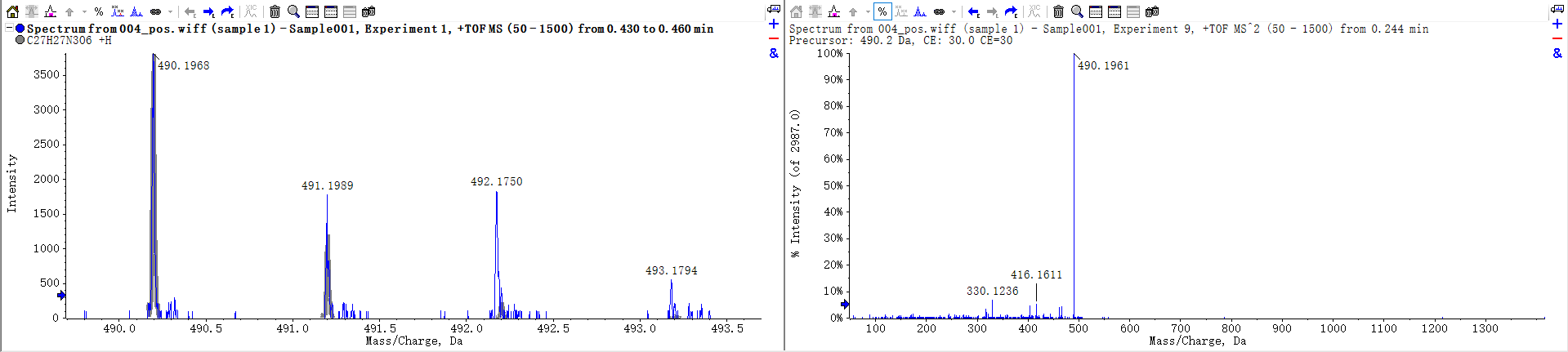


HRMS spectra of compound **6j**


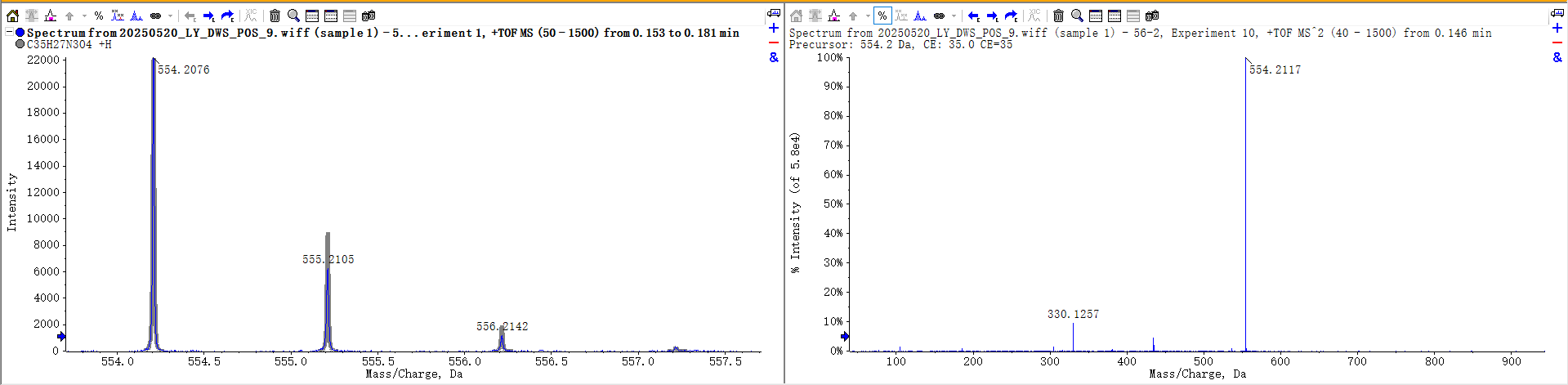


HRMS spectra of compound **6k**


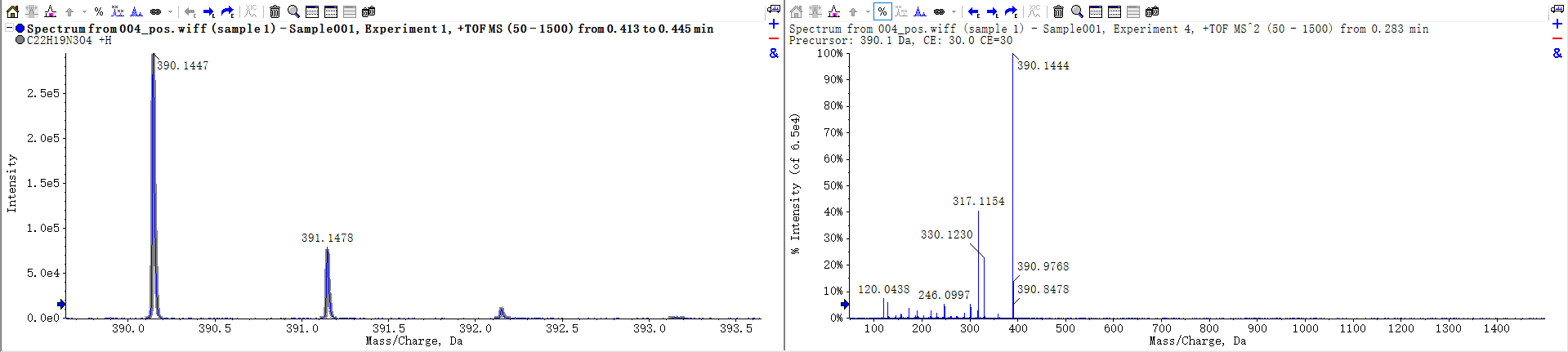


HRMS spectra of compound **7**


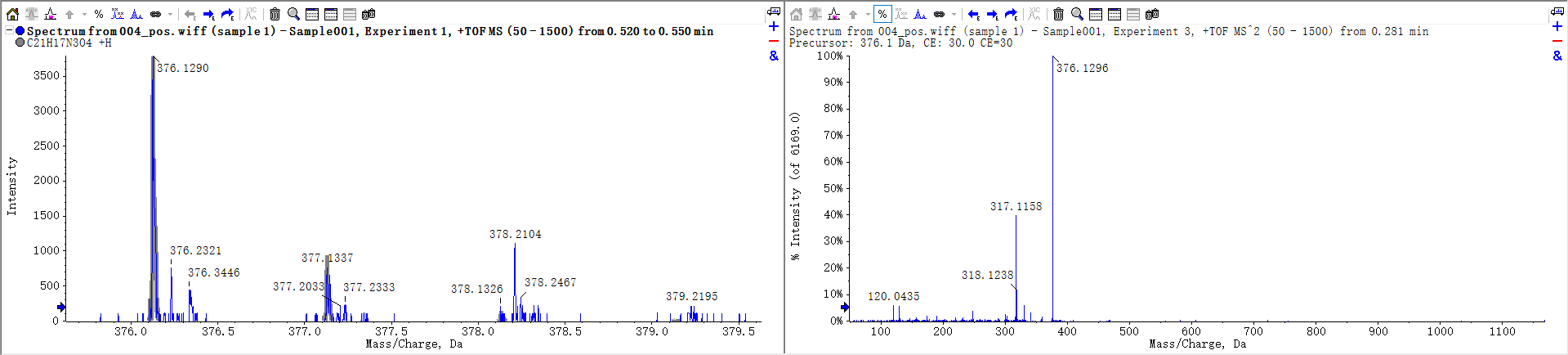


HRMS spectra of compound **8**


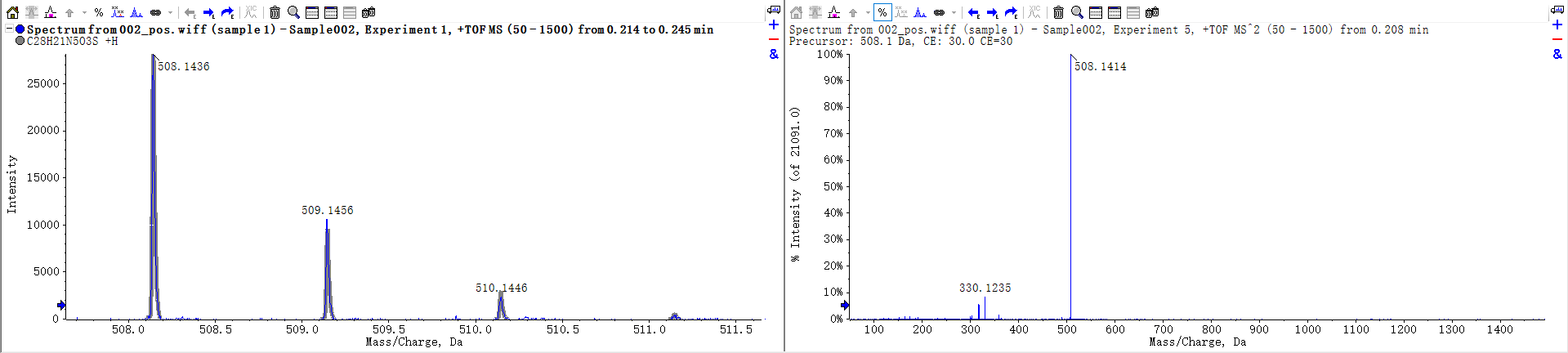


HRMS spectra of compound **9a**


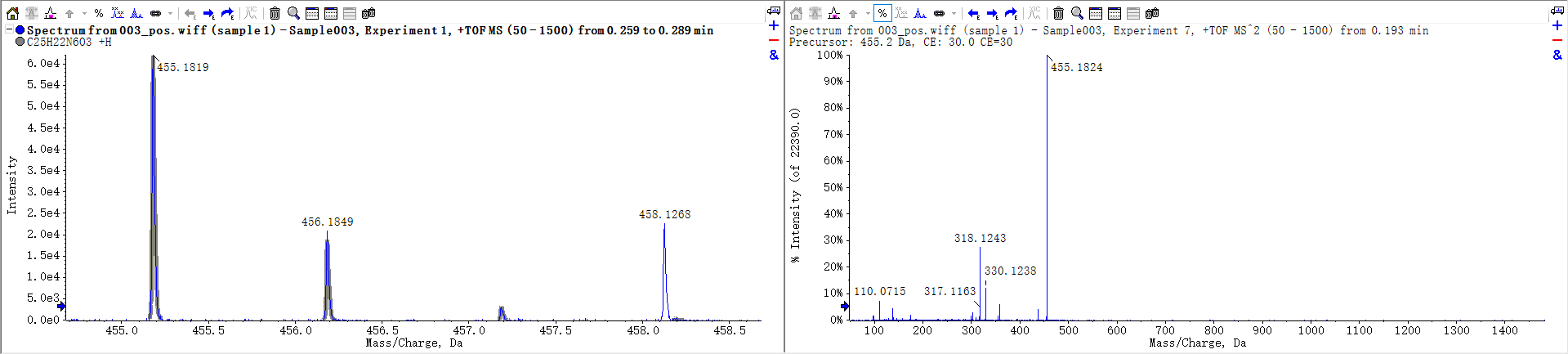


HRMS spectra of compound **9c**


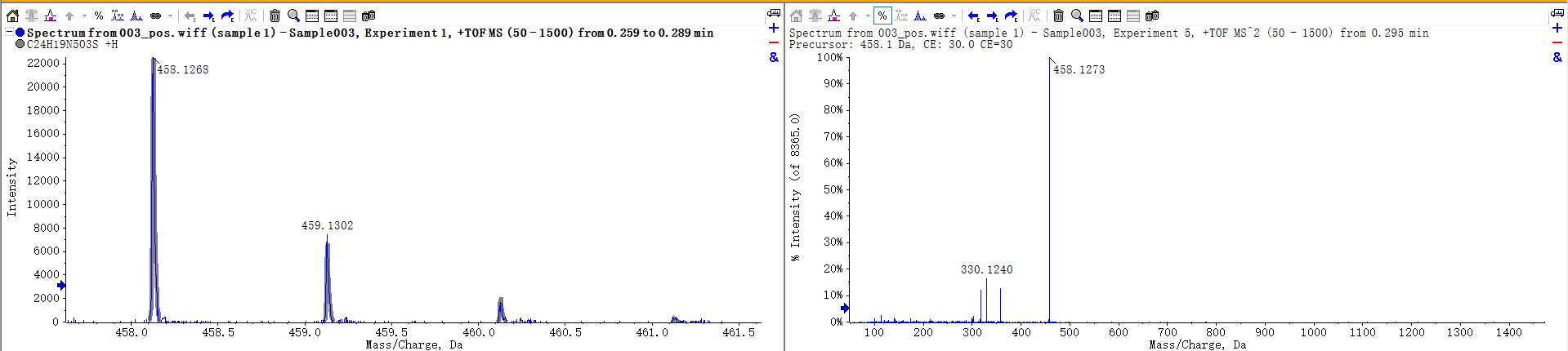


HRMS spectra of compound **9d**


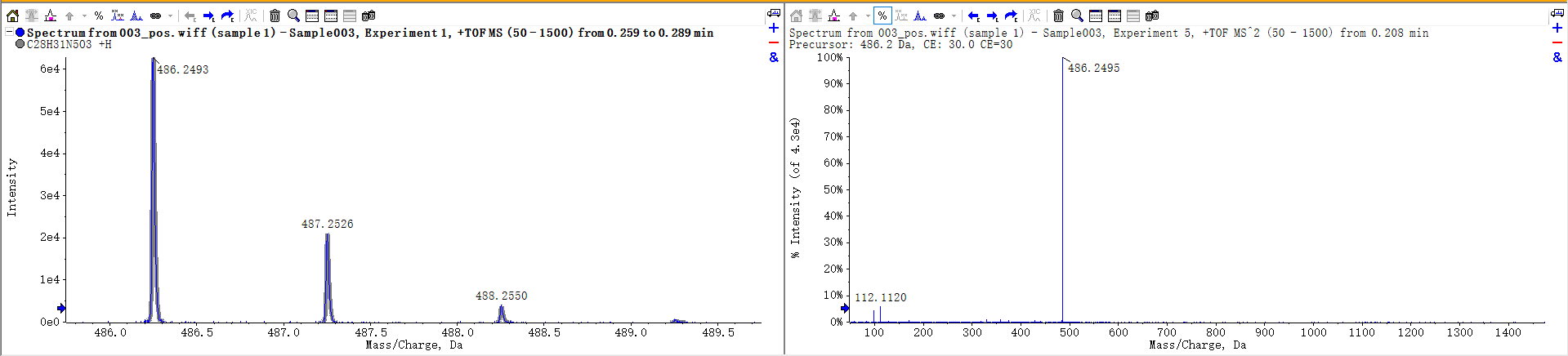


HRMS spectra of compound **9e**


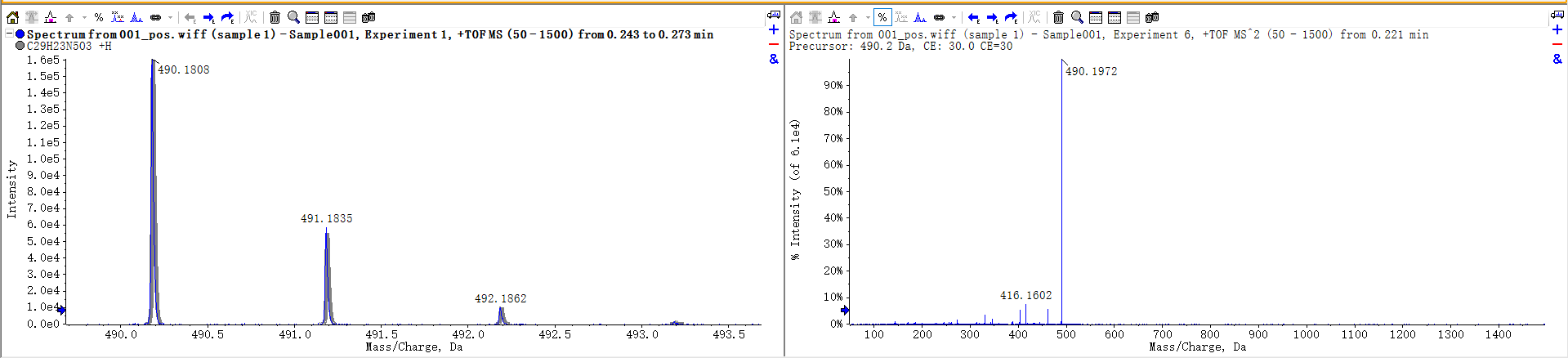


HRMS spectra of compound **9f**


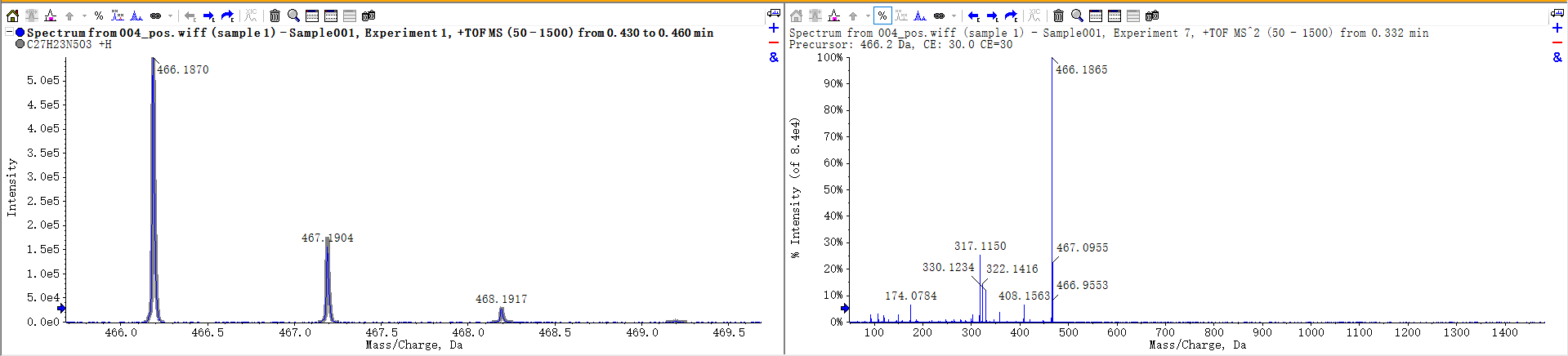


HRMS spectra of compound **9g**


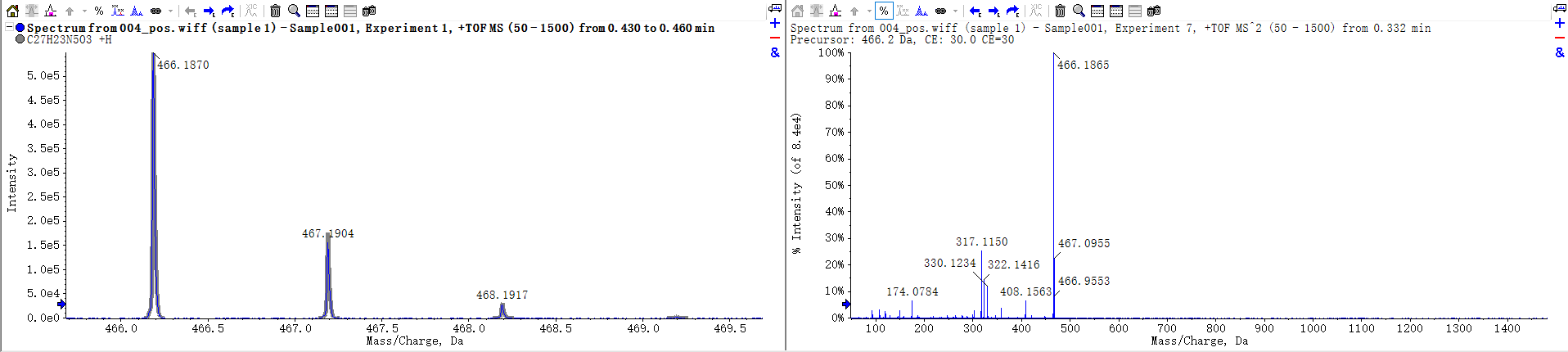


HRMS spectra of compound **9h**


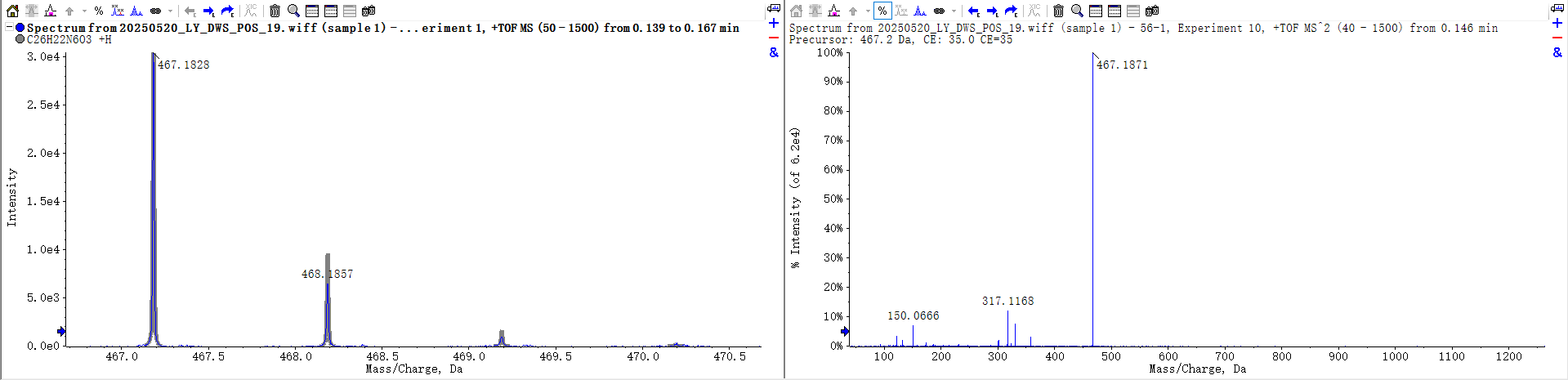


HRMS spectra of compound **9i**


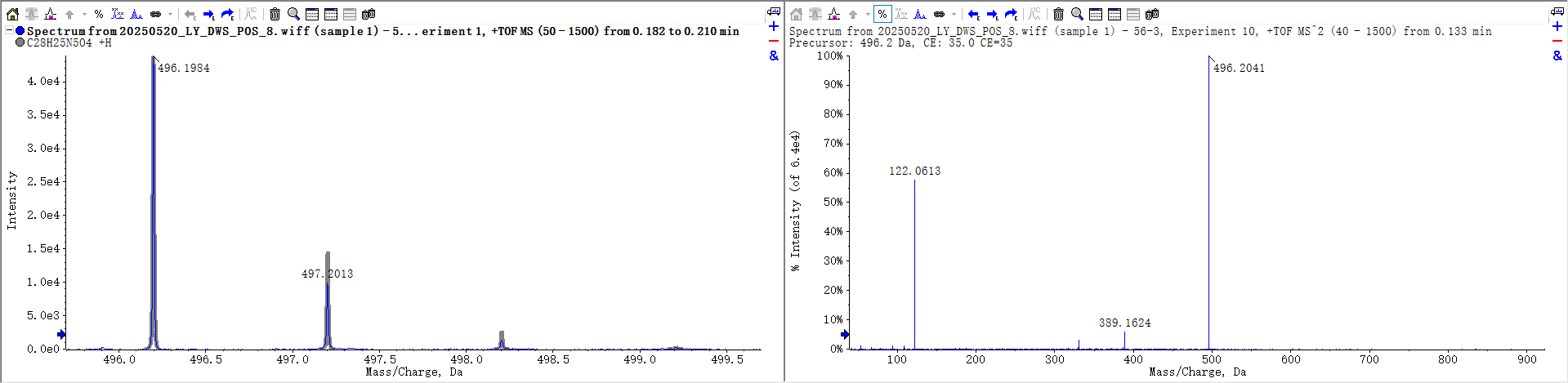


HRMS spectra of compound **9j**


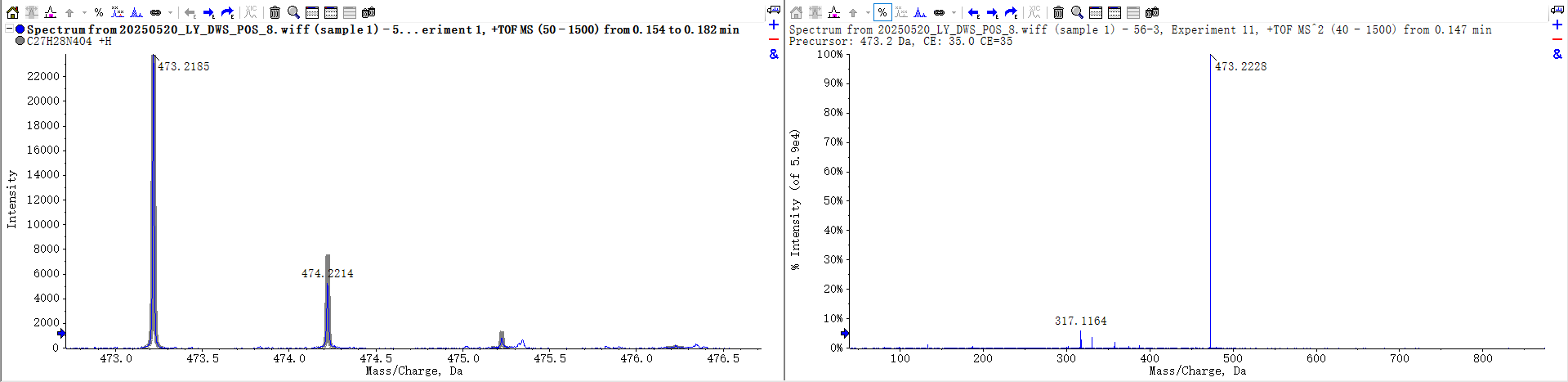


HRMS spectra of compound **9k**


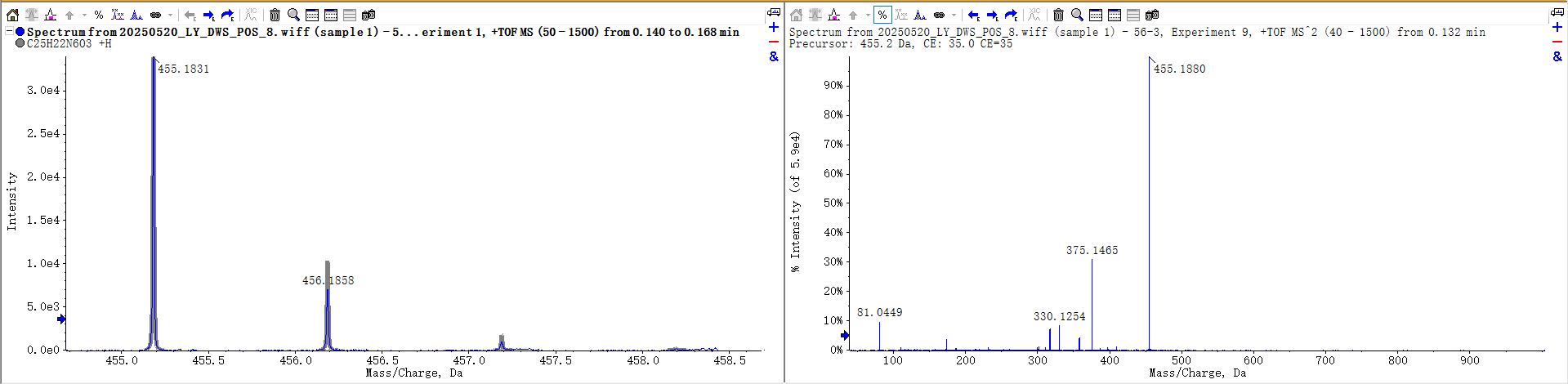


HRMS spectra of compound **9l**

HPLC spectra of target compounds


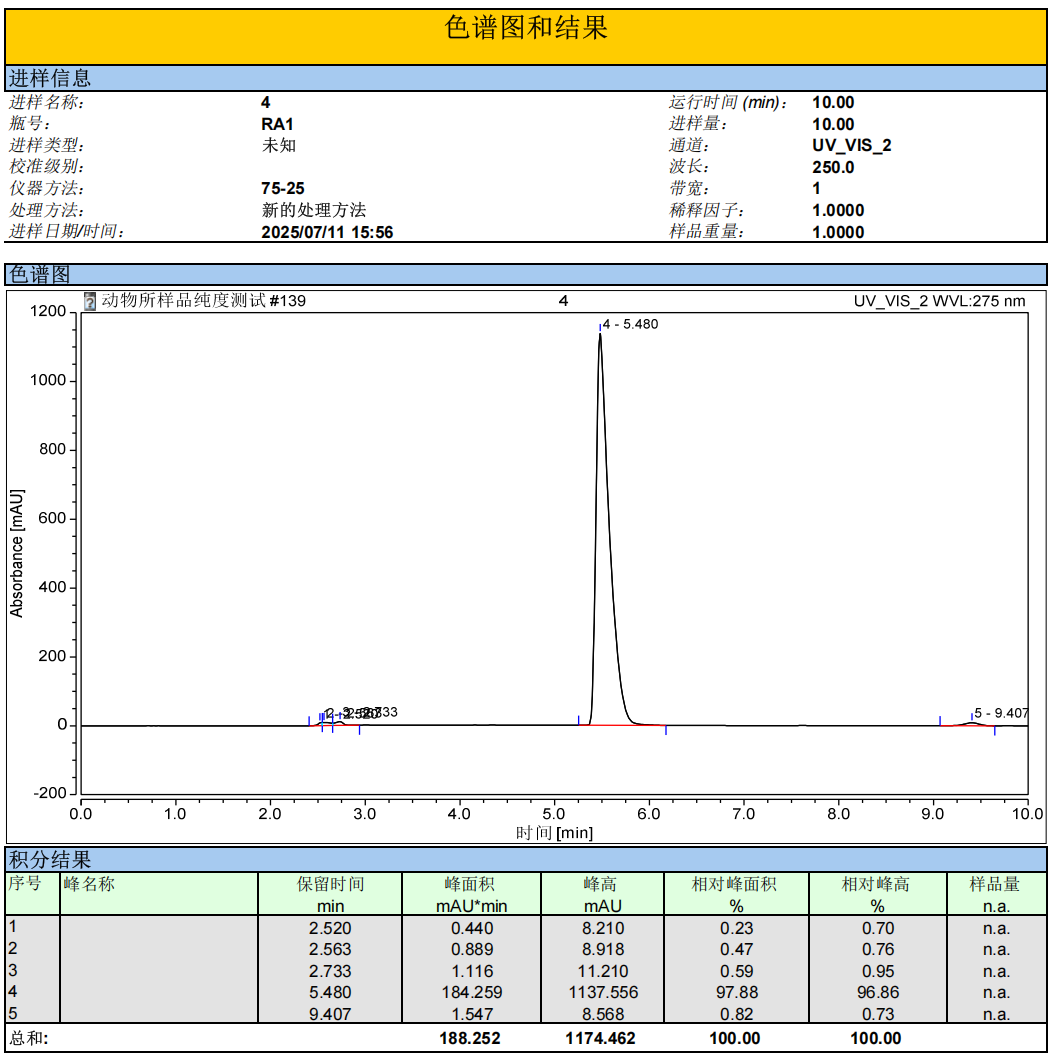


HPLC spectra of compound **4**


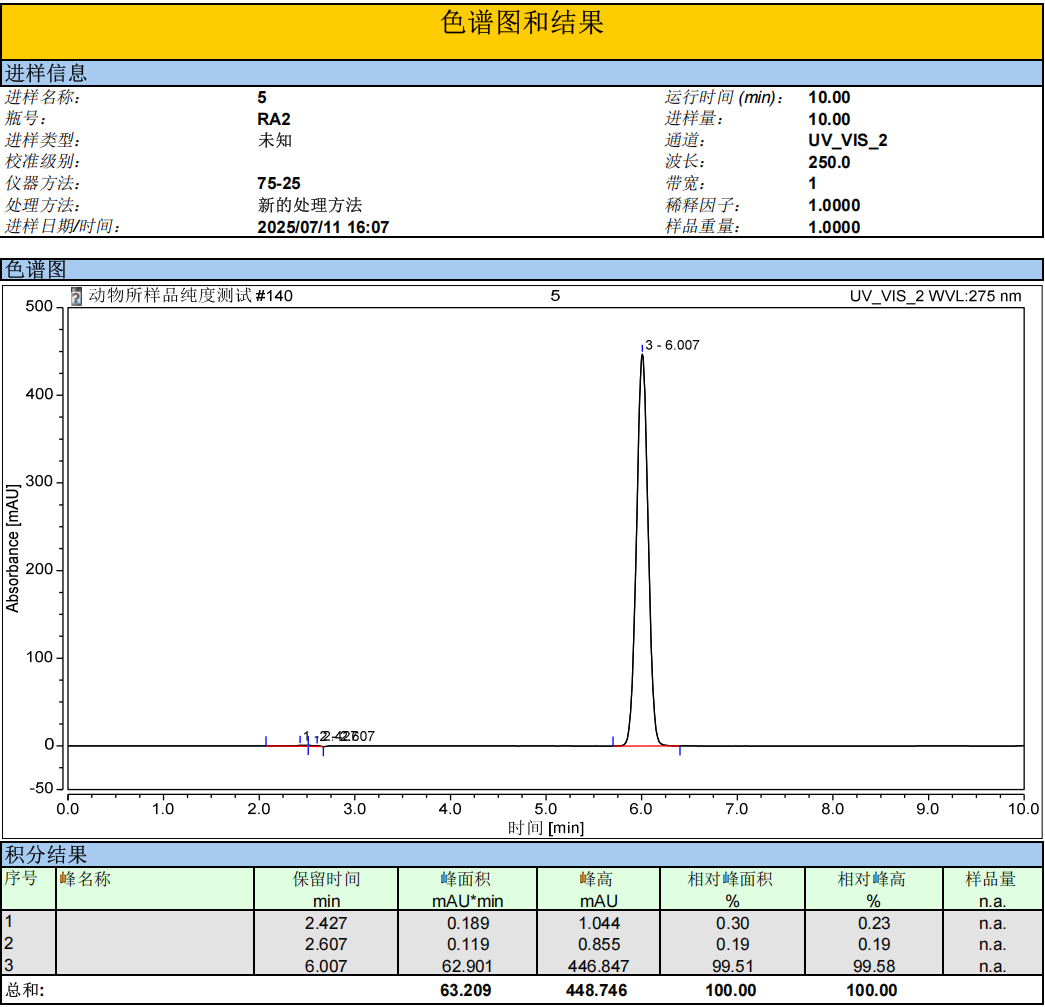


HPLC spectra of compound **5**


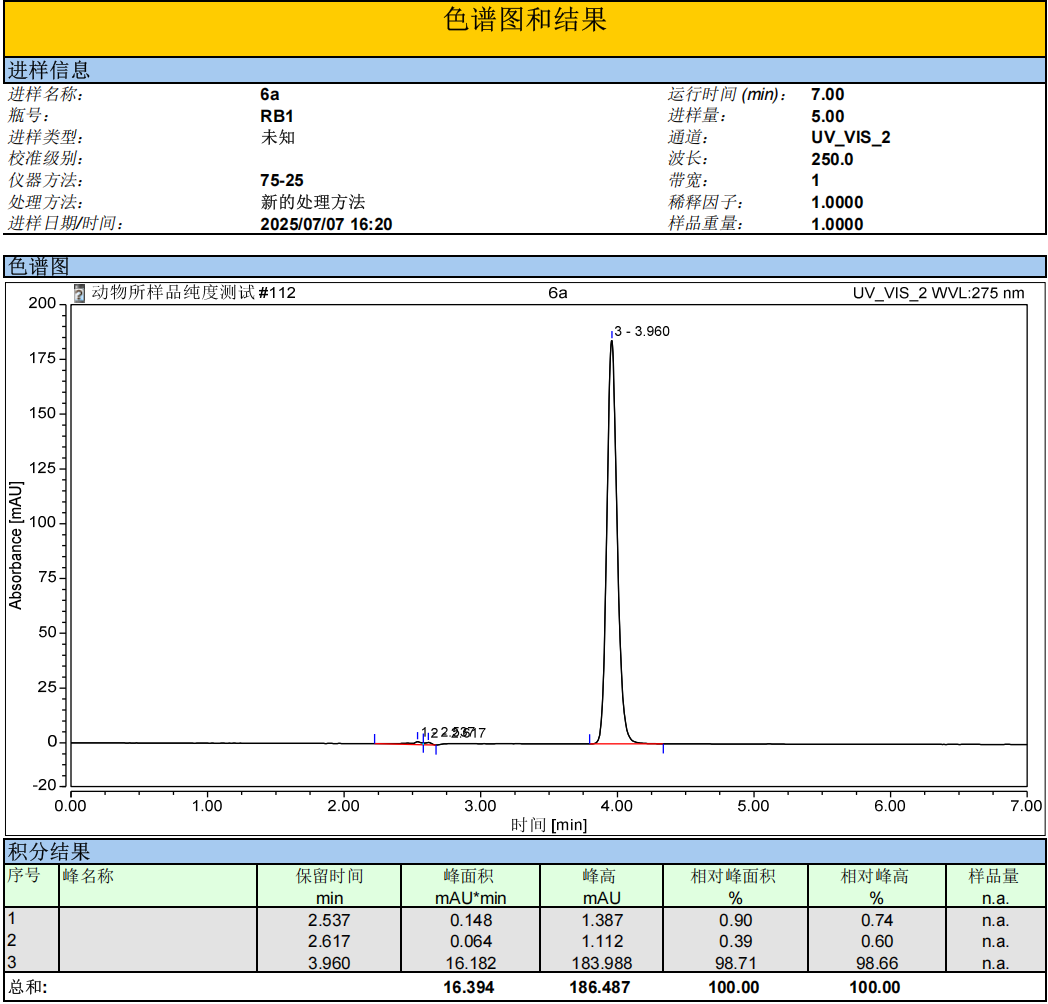


HPLC spectra of compound **6a**


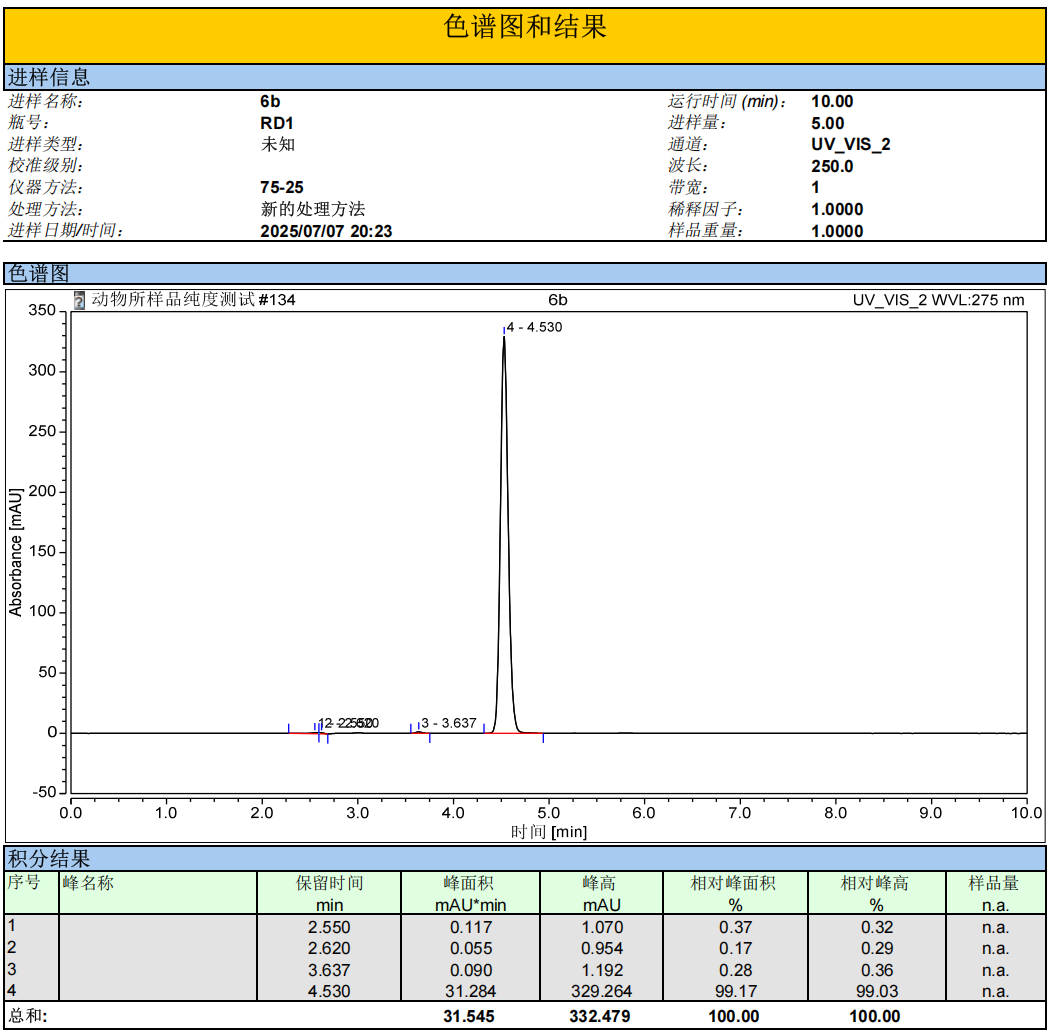


HPLC spectra of compound **6b**


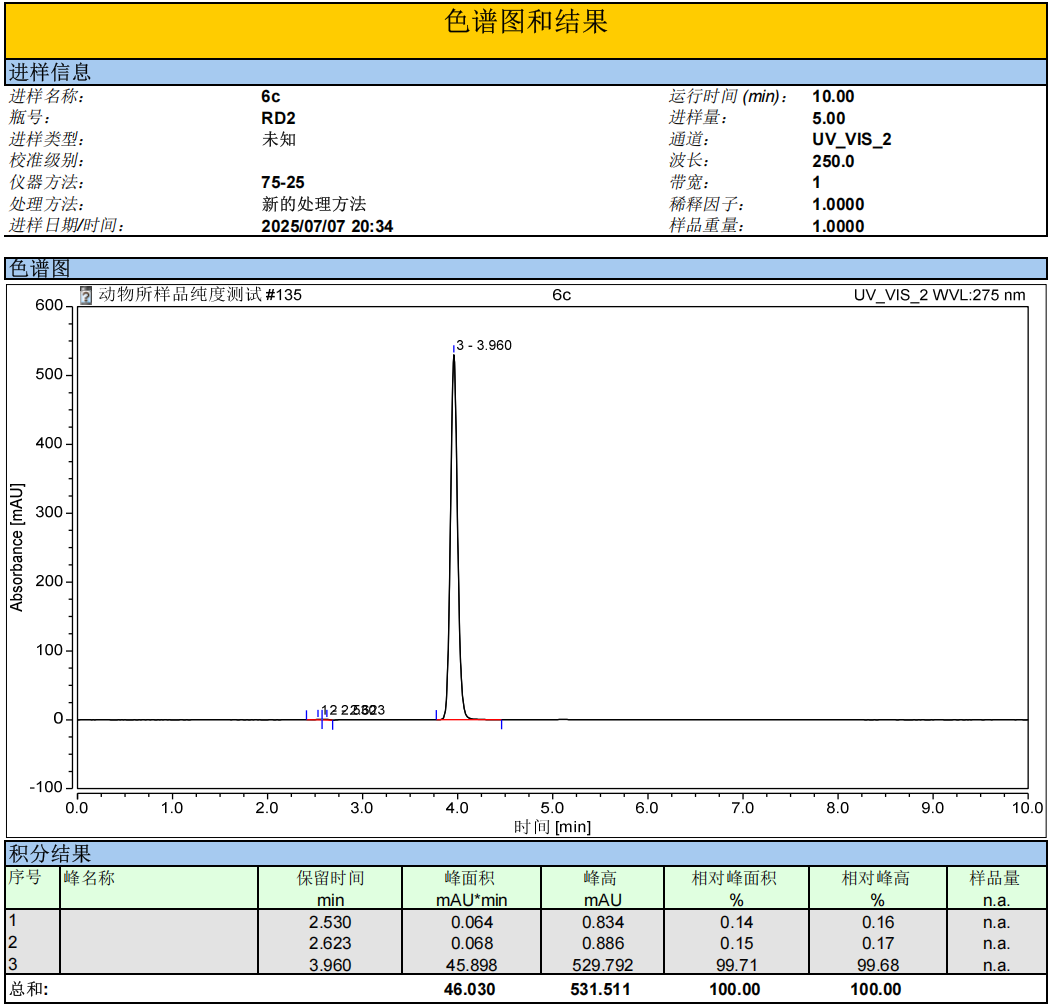


HPLC spectra of compound **6c**


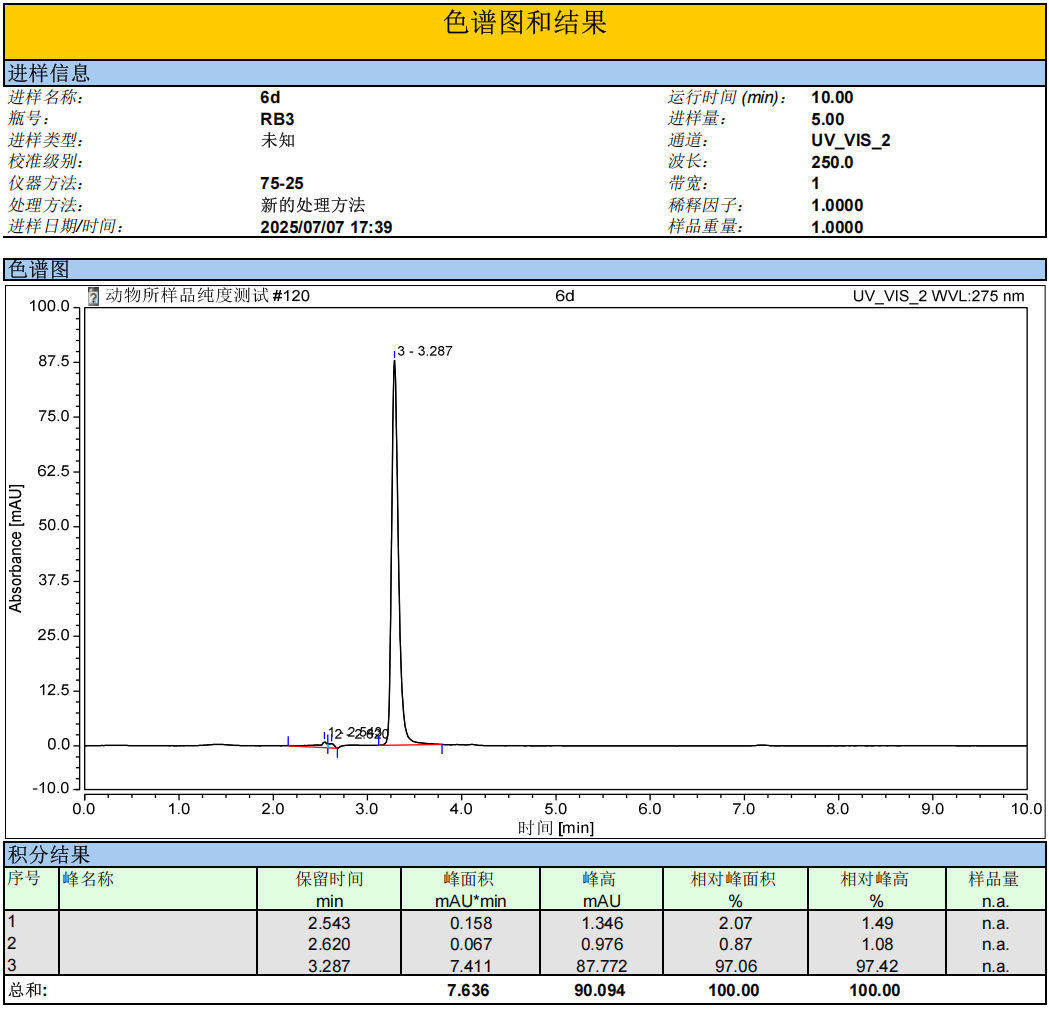


HPLC spectra of compound **6d**


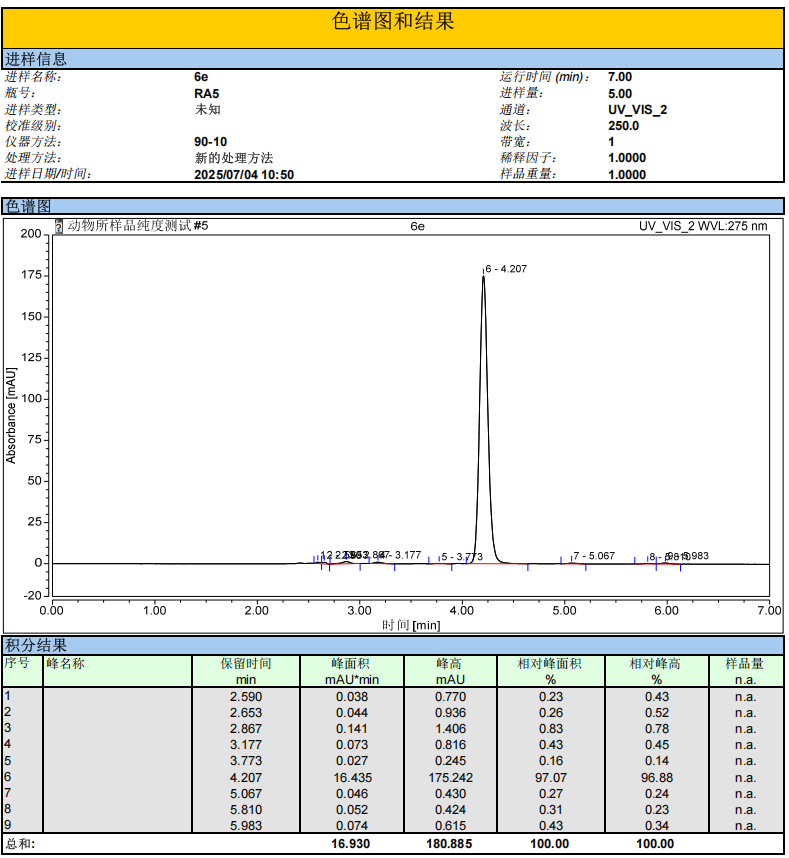


HPLC spectra of compound **6e**


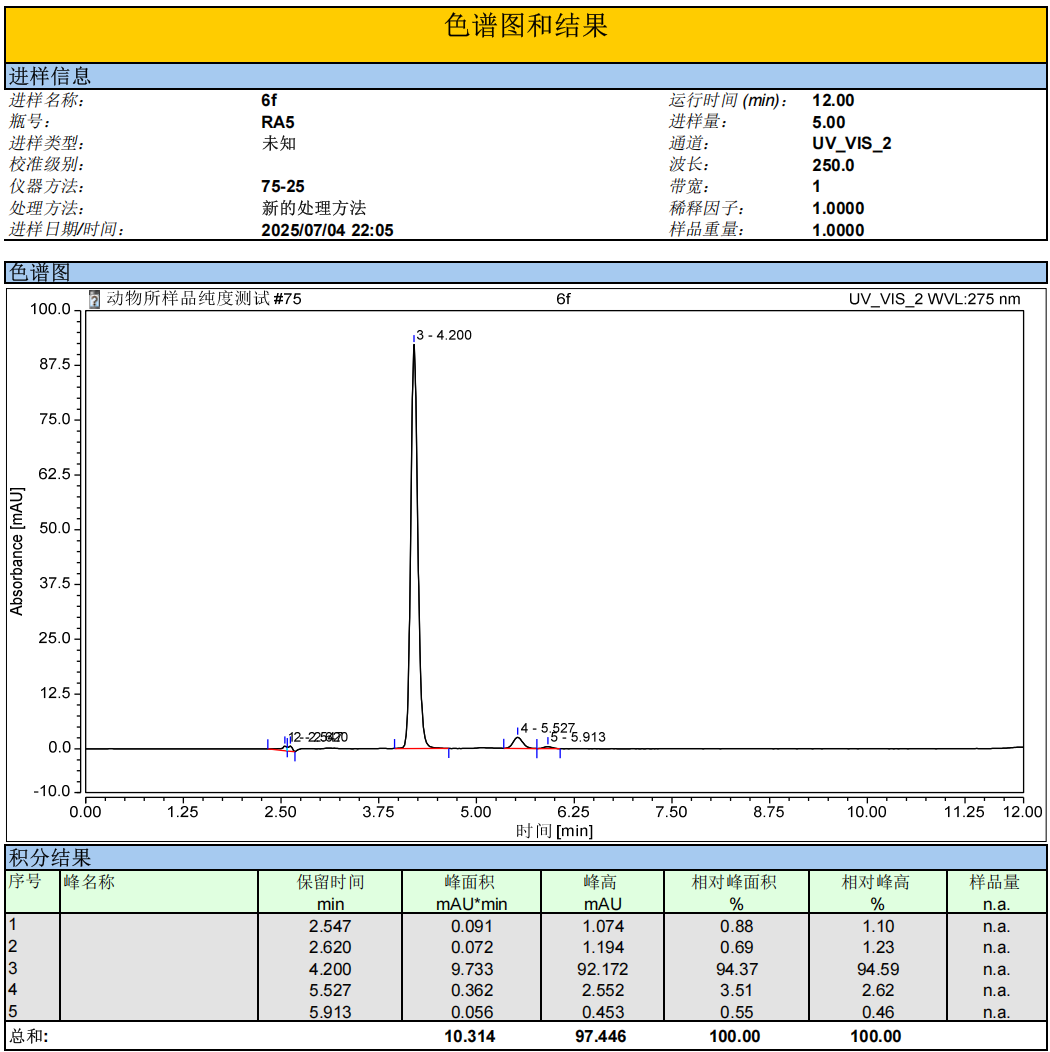


HPLC spectra of compound **6f**


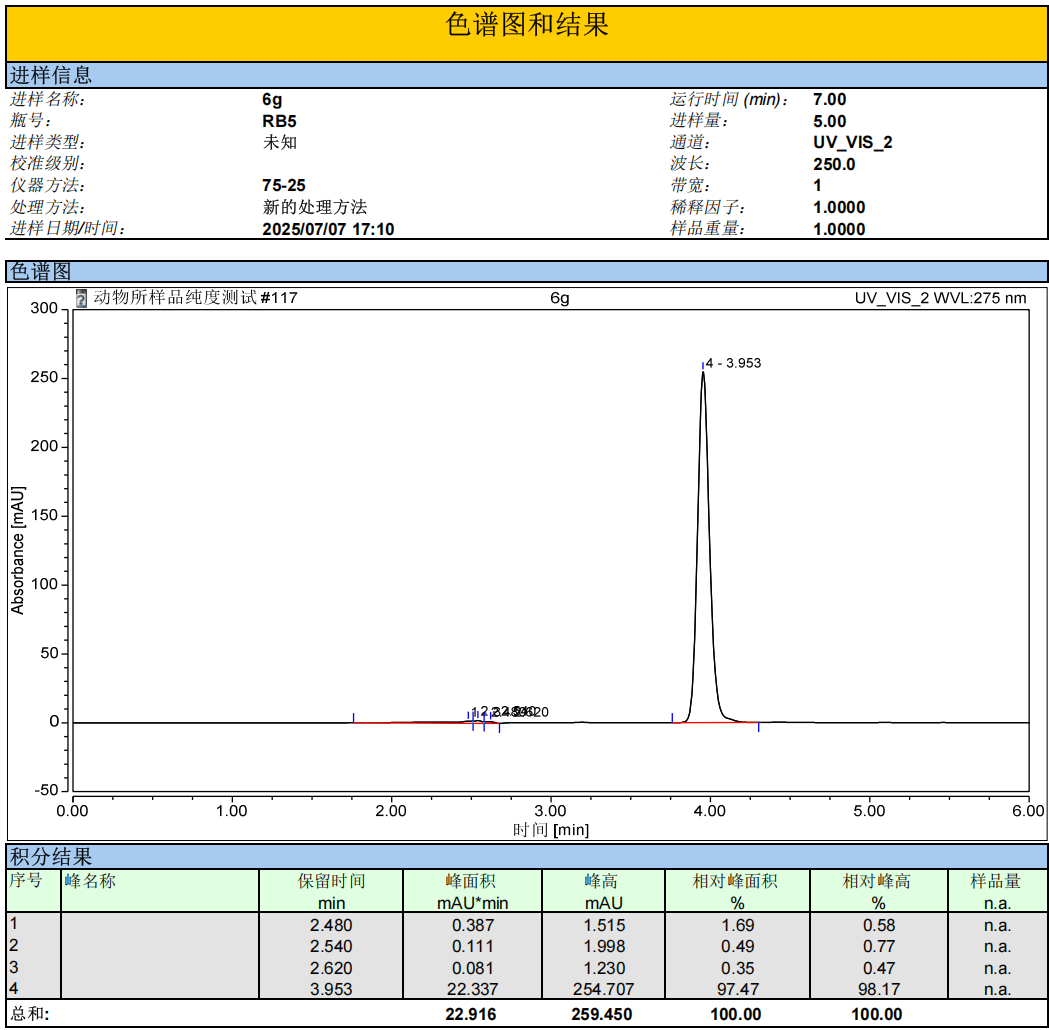


HPLC spectra of compound **6g**

HPLC spectra of compound **6h**

HPLC spectra of compound **6i**

HPLC spectra of compound **6j**

HPLC spectra of compound **6k**

HPLC spectra of compound **7**

HPLC spectra of compound **8**

HPLC spectra of compound **9a**

HPLC spectra of compound **9b**

HPLC spectra of compound **9c**

HPLC spectra of compound **9d**

HPLC spectra of compound **9e**

HPLC spectra of compound **9f**

HPLC spectra of compound **9g**

HPLC spectra of compound **9h**

HPLC spectra of compound **9i**

HPLC spectra of compound **9j**

HPLC spectra of compound **9k**

HPLC spectra of compound **9l**

Supplementary Tables

Table S1. The expression of TNF-α (Figure **3A**)

| **group** | **TNF-α** | **group** | **TNF-α** |
| --- | --- | --- | --- |
| **Control** | 1±0.02 | **6j** | 20.42±10.65 |
| **LPS** | 19.54±0.68 | **6k** | 14.81±3.5 |
| **Dex** | 12.96±2.57 | **7** | 17.7±0.98 |
| **Cel** | 11.11±0.13 | **8** | 12.01±5.96 |
| **4** | 71.21±4.13 | **9c** | 17.25±0.84 |
| **5** | 13.06±0.37 | **9d** | 14.68±0.41 |
| **6c** | 11.79±0.35 | **9e** | 15.15±1.15 |
| **6e** | 7.71±0.19 | **9g** | 17.95±1.61 |
| **6f** | 9.55±0.47 | **9h** | 19.79±0.89 |
| **6g** | 11.2±1.12 | **9j** | 68.04±2.07 |
| **6h** | 7.88±0.68 | **9k** | 85.43±1.7 |
| **6i** | 13.25±1.91 | **9l** | 144.39±2.63 |

Table S2. The expression of IL-6 (Figure **3B**)

| **group** | **IL-6** | **group** | **IL-6** |
| --- | --- | --- | --- |
| **Control** | 0.96±0.13 | **6j** | 270.29±24.29 |
| **LPS** | 208.94±10.85 | **6k** | 190.93±8.27 |
| **Dex** | 93.45±10.64 | **7** | 285.81±7.92 |
| **Cel** | 87.06±1.02 | **8** | 151.66±8.4 |
| **4** | 74.04±4.07 | **9c** | 182.25±11.98 |
| **5** | 243.45±10.2 | **9d** | 129.9±5.4 |
| **6c** | 64.82±10.86 | **9e** | 160.62±14.43 |
| **6e** | 74.64±8.39 | **9g** | 129.65±8.97 |
| **6f** | 591.82±74.72 | **9h** | 154.97±5.37 |
| **6g** | 130.31±29.3 | **9j** | 76.23±7.19 |
| **6h** | 216.52±19.46 | **9k** | 124.23±63.95 |
| **6i** | 133.03±8.75 | **9l** | 110.93±25.79 |

Table S3. The expression of IL-1β (Figure **3C**)

| **group** | **IL-1β** | **group** | **IL-1β** |
| --- | --- | --- | --- |
| **Control** | 1.01±0.13 | **6j** | 178.33±119.84 |
| **LPS** | 217.52±12.8 | **6k** | 100.2±4.06 |
| **Dex** | 48.91±3.05 | **7** | 294.75±17.35 |
| **Cel** | 111.73±2.06 | **8** | 344.48±54.46 |
| **4** | 66.08±0.08 | **9c** | 128.54±6.68 |
| **5** | 15.51±5.09 | **9d** | 68.49±5.92 |
| **6c** | 31.67±2 | **9e** | 154.49±8.56 |
| **6e** | 29.46±6.66 | **9g** | 28±0.1 |
| **6f** | 37.51±1.96 | **9h** | 105.24±0.36 |
| **6g** | 92.22±3.83 | **9j** | 98.2±2.31 |
| **6h** | 174.71±12.69 | **9k** | 103±2.12 |
| **6i** | 148.31±8.73 | **9l** | 129.71±3.52 |

Table S4. The expression of CD206 (Figure **3D**)

| **group** | **CD206** | **group** | **CD206** |
| --- | --- | --- | --- |
| **Control** | 1±0.02 | **6j** | 1.14±0.08 |
| **LPS** | 0.71±0 | **6k** | 0.76±0.15 |
| **Dex** | 1.03±0.02 | **7** | 0.95±0.03 |
| **Cel** | 0.76±0.08 | **8** | 0.95±0.09 |
| **4** | 0.81±0.06 | **9c** | 0.75±0.08 |
| **5** | 0.76±0.17 | **9d** | 1.15±0.05 |
| **6c** | 0.84±0.09 | **9e** | 0.58±0.07 |
| **6e** | 0.87±0.05 | **9g** | 0.72±0.15 |
| **6f** | 1.34±0.12 | **9h** | 0.78±0.19 |
| **6g** | 1.26±0.2 | **9j** | 0.69±0.05 |
| **6h** | 1.24±0.12 | **9k** | 1.01±0.07 |
| **6i** | 1.09±0.15 | **9l** | 1.02±0.11 |

Table S5. The expression of CD206 (Figure **3E**)

| **group** | **IL-10** | **group** | **IL-10** |
| --- | --- | --- | --- |
| **Control** | 1 | **6j** | 1.98±0.29 |
| **LPS** | 0.9±0.07 | **6k** | 0.46±0.03 |
| **Dex** | 1.6±0.11 | **7** | 1.25±0.14 |
| **Cel** | 1.13±0.08 | **8** | 0.93±0.17 |
| **4** | 1.18±0.04 | **9c** | 2.7±0.22 |
| **5** | 0.95±0.01 | **9d** | 2.31±0.02 |
| **6c** | 0.77±0.05 | **9e** | 0.43±0.04 |
| **6e** | 1.21±0.08 | **9g** | 0.84±0.23 |
| **6f** | 0.9±0.12 | **9h** | 1.32±0.32 |
| **6g** | 1.37±0.09 | **9j** | 0.88±0.04 |
| **6h** | 2.03±0.13 | **9k** | 0.64±0.08 |
| **6i** | 1.41±0.05 | **9l** | 1.17±0.06 |

Table S6. The expression of NF-κB (Figure **4D**)

| **group** | **NF-κB** |
| --- | --- |
| **Control** | 1±0.02 |
| **LPS** | 3.21±0.14 |
| **Dex** | 7.1±2.32 |
| **Cel** | 2.28±0.36 |
| **6g** | 1.88±0.2 |

Table S7. The expression of NF-κB (Figure **4E**)

| **group** | **LPA2** |
| --- | --- |
| **Control** | 0.99±0.05 |
| **LPS** | 0.58±0.12 |
| **Dex** | 1.39±0.03 |
| **Cel** | 1.07±0.09 |
| **6g** | 0.35±0.04 |

Table S8. The expression of SOD (Figure **7A**)

| **group** | **SOD** | **group** | **SOD** |
| --- | --- | --- | --- |
| **Control** | 0.86±0.06 | **6j** | 2.23±0.08 |
| **LPS** | 1.51±0.07 | **6k** | 1.59±0.02 |
| **Dex** | 1.75±0.06 | **7** | 2.03±0.03 |
| **Cel** | 2.08±0.75 | **8** | 1.71±0.03 |
| **4** | 1.44±0.02 | **9c** | 2.07±0.05 |
| **5** | 1.68±0.05 | **9d** | 1.87±0.03 |
| **6c** | 2.13±0.16 | **9e** | 1.81±0.04 |
| **6e** | 1.65±0.05 | **9g** | 1.93±0.02 |
| **6f** | 1.82±0.05 | **9h** | 1.87±0.09 |
| **6g** | 1.53±0.02 | **9j** | 1.81±0.08 |
| **6h** | 2.15±0.09 | **9k** | 1.59±0.04 |
| **6i** | 2.08±0.01 | **9l** | 1.95±0.16 |

Table S9. The expression of Nrf2 (Figure **7B**)

| **group** | **Nrf2** | **group** | **Nrf2** |
| --- | --- | --- | --- |
| **Control** | 1.01±0.03 | **6j** | 1.42±0.03 |
| **LPS** | 0.77±0.03 | **6k** | 0.9±0.03 |
| **Dex** | 0.9±0.01 | **7** | 0.99±0.01 |
| **Cel** | 0.47±0.02 | **8** | 0.88±0.02 |
| **4** | 0.81±0.03 | **9c** | 0.94±0.07 |
| **5** | 0.92±0.03 | **9d** | 0.9±0.03 |
| **6c** | 0.98±0.05 | **9e** | 0.89±0.03 |
| **6e** | 0.85±0.03 | **9g** | 1.03±0.05 |
| **6f** | 1.09±0.04 | **9h** | 0.89±0.02 |
| **6g** | 0.9±0.07 | **9j** | 0.96±0.01 |
| **6h** | 1.07±0.03 | **9k** | 0.78±0.02 |
| **6i** | 0.91±0.04 | **9l** | 1.04±0.02 |

Table S10. The expression of HO-1 (Figure **7C**)

| **group** | **HO-1** | **group** | **HO-1** |
| --- | --- | --- | --- |
| **Control** | 1±0.09 | **6j** | 1.06±0.02 |
| **LPS** | 0.96±0.03 | **6k** | 1.84±0.06 |
| **Dex** | 1.17±0.02 | **7** | 2.94±0.01 |
| **Cel** | 2.59±0.32 | **8** | 1.27±0.01 |
| **4** | 1.62±0.04 | **9c** | 1.33±0 |
| **5** | 1.71±0.01 | **9d** | 1.29±0.02 |
| **6c** | 1.59±0.05 | **9e** | 1.11±0.01 |
| **6e** | 1.82±0.04 | **9g** | 1.33±0.02 |
| **6f** | 1.76±0.02 | **9h** | 1.6±0.06 |
| **6g** | 1.35±0.01 | **9j** | 1.75±0.02 |
| **6h** | 1.25±0.03 | **9k** | 1.34±0.04 |
| **6i** | 1.1±0.02 | **9l** | 1.29±0.03 |

Table S11. The expression of NO (Figure **7D**)

| **group** | **NO** | **group** | **NO** |
| --- | --- | --- | --- |
| **Control** | 1±0 | **6j** | 1.5±0.1 |
| **LPS** | 1.67±0.13 | **6k** | 1.26±0.07 |
| **Dex** | 1.32±0.06 | **7** | 1.3±0.01 |
| **CEL** | 0.68±0 | **8** | 1.46±0.07 |
| **4** | 1.24±0.05 | **9c** | 1.47±0 |
| **5** | 1.27±0.02 | **9d** | 1.54±0.1 |
| **6c** | 1.41±0.1 | **9e** | 1.71±0.14 |
| **6e** | 1.34±0.02 | **9g** | 1.44±0.07 |
| **6f** | 1.32±0.03 | **9h** | 1.45±0.1 |
| **6g** | 1.63±0 | **9j** | 1.22±0.04 |
| **6h** | 1.53±0.1 | **9k** | 1.37±0.04 |
| **6i** | 1.47±0.06 | **9l** | 1.55±0.01 |
